# ReTrOS: a MATLAB toolbox for reconstructing transcriptional activity from gene and protein expression data

**DOI:** 10.1186/s12859-017-1695-8

**Published:** 2017-06-26

**Authors:** Giorgos Minas, Hiroshi Momiji, Dafyd J. Jenkins, Maria J. Costa, David A. Rand, Bärbel Finkenstädt

**Affiliations:** 1 Systems Biology Centre, University of Warwick, Coventry, CV34 4HD UK; 2 Department of Statistics, University of Warwick, Coventry, CV34 4HD UK; 3 Mathematics Institute, University of Warwick, Coventry, CV34 4HD UK; 4 School of Life Sciences, University of Warwick, Coventry, CV34 4HD UK

**Keywords:** Gene transcription, Time series, Transcriptional switches, Circadian timing

## Abstract

**Background:**

Given the development of high-throughput experimental techniques, an increasing number of whole genome transcription profiling time series data sets, with good temporal resolution, are becoming available to researchers. The ReTrOS toolbox (Reconstructing Transcription Open Software) provides MATLAB-based implementations of two related methods, namely ReTrOS–Smooth and ReTrOS–Switch, for reconstructing the temporal transcriptional activity profile of a gene from given mRNA expression time series or protein reporter time series. The methods are based on fitting a differential equation model incorporating the processes of transcription, translation and degradation.

**Results:**

The toolbox provides a framework for model fitting along with statistical analyses of the model with a graphical interface and model visualisation. We highlight several applications of the toolbox, including the reconstruction of the temporal cascade of transcriptional activity inferred from mRNA expression data and protein reporter data in the core circadian clock in *Arabidopsis thaliana*, and how such reconstructed transcription profiles can be used to study the effects of different cell lines and conditions.

**Conclusions:**

The ReTrOS toolbox allows users to analyse gene and/or protein expression time series where, with appropriate formulation of prior information about a minimum of kinetic parameters, in particular rates of degradation, users are able to infer timings of changes in transcriptional activity. Data from any organism and obtained from a range of technologies can be used as input due to the flexible and generic nature of the model and implementation. The output from this software provides a useful analysis of time series data and can be incorporated into further modelling approaches or in hypothesis generation.

**Electronic supplementary material:**

The online version of this article (doi:10.1186/s12859-017-1695-8) contains supplementary material, which is available to authorized users.

## Background

Analyzing the temporal dynamics of mRNA and protein expression is a key ingredient to the study of gene function within the cell. The widespread adoption of high-throughput transcriptomic and proteomic technologies, such as microarrays, fluorescent imaging, transcriptional reporter constructs and sequencing, has enabled the generation of large numbers of high-resolution genome-scale time series data sets. The processing, analysis and summarising of such time series data has a number of theoretical and computational difficulties to overcome. If, for example, a protein reporter construct is used, what is the relationship between the observed reporter protein and the mRNA expression dynamics of the gene of interest? Moreover, allowing for the process of mRNA and protein degrading, what is the actual transcriptional activity?

Here, we present a software toolbox called ReTrOS (Reconstructing Transcription Open Software) which provides several approaches for processing and analysing both gene or protein expression time series data sets, with an easy-to-use graphical interface for user interaction. The software is written in the cross-platform MATLAB®; environment. The approach used in ReTrOS is based on a differential equation model [1] to account for transcription and degradation of mRNA molecules and translation and degradation of protein molecules. The model has been the basis to a number of novel methodologies to study the dynamics of gene expression [2–6]. The ReTrOS software currently provides two methods for the processing, reconstruction and analysis of gene transcription dynamics:

- *ReTrOS-Smooth*: based upon the algorithm introduced in Harper et al. (2011) [2]. ReTrOS-Smooth outputs a smooth reconstruction of transcription activity from a non-parametric representation of the transcriptional process. The algorithm is extended to incorporate both variability of measurement error and uncertainty about model parameters with credibility envelopes simulated through a bootstrap procedure.
- *ReTrOS-Switch*: based upon the algorithm introduced in Jenkins et al. (2013) [3]. ReTrOS-Switch outputs a reconstruction of transcriptional activity using a temporal “switch” model where transcription rates are subject to temporal jumps at unknown time points. A Bayesian inference algorithm using reversible jump Markov Chain Monte Carlo is provided for modeling mRNA data as in [3], and has, for the purpose of this article, been extended to model protein dynamics.

This article introduces and describes the underlying model and the two algorithms implemented in ReTrOS. We briefly discuss the data requirements and compare the applicability of the two methods. Following the methods overview, we present several cases of applying ReTrOS concluding with some final remarks summarising the software and its uses.

## Implementation

### mRNA and protein expression dynamics

The basic model underlying ReTrOS is an ordinary differential equation (ODE) model introduced in [1]:

1 $$\begin{array}{@{}rcl@{}} \frac{\mathrm{d}M}{\mathrm{d}t}&=& \tau(t) - \delta_{M} M(t) \end{array} $$

2 $$\begin{array}{@{}rcl@{}} \frac{\mathrm{d}P}{\mathrm{d}t}&=& \alpha M(t) - \delta_{P} P(t) \end{array} $$

where *M*(*t*) is the amount of mRNA transcript at time *t*, *P*(*t*) is the amount of protein at time *t*, *τ*(*t*) is the rate of transcription/mRNA synthesis, *δ*_*M*_ is the rate of mRNA transcript decay, *α* is the rate of translation/protein synthesis and *δ*_*P*_ is the rate of protein decay.

Let *y*(*t*_*i*_), *i*=1,…,*n*, be the discretely observed (not necessarily at equidistant time points) imaging protein time series. Assuming that the mean of the data are proportional with unknown factor *κ* to the concentration of the reporter protein, we have

3 $$ y(t_{i}) = \tilde{P}(t_{i}) + \epsilon(t_{i}), \text{ where } \tilde{P}(t)= \kappa P(t),  $$

and *ε*(*t*_*i*_), *i*=1,…,*n* are independent normally distributed random variables each with mean zero and unknown variance *σ* ^2^(*t*_*i*_). If the observations are mRNA expression levels (e.g. measured using microarrays technology), instead of (Eq. 3) we have

4 $$ y(t_{i}) = \tilde{M}(t_{i}) + \epsilon(t_{i}), \text{where} \tilde{M}(t)= \kappa M(t),  $$

with *ε*(*t*_*i*_), *i*=1,…,*n* again independent normally distributed random variables each with mean zero and unknown variance *σ* ^2^(*t*_*i*_).

The main difference between ReTrOS-Smooth and ReTrOS-Switch lies in the formulation of the transcription function *τ*(*t*) as follows.

### ReTrOS-smooth: reconstruction of a smooth transcription function

Here we use a kernel smoothing function that we found to be more flexible outperforming the spline approach particularly when the data changes rapidly over short time-scales. The user is assumed to have prior information about the values of the degradation rates and has to provide these through appropriate distributions. The algorithm extends the transcription reconstruction algorithm originally introduced in [2] by incorporating both variability of measurement error and model parameter uncertainty through a bootstrap procedure.

Rewriting (Eq. 2) in terms of $\tilde {P}(t)$ we first note that, for a given solution path $\tilde {P}(t)$ and degradation rate *δ*_*P*_ we can compute the mRNA path by

5 $$ \tilde{M}(t):=\kappa \alpha M(t) = \frac{d\tilde{P}(t)}{dt} + \delta_{P} \tilde{P}(t).  $$

In ReTrOS-Smooth, the continuous function $\tilde {P}(t)$ is replaced using kernel smoothing [7] with a bandwidth optimized by leave–one–out cross–validation. One can then recover the transcriptional dynamics by inserting the estimated $\tilde {M}(t)$ into

6 $$ \tilde{\tau}(t):=\kappa \alpha \tau(t) = \frac{d\,\tilde{M}(t)}{dt} + \delta_{M} \tilde{M}(t)\,.  $$

If the scaling factors *κ* and *α* are unknown, the profile of the reconstructed time paths representing the mRNA and transcriptional dynamics are computed at an arbitrary level. In ReTrOS we set *κ* *α*=1 as default parameter values.

This two-step back-calculation is applied if the observed time courses represent protein data arising, for example, from reporter protein or fluorescently tagged functional protein. If the data *y*(*t*_*i*_) represent mRNA levels, the procedure involves only the one step of the back-calculation in (Eq. 6) and instead of (Eq. 3), *y*(*t*_*i*_) are assumed to be related to the unknown mRNA expression levels, *M*(*t*), through (Eq. 4). A prior distribution for the mRNA degradation rate, *δ*_*M*_, is provided by the user here. The next paragraph details the steps of the ReTrOS-Smooth implementation algorithm.

**Variability of the reconstructed profile** The variability of the profiles for $\tilde {\tau }(t)$ depends on the variance of the fitted kernel function given the sample size and variance of the residuals. ReTrOS-Smooth incorporates uncertainty about knowledge of the degradation rate parameters. Let $\hat {\epsilon }_{t_{i}}= \hat {y}(t_{i})-y(t_{i})$ denote the residuals between the data and the fitted smooth kernel function at time *t*_*i*_. The influence of both sources of variability can be estimated in a straightforward way via bootstrap simulation methods [8]:

- (1) Compute $\hat {y}(t)$ using kernel smoothing with a bandwidth optimized by leave–one–out cross–validation. In order to estimate a model for the variance we apply kernel estimation at the same bandwidth to fit a smooth function $\hat {\sigma }^{2}(t)$ to the squared residuals $\hat {\epsilon }_{i}^{2}$.
- (2) Obtain a resampled data profile of same sample size $y^{*}(t_{i})=\hat {y}(t_{i})+e(t_{i})$, *t*_*i*_=1,…,*n* where $e(t_{i})\sim N(0,\hat {\sigma }^{2}(t_{i}))$, i.e. using the variance function evaluated at *t*_*i*_, and such that *e*(*t*_*i*_) has the same sign as $\hat {\epsilon }_{t_{i}}$.
- (3) Find $\hat {y}^{*}(t)$ using kernel smoothing at the same bandwidth as used in step 1.
- (4) Draw values for *δ*_*P*_ and *δ*_*M*_ from their prior distributions.
- (5) Calculate $\tilde {M}$ and $\tilde {\tau }$ using (Eq. 5) and (Eq. 6), respectively.

**Fig. 1:**
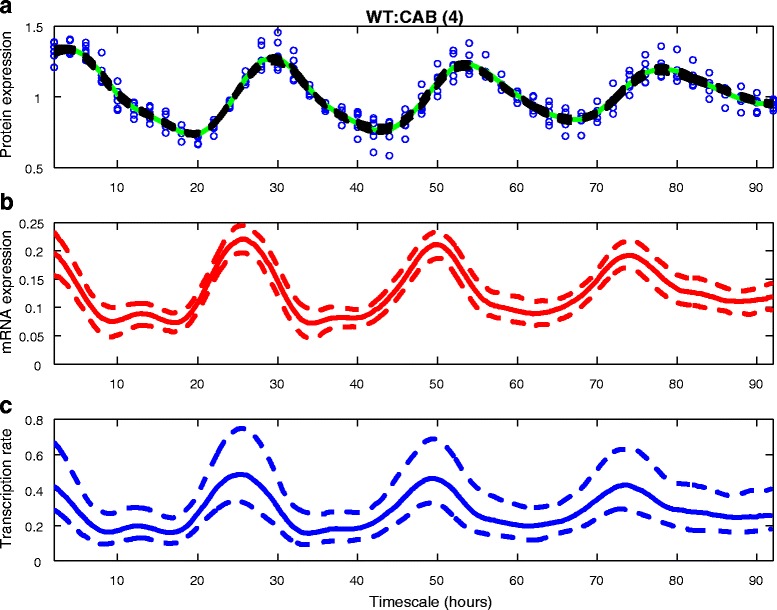
Example output from ReTrOS-Smooth applied to protein data. Data is from a luciferase reporter for the CAB clock gene in wild-type Arabidopsis plants (ROBuST Project) [15]. Panel **a**) shows the raw input data (*blue circles*), the kernel-smoothed input data (*green*) and the median fitted protein output (*black*). Panel **b**) shows the back-calculated median reporter mRNA expression (*red*) with 2.5% and 97.5% percentiles in *dashed lines*. Panel **c**) shows the back-calculated median transcription activity (*blue*) with the same percentiles as (**b**). The model in Eqs. (5) and (6) are used here. Degradation rates (mean, std. dev.) of mRNA and protein are (2.3, 0.46) and (0.13, 0.010), respectively, set by choosing *luc reporter* in the parameter setting GUI (Fig. 5, *upper panel*). For other parameter values, see the middle panel of Fig. 5

Steps (2) to (5) are repeated *R* times (by default set to 99 in ReTrOS-Smooth), after which point-wise mean estimates for $\tilde {M}$ and $\tilde {\tau }$ are plotted along with estimated 95% confidence envelopes for the reconstructed curves. The values of *δ*_*P*_ and *δ*_*M*_ in step (4) are drawn from a gamma distribution with mean and standard deviation provided by the user. If the time course represents mRNA levels then steps (1) to (5) are implemented analogously where only a prior distribution for *δ*_*M*_ is required. An example output from the ReTrOS-Smooth method is shown in Fig. 1.

### ReTrOS-switch: reconstruction of a switching transcription profile

This method is an implementation of the transcriptional switch model inference introduced in Finkenstädt et al. [1] and Jenkins et al. [3] where it was applied to mRNA time series data. Here it is further extended to include protein dynamics through (Eq. 2). We assume that the transcriptional rate function *τ*(*t*) in (Eq. 1) has the profile of a step function where the rate is constant between time points, *s*_*i*_, where it changes due to unobserved transcriptional events (for example, activation or inhibition):

7 $$ \tau(t)= \tau_{i} \text{ for } s_{i} < t \leq s_{i+1}, i=1,\ldots,k,\; t \in [0, L]  $$

with *L* the total length of the time interval observed. Note that transcription might not be fully turned off and that there may be more than just two states. The posterior distributions for the switch-times *s*_1_,…,*s*_*k*_ and number of switches *k* are estimated by the software. Extending this model to incorporate protein expression dynamics (Eq. 2) gives the solution:

8 $$ P(t)=P(0) \mathrm{e}^{-\delta_{P} t} + \alpha \mathrm{e}^{-\delta_{P} t} \int_{0}^{t} \mathrm{e}^{\delta_{P} u} M(u)\: \mathrm{d}u  $$

with *M*(*t*) satisying (Eq. 5) and *τ*(*t*) as in (Eq. 7).

A fast estimation algorithm can be implemented due to the fact that the ODE system, for given values of the degradation parameters during an iteration of the estimation sampler, can be written as a linear model for functions of the parameters *P*(0),*M*(0) and all *τ*. That is, (Eq. 8) can be written as a linear regression model, which for the protein is of the form:

9 $$ P(t) = \beta_{0} X^{P}_{0} + \beta_{1} X^{P}_{1} + \beta_{2} X^{P}_{2} + \dots + \beta_{k+2} X^{P}_{k+2}   $$

where $\beta _{0}=P(0), \beta _{1}= M(0), \beta _{2} = \frac {\tau _{0}}{\delta _{M}}, \beta _{3}= \frac {\tau _{1}-\tau _{0}}{\delta _{M}}, \ldots \,., \beta _{k+2} = \frac {\tau _{k}-\tau _{k-1}}{\delta _{M}}$ and the regressors are

$$\begin{array}{@{}rcl@{}} X^{P}_{0}&=&\mathrm{e}^{-\delta_{P} t} \\ X^{P}_{1}&=&\frac{\alpha}{\delta_{P}-\delta_{M}}\left(\mathrm{e}^{-\delta_{M} t} - \mathrm{e}^{-\delta_{P} t}\right) \\ X^{P}_{2}&=&\alpha\left(\frac{1-\mathrm{e}^{-\delta_{P} t}}{\delta_{P}} - \frac{\mathrm{e}^{-\delta_{M} t} - \mathrm{e}^{-\delta_{P} t}}{\delta_{P}-\delta_{M}}\right)\\ &\vdots&\\ X^{P}_{k+2}&=&\alpha\left(\frac{1-\mathrm{e}^{-\delta_{P} (t-s_{k})}}{\delta_{P}} - \frac{\mathrm{e}^{-\delta_{M} (t-s_{k})} - \mathrm{e}^{-\delta_{P} (t-s_{k})}}{\delta_{P}-\delta_{M}} \right) \end{array} $$

and, analogously, for the mRNA [3]

10 $$ M(t) = \beta_{1} X^{M}_{1} + \beta_{2} X^{M}_{2} + \dots + \beta_{k+2} X^{M}_{k+2}   $$

with

$$\begin{array}{*{20}l} & X^{M}_{1}= \mathrm{e}^{-\delta_{M} t}, X^{M}_{2} = 1 - \mathrm{e}^{-\delta_{M} t},\\ & X^{M}_{3} = 1 - \mathrm{e}^{-\delta_{M} (t - s_{1})}, \dots, X^{M}_{k+2} = 1 - \mathrm{e}^{-\delta_{M} (t - s_{k})}. \end{array} $$

**Parameter estimation and model output** Parameter estimation is performed using a Markov Chain Monte Carlo (MCMC) sampler following the methodological approach introduced in [3]. Like for ReTrOS-Smooth the user is required to provide prior means and standard deviations so that an informative Gamma distribution can be formulated for the degradation rates, *δ*_*P*_ and *δ*_*M*_, which are updated using a standard Metropolis-Hastings acceptance scheme. For protein reporter observations (Eq. 3) is used while (Eq. 4) is used for mRNA level observations, where the translation rate parameters, *α* and *κ*, if unknown, are fixed to 1 as in the ReTrOS-Smooth model.

The number and positions of the switch points are sampled using the reversible jump methodology [9]. Estimates of the values for the *β*_0_ to *β*_*k*+2_ coefficients, representing P(0), M(0) and *τ*_0_,…,*τ*_*k*_, are obtained through linear regression of the model (Eq. 9) given *δ*_*P*_ and *δ*_*M*_, protein expression data and the design matrix *X* ^*P*^. The regression can be performed using ordinary least squares or a parametric weighted least squares method (see, for example, [10] for details) with weights, $w_{e^{(i)}} = \hat {M}_{e^{(i)}}$, an estimate of the given expression at time *t*_*i*_, derived by a smoothing spline kernel. If mRNA expression data are provided, the reduced model given in (Eq. 10) can be inferred using *δ*_*M*_ and the *X* ^*M*^ design matrix only.

**Fig. 2:**
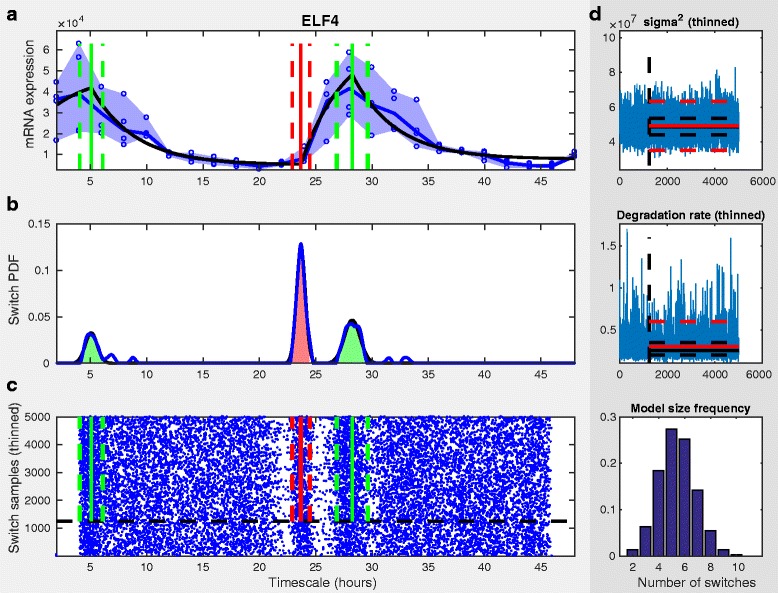
Example output summary from ReTrOS-Switch applied to mRNA microarray data. Data is from a microarray time-series for the ELF4 clock gene in wild-type Arabidopsis plants (PRESTA project) [12]. Panel **a**) shows the raw input data (*blue circles*, extremes of replicates shown in shaded region and median shown with line), the fitted mRNA expression (*black line*) and the estimated switch events (*μ*±1.96*σ* as vertical *red/green lines*). Panel **b**) shows the baseline-removed estimated switch time probability density (*black line*) and the estimated switch times Gaussian mixture model (*blue line*, with shaded *red/green* region indicating *μ*±1.96*σ*). Panel **c**) shows the accepted switch time samples, along with the switch events (*red/green*). The burn-in period of the chain is shown by the *dashed black line*. Panel **d**) shows the accepted samples from the precision (*σ* ^2^), *δ*_*m*_ and *δ*_*p*_ parameter chains in *blue* and a histogram of the accepted model size. Summaries of the parameter chains are shown in *black* (median and lower/upper quartiles) and *red* (*μ* and 1.96 *σ*). The model in Eq. (10) is used here. The default switch parameter values in the parameter setting GUI are used, with the prior mRNA degradation rate (mean = 1.5, std. dev. = 0.18), set by choosing *Arabidopsis* (see Fig. 5, *upper panel*)

**Fig. 3:**
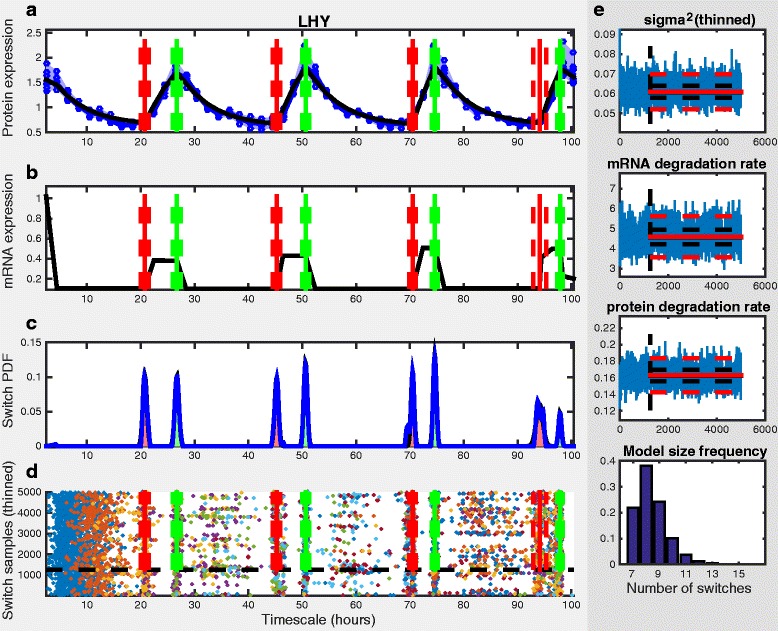
Example output summary from ReTrOS-Switch applied to protein data. Data is luciferase tagged protein for the LHY clock gene in wild-type Arabidopsis plants (ROBuST Project) [15]. Panel **a**) shows the raw input data (*blue circles*, extremes of replicates shown in shaded region and median shown with line), the fitted protein expression (*black line*) and the estimated switch events (*μ*±1.96*σ* as *vertical red/green lines*). Panel **b**) shows the back-calculated mRNA expression profiles (*black line*) with the estimated switch times. Panel **c**) shows the baseline-removed estimated switch time probability density (*black line*) and the estimated switch times Gaussian mixture model (*blue line*, with shaded red/green region indicating *μ*±1.96*σ*). Panel **d**) shows the accepted switch time samples, along with the switch events (*red/green*). The burn-in period of the chain is shown by the *dashed black line*. Panel **e**) shows the accepted samples from the precision (*σ* ^2^), *δ*_*m*_ and *δ*_*p*_ parameter chains in *blue* and a histogram of the accepted model size. Summaries of the parameter chains are shown in *black* (median and lower/upper quartiles) and *red* (*μ* and 1.96 *σ*). If mRNA expression data is used, specific output panels are removed accordingly. The model in Eq. (9) is used here. The default switch parameter values in the parameter setting GUI are used, with the prior mRNA and protein degradation rates set by choosing *luc reporter*

Given the nature of the sampling methodology, we have to summarise the posterior distributions obtained from the simulated chains. Univariate parameter distributions are summarised in terms of percentiles, whilst the switch time distribution is summarised as a Gaussian mixture model, such that each estimated switch event has a mean and a standard deviation derived from the marginal switch time probability density (see [3] for further details). In addition to summarising the sampled distributions across all model sizes, the sampled switch time density for specific model sizes are also shown in order of sampled frequency. Example output summary from the ReTrOS-Switch method applied to microarray mRNA expression data is shown in Fig. 2 and to protein reporter (luciferase) data in Fig. 3.

### Running ReTrOS

The ReTrOS toolbox provides both a graphical and a command-line interface for selecting input data files and specifying algorithm parameters. Users can either run the analysis directly from the graphical user interface, or alternatively from the command-line or batch scripts generated from the GUI.

**Fig. 4:**
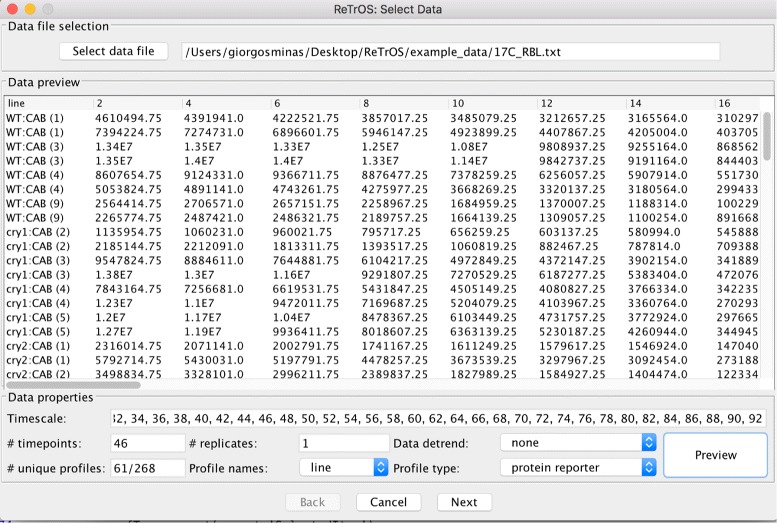
The ReTrOS GUI environment for importing data. The user can import data by selecting the data file directory of a spreadsheet file that is in the same format as in the example data provided with our software. The software automatically detects the number of time-points and replicates, the profiles with unique names, the column that defines the name of each gene/protein. The profile type can be defined by the user as either mRNA or protein reporter (for more details see ReTrOS User Manual)

**Input data format** The data format used is a simple table generated by standard spreadsheet software. A header row defines the observation time and each row contains the observed data for a single sample, gene or protein. The *N* columns in the table can be used to specify the name of the gene/protein or other annotation data and the remaining columns contain the observed values for the corresponding observation time in the header row (see Fig. 4). Replicate samples can be either treated as individual time series, or combined together.

**Fig. 5:**
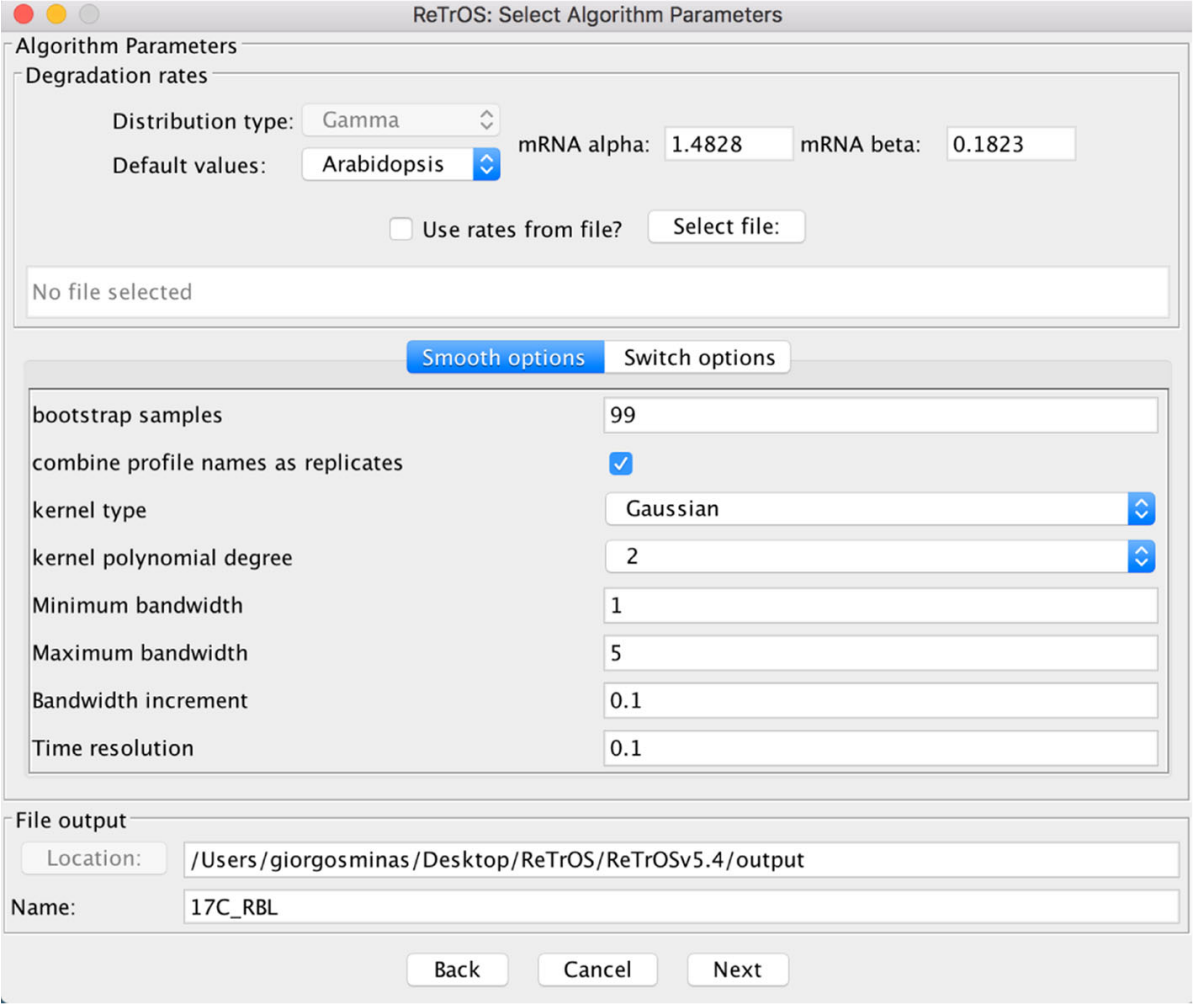
The GUI environment for selecting parameter values of ReTrOS-Smooth. Default parameter values are available to the user. The rate parameter values can also be imported by selecting the directory of the corresponding file. A similar GUI environment is available for the ReTrOS-Switch method (for more details see ReTrOS User Manual)

**Algorithm parameters** A range of user-definable parameters are available for both methods. The user interface provides default values for required parameters (such as number of iterations or bootstrap samples to run) and each method provides default values for all other parameters. Most algorithmic parameters can be modified through the user interface and all parameters can be modified through the batch scripts (see Fig. 5).

### Algorithm comparison

Whilst the two algorithms in ReTrOS use the same underlying transcription/translation model, there are a number of considerations as to which method may be more appropriate to use. The ReTrOS-Smooth method does not require, but can handle, replicate data and due to the non-parametric approach has fewer restrictions on data requirements. Model fitting using the ReTrOS-Switch method is improved by the use of replicate data samples, but requires that the replicate data samples are reasonably synchronised (both in expression range and dynamics) and that changes in transcription activity are expected. The algorithmic complexity of the ReTrOS-Smooth method is lower than for the ReTrOS-Switch method, which also requires a large number of MCMC iterations to estimate the parameter distributions accurately. As such, the computational time required for the ReTrOS-Smooth method is far smaller than for the ReTrOS-Switch method. ReTrOS-Smooth processes time series such as in Fig. 1 (4 replicates of time-series of 46 data-points) with the default parameter setting (see Fig. 5) in about 20 s (3.1-GHz Intel Core i7 processor), about 8 times faster on average than ReTrOS-Switch. The computations can be parallelised, which improves processing times particularly for large number of protein reporters or genes, and the current ReTrOS implementation provide the option to use workers of the parallel pool of the local machine. Whilst both methods require informative prior distributions of *δ*_*m*_ and *δ*_*p*_, the mRNA and protein degradation rates, ReTrOS-Switch samples the parameter distributions through the Metropolis-Hastings acceptance scheme and provides an updated posterior estimate of the distributions, whereas ReTrOS-Smooth draws directly from the given distributions only. Finally, in addition to the continuous estimate of transcription activity generated by both algorithms, ReTrOS-Switch also generates distributions over the timing and type of changes in transcriptional activity and therefore it is more appropriate when switching events are of interest.

## Results

The algorithms used in the ReTrOS toolbox have previously been applied to a number of different mRNA and protein reporter data sets, in addition to those in [1]. The study in [2] used the ReTrOS-Smooth to investigate the transcriptional dynamics of two protein-reporter systems, firefly luciferase (luc) and destabilized enhanced green fluorescent protein (EGFP), under identical promoter control in mammalian cells. The study in [3] used ReTrOS-Switch to explore correlations between discrete transcriptional ‘switch events’ and promoter structure and also obtained updated degradation rate estimates for 200 genes displaying a circadian rhythm in *Arabidopsis thaliana* leaf samples. Here we also present a new case study applying ReTrOS to recently published mRNA and protein-reporter time series of a selection of central *Arabidopsis thaliana* circadian clock-related genes. Circadian clocks and rhythms are present in most living organisms and provide a regulatory method for many important processes. A common feature of circadian clocks is that they consist of both transcriptional and translational components. We use the ReTrOS software to explore the oscillatory nature of the mRNA expression and protein reporter time series data from the model plant *Arabidopsis thaliana*.

**Fig. 6:**
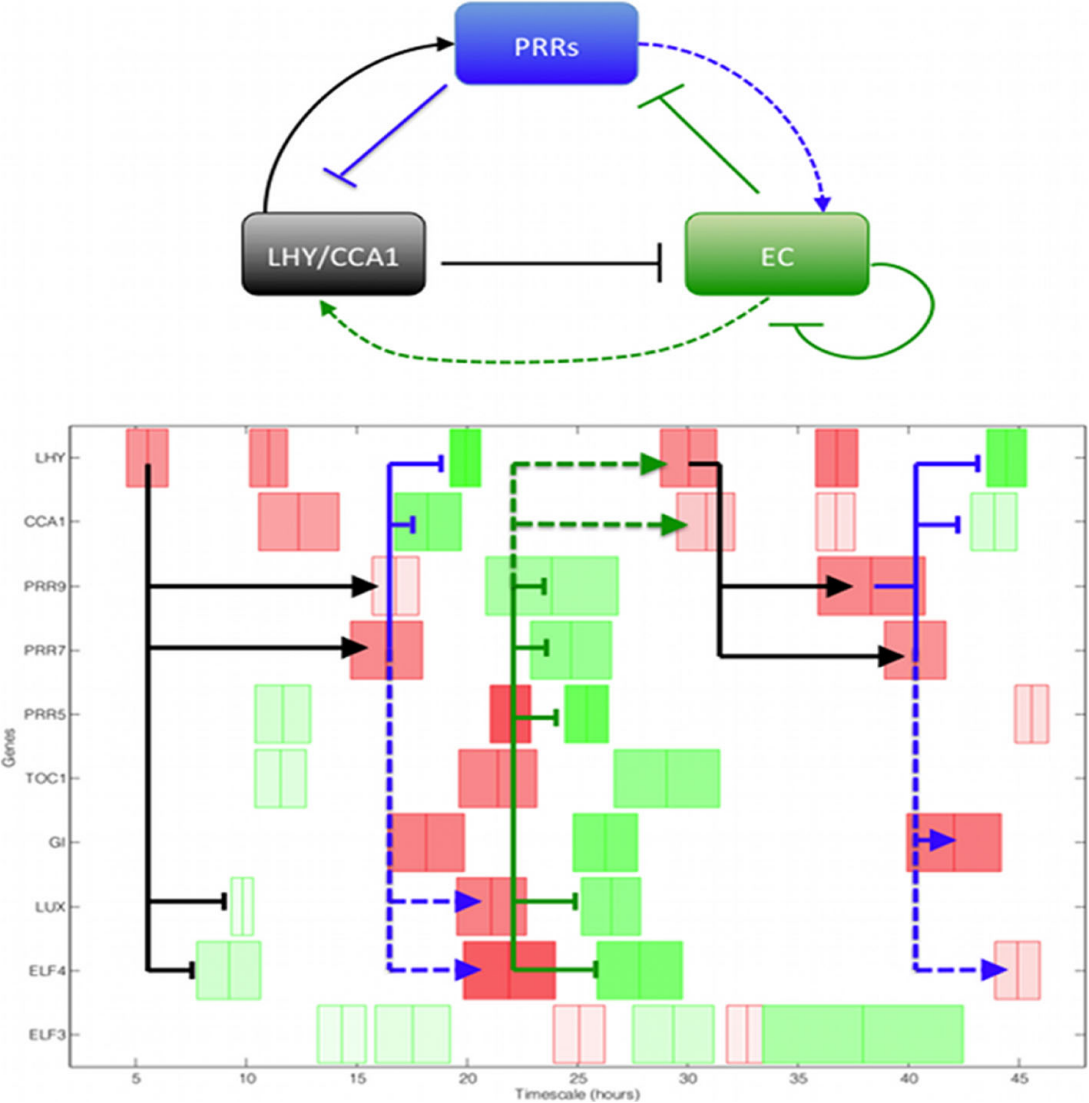
Arabidopsis clock repressilator circuit model and estimated transcriptional switch times. The *top panel* shows the core Arabidopsis clock repressilator circuit model from Pokhilko et al. [11], showing the 3 gene groups: LHY/CCA1 (*black*), PRRs (*blue*), EC (*dark green*). Direct regulatory interactions between the groups are shown by the *solid lines*, while the indirect interactions caused by the double-negative interactions are shown by *dotted lines*. The *bottom panel* shows the switch times and switch types heatmap estimated using the ReTrOS-Switch method for the selected 10 clock genes. The data used here is from microarray time-series for the clock genes in wild-type Arabidopsis plants (PRESTA project) [12]. Switches increasing the transcription rate are shown in *red*, decreasing transcription switches are shown in *green*. *Darker colours* represent more frequently sampled switch times. The direct interactions from the repressilator circuit model are shown by the *black*, *blue* and *dark green solid lines* and the indirect interactions are shown in *dashed lines* in the same colours. The oscillatory nature of the genes’ transcription is clearly identified in most of the genes and the putative interactions from the repressilator can also be determined in many cases. The default switch parameter values in the parameter setting GUI are used, with the prior mRNA degradation rate set by choosing *Arabidopsis*

**Identifying temporal events in the** ***Arabidopsis thaliana*** **circadian ‘repressilator’ circuit** Using the simplified core ‘repressilator’ model (shown in Fig. 6 top) introduced by Pokhilko et al. [11], we aim to identify the direct and indirect regulatory interactions caused by the double-negative feedback in terms of transcriptional switch timing. We selected 10 core clock genes (Table 1) including several from each broad gene group, LHY/CCA1, PRRs and Evening Complex (EC), and analysed mRNA microarray data from the 2-day wild-type time series dataset [12] using ReTrOS-Switch (shown in Fig. 6). The temporal switch distributions (shown at ±1.96*σ* with darker colours representing more frequently sampled switches) clearly show the circadian nature of the transcriptional profiles: for example LHY has three transcriptional switches, at approximately 6, 10 and 20 h, during the first 24-h observation period, followed by three switches of the same types at approximately the same times during the second 24-h period. However, not all of the analysed profiles displayed identifiable expression dynamics (specifically ELF3) and as such a clear transcriptional switch profile could not be obtained. The putative interactions of the repressilator model can be visually identified in many cases: for example the regulatory interactions by the LHY/CCA1 group (shown in black) can be identified with positive regulation of several PRR genes (increasing transcription switch event to increasing transcriptional switch event) and negative regulation of several EC genes (increasing transcriptional switch event to decreasing transcriptional switch event). The temporal flow of transcriptional regulation in waves can also be identified, with the LHY/CCA interactions followed by the PRR interactions which are followed by the EC interactions.

**Table 1:** Selected *Arabidopsis thaliana* clock genes

Gene	Group	Locus
LHY	LHY/CCA1	AT1G01060
CCA1	LHY/CCA1	AT2G46830
PRR9	PRRs	AT2G46790
PRR7	PRRs	AT5G02810
PRR5 (NI)	PRRs	AT5G24470
TOC1	-	AT5G61380
GI	-	AT1G22770
LUX	EC	AT3G46640
ELF4	EC	AT2G40080
ELF3	EC	AT2G25930

**Fig. 7:**
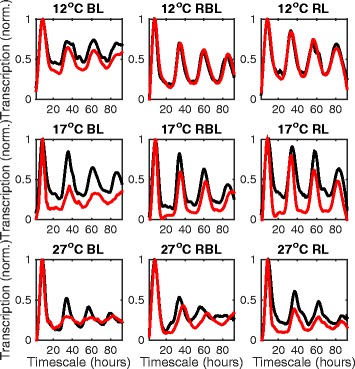
Back-calculated transcription profiles of the wild-type and cry1 cry2 double-mutant CCR2 reporter lines under different temperature and light conditions. Each panel shows the back-calculated wild-type CCR2 (*black line*) and the double-mutant cry1 cry2 CCR2 (*red line*) transcription profiles at 12 (*top row*), 17 (*middle row*) and 27 °C (*bottom row*) with *blue* (BL, *left column*), mixed *red* and *blue* (RBL, *middle column*) and *red light* (RL, *right column*) conditions. The rhythmic activity is observable in all profiles, but with strong damping at 17 and 27 °C across most light conditions. A lengthening of oscillatory period is particularly observable in the mutant line at 27 °C under RBL conditions. Degradation rates (mean, std. dev.) of mRNA and protein are respectively (2.8, 0.63) and (0.098, 0.0058) at 12 °C, (2.3, 0.46) and (0.13, 0.010) at 17 °C, and (1.2, 0.11) and (0.19, 0.020) at 27 °C. For other parameter values, see the *middle panel* of Fig. 5

**Effects of light and temperature conditions on circadian marker genes** We analysed protein luciferase reporter data from a 4-day time series dataset of the circadian-controlled CCR2 and CAB2 promoters in a range of wild-type and mutant lines, temperature conditions and light regimes [13]. Applying the ReTrOS-Smooth algorithm to the detrended data and combining replicate observations from the same lines yields clear rhythmic back-calculated transcriptional profiles under most experimental conditions. Figure 7 shows the back-calculated transcription profiles for the wild-type CCR2(3) line (shown by the black line) and the *cry1 cry* CCR2(3) double-mutant line (shown by the red line at temperatures of 12, 17 and 27 °C and blue (BL), red (RL) and mixed red-blue (RBL) light conditions. We observe similar behaviours to those identified within the original study, such as an increased period in the *cry1 cry2* CCR2 mutant line at 27 °C under RBL conditions, however, the back-calculated transcription profile of the *cry1 cry2* CCR2 still shows damped rhythmic dynamics. As the back-calculation model takes into account the degradation processes of the luceriferase reporter, finer-scale structures in the time series are able to be extracted which may allow, for instance, increased accuracy in periodicity inference when using methods robust to asymmetric oscillations such as spectrum resampling [14].

## Conclusions

Analysis of large-scale and high-throughput data is becoming an increasingly common task for many researchers. We provide an easy-to-use toolbox for the analysis of mRNA or protein-reporter time series data, that generates a fine-scale profile of transcriptional activity by removing the effects of degradation processes from the observed data (Additional file 1). The ReTrOS toolbox has been applied to a variety of datasets from a range of different technologies and platforms. ReTrOS can be easily incorporated into a computational or analysis pipeline as either, a data preprocessing step for rapidly obtaining high-resolution back-calculated transcriptional profiles which are then used in other analysis steps, or directly as an analysis tool extracting distributions of transcriptional ‘switching’ activity and degradation rate estimates. There are a number of possible extensions to the analysis including parallelisation of the ReTrOS-Smooth bootstrap procedure and parallel MCMC sampling in the ReTrOS-Switch algorithm, the application of further analysis methods such as clustering on the model outputs and the use of Bayesian hierarchical modelling for multiple time series [3]. The methodology for a stochastic transcriptional switch model is considerably more challenging and has recently been developed in [5] and [6].

## Availability and requirements

The ReTrOS toolbox, user manual and example data are freely available from http://www2.warwick.ac.uk/fac/sci/systemsbiology/research/software/ and https://github.com/giorgosminas/ReTrOS under the GNU General Public License https://www.gnu.org/licenses/gpl-3.0.en.html. The toolbox runs in MATLAB®; that can operate in Windows, Mac and Linux (see https://uk.mathworks.com/support/sysreq.html).

## Additional file

Additional file 1 Toolbox and data. The MATLAB code for running ReTrOS toolbox. This also includes the data used in the illustrative examples and the user manual that provides the instructions on how to run the toolbox. (ZIP 52838 kb)

## Abbreviations

BL: Blue light
CAB2: Chlorophyll A/B-binding protein 2
CCA1: Circadian clock associated 1
CCR2: Cold, circadian rhythm, and RNA binding 2
EC: Evening complex
EGFP: Destabilized enhanced green fluorescent protein
GUI: Graphical user interface
LHY: Late elongated Hypocotyl
luc: firefly luciferase
MCMC: Markov Chain Monte Carlo
mRNA: Messenger Ribonucleic acid
ODE: Ordinary differential equation
PRR: Pseudo-response regulator
RBL: Mixed red-blue light
ReTrOS: Reconstructing transcription open software
RL: Red light
