# Supplementary material for: ReTrOS: a MATLAB toolbox for reconstructing transcriptional activity from gene and protein expression data
Source: BMC Bioinformatics. 2017 Jun 26;18:316. doi: 10.1186/s12859-017-1695-8 (PMC5485715; doi:10.1186/s12859-017-1695-8)
Supplement: Additional file 1 — Toolbox and data. The MATLAB code for running ReTrOS toolbox. This also includes the data used in the illustrative examples and the user manual that provides the instructions on how to run the toolbox. (ZIP 52838 kb) [file 12859_2017_1695_MOESM1_ESM.zip › ReTrOS-master/ReTrOSv5.4/output/clock_genes_mRNA/clock_genes_mRNA_switch.pdf]

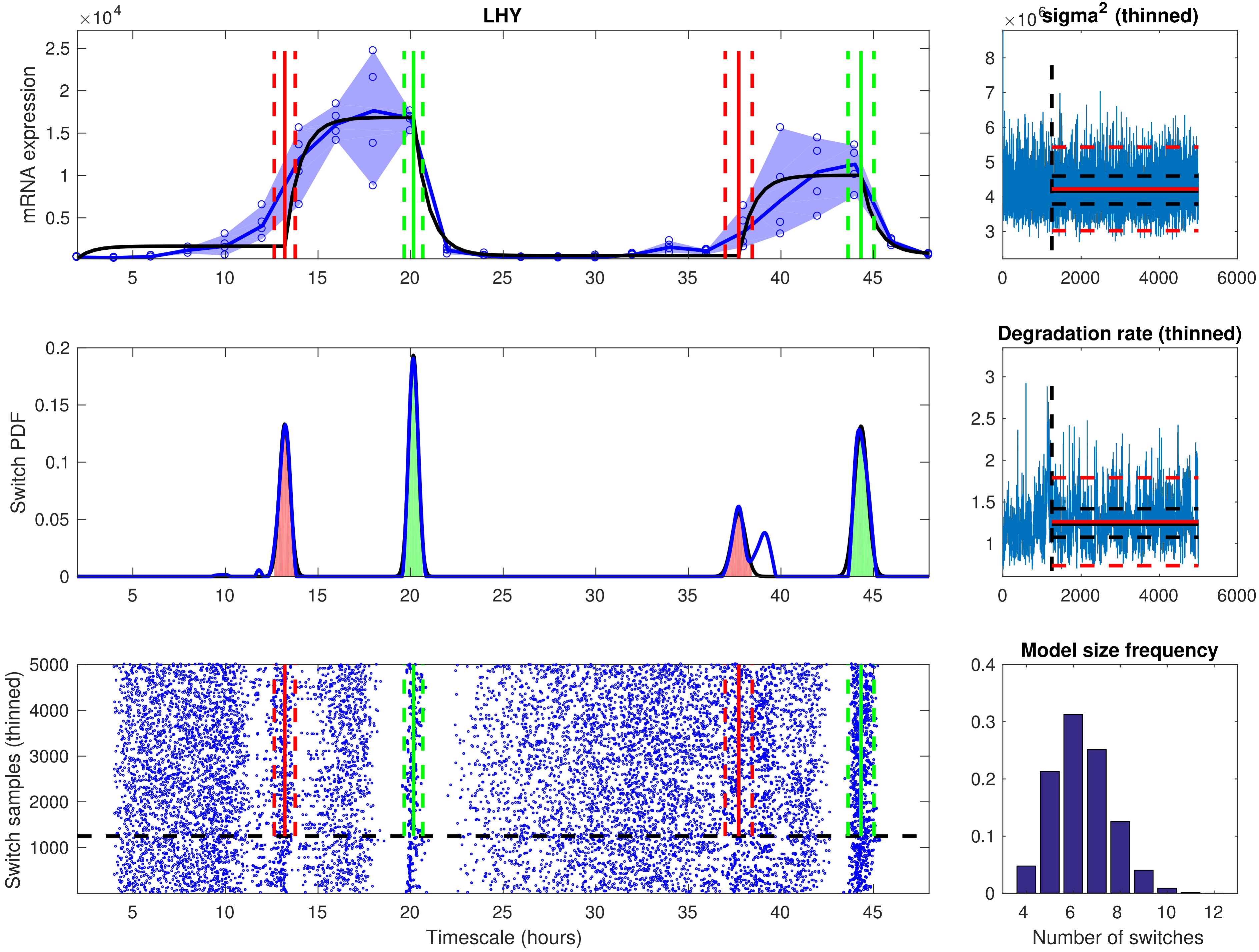

LHY
$\times 10^4$
mRNA expression
sigma^2 (thinned)
$\times 10^6$
Degradation rate (thinned)
Switch PDF
Switch samples (thinned)
Timescale (hours)
Model size frequency
Number of switches

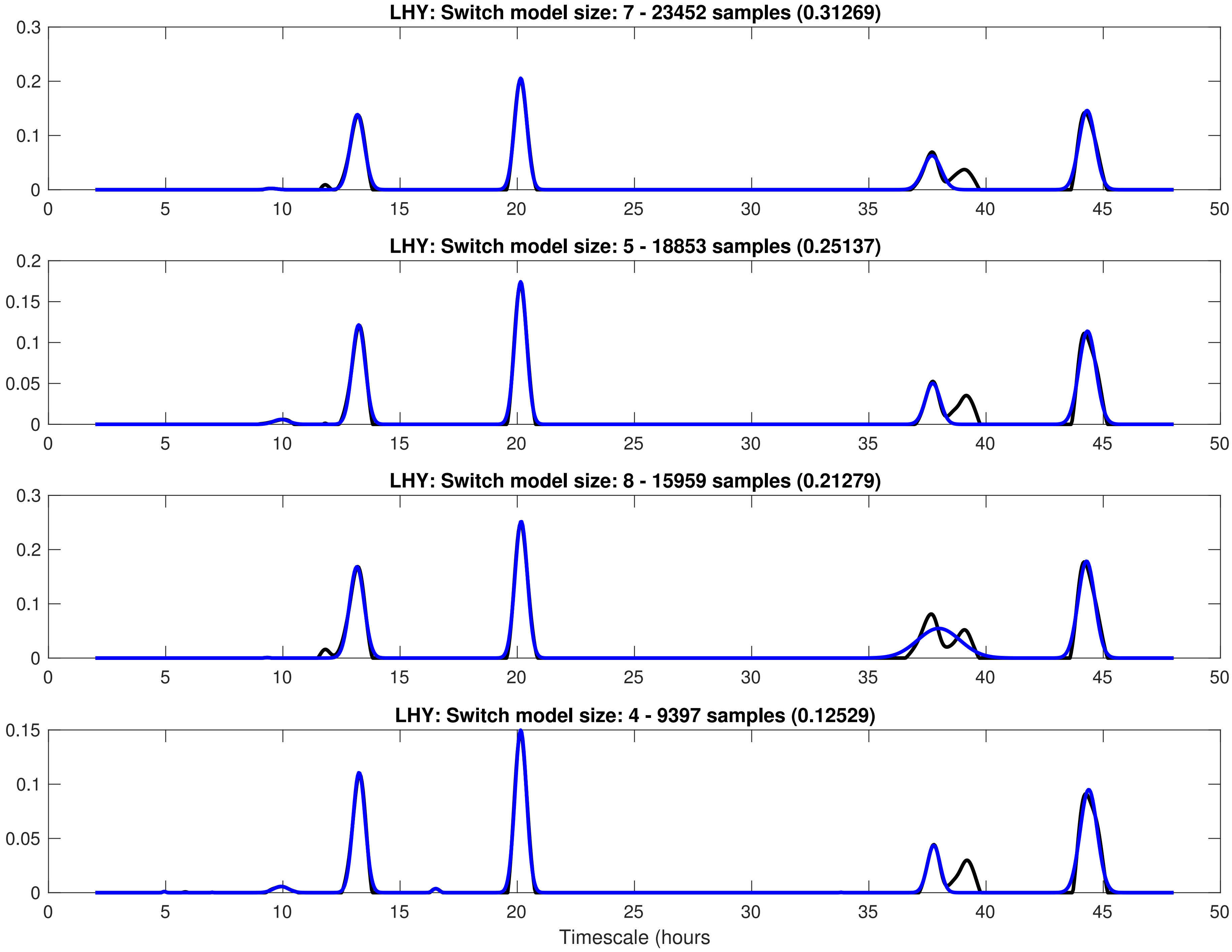

LHY: Switch model size: 7 - 23452 samples (0.31269)
LHY: Switch model size: 5 - 18853 samples (0.25137)
LHY: Switch model size: 8 - 15959 samples (0.21279)
LHY: Switch model size: 4 - 9397 samples (0.12529)
Timescale (hours

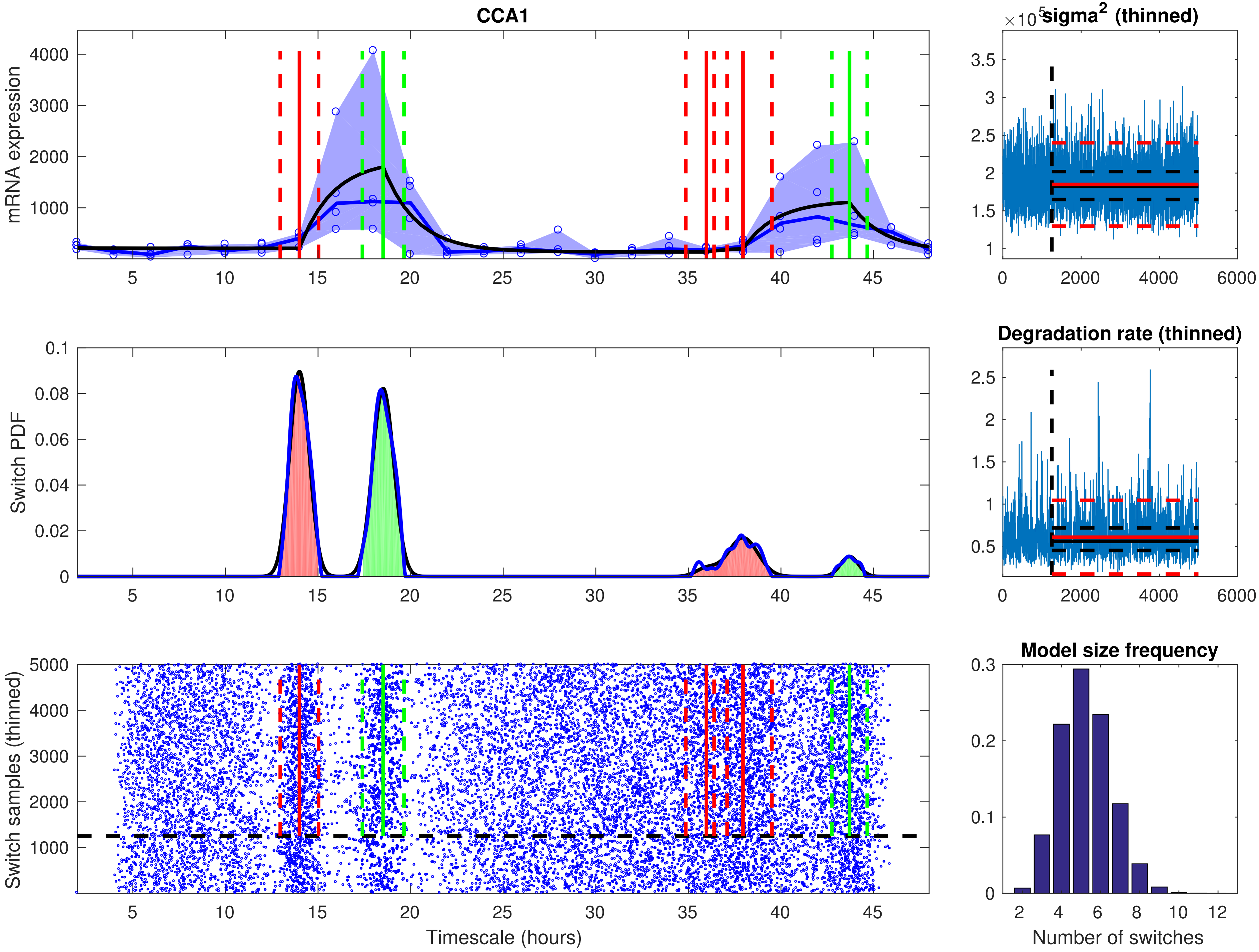
CCA1
mRNA expression
Switch PDF
Switch samples (thinned)
Timescale (hours)
sigma^2 (thinned)
$\times 10^5$
Degradation rate (thinned)
Model size frequency
Number of switches

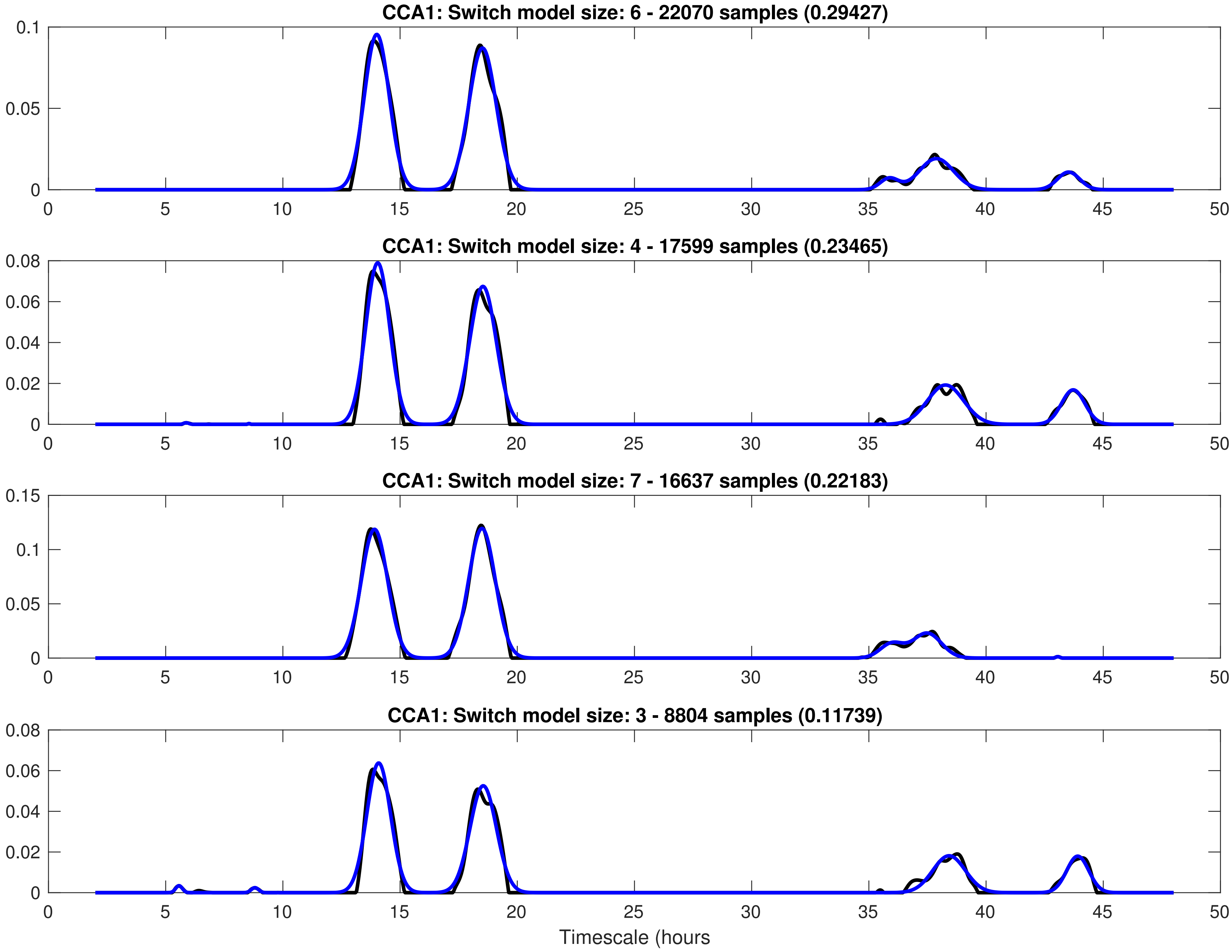

CCA1: Switch model size: 6 - 22070 samples (0.29427)
CCA1: Switch model size: 4 - 17599 samples (0.23465)
CCA1: Switch model size: 7 - 16637 samples (0.22183)
CCA1: Switch model size: 3 - 8804 samples (0.11739)
Timescale (hours

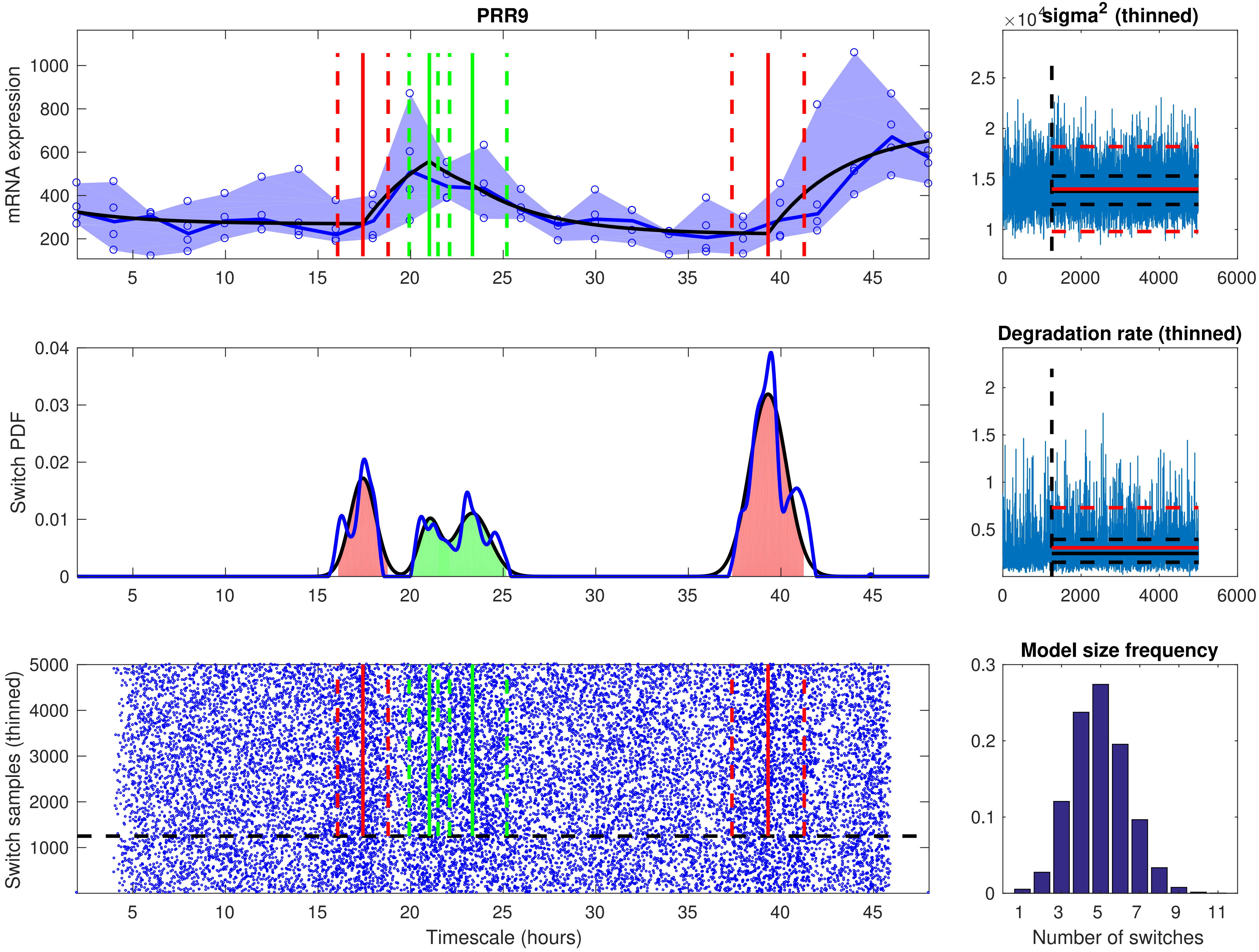
PRR9
mRNA expression
×10^4 sigma^2 (thinned)
Switch PDF
Degradation rate (thinned)
Switch samples (thinned)
Timescale (hours)
Model size frequency
Number of switches

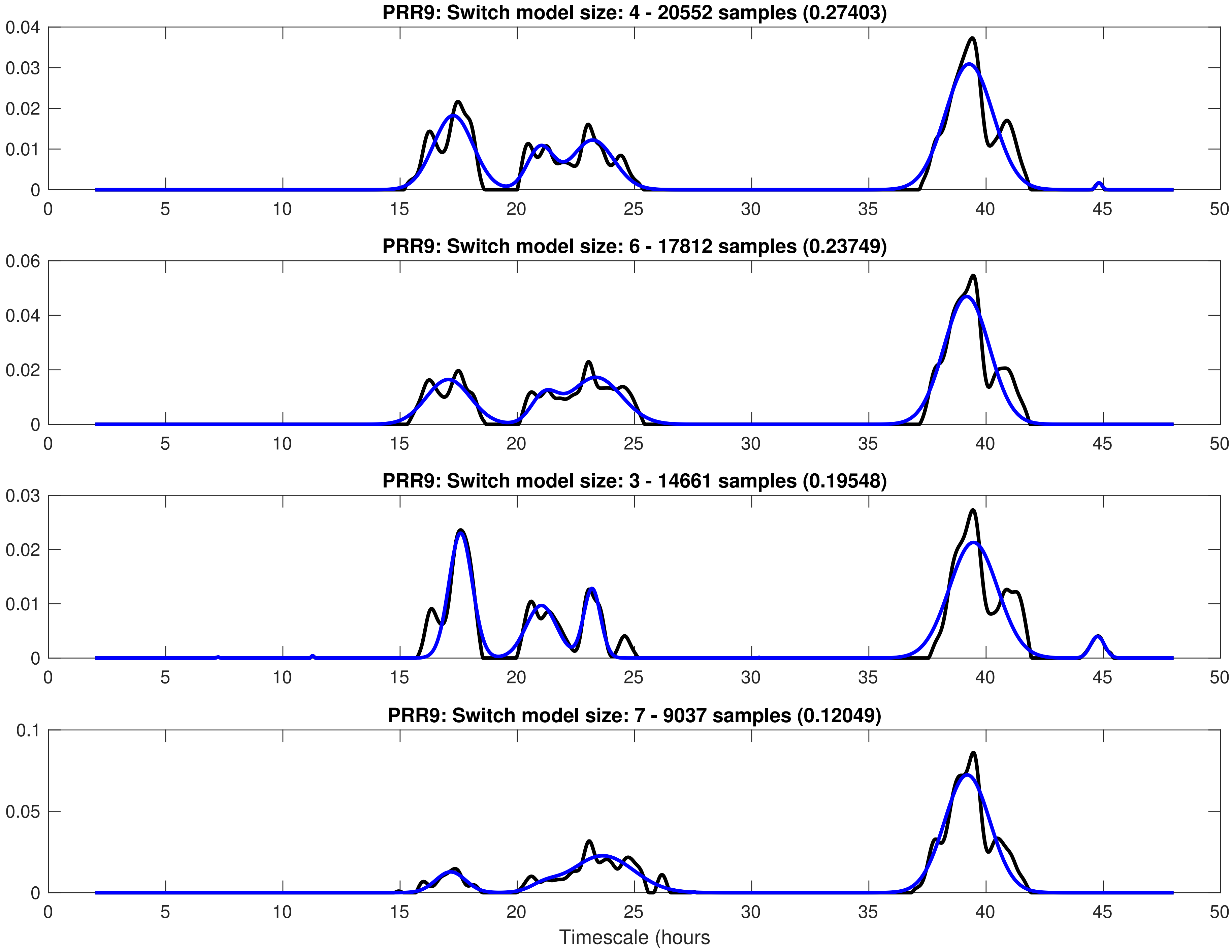

PRR9: Switch model size: 4 - 20552 samples (0.27403)
PRR9: Switch model size: 6 - 17812 samples (0.23749)
PRR9: Switch model size: 3 - 14661 samples (0.19548)
PRR9: Switch model size: 7 - 9037 samples (0.12049)
Timescale (hours

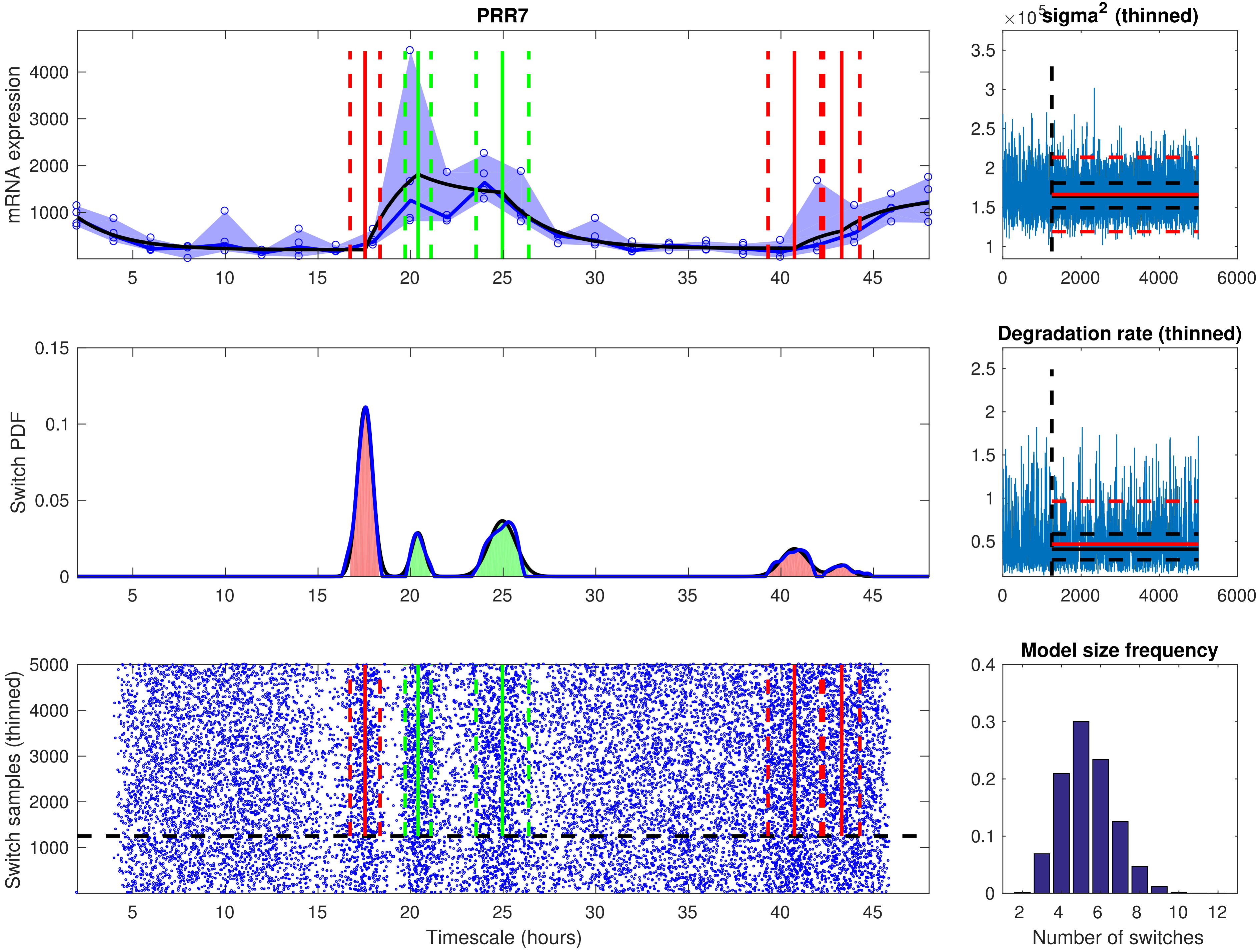

PRR7
mRNA expression
Switch PDF
Switch samples (thinned)
Timescale (hours)
×10^5 sigma^2 (thinned)
Degradation rate (thinned)
Model size frequency
Number of switches

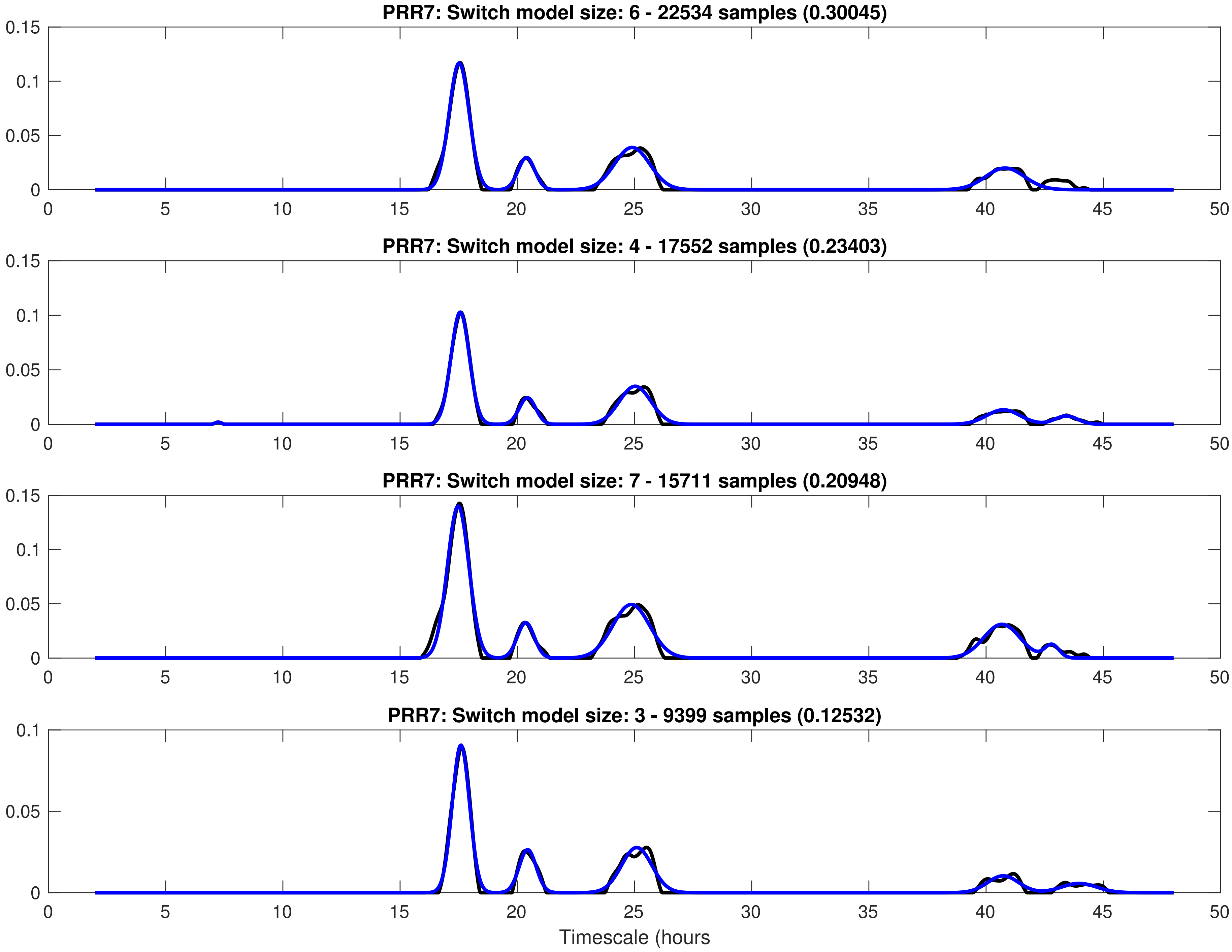
PRR7: Switch model size: 6 - 22534 samples (0.30045)
PRR7: Switch model size: 4 - 17552 samples (0.23403)
PRR7: Switch model size: 7 - 15711 samples (0.20948)
PRR7: Switch model size: 3 - 9399 samples (0.12532)
0.15
0.1
0.05
0
0
5
10
15
20
25
30
35
40
45
50
Timescale (hours

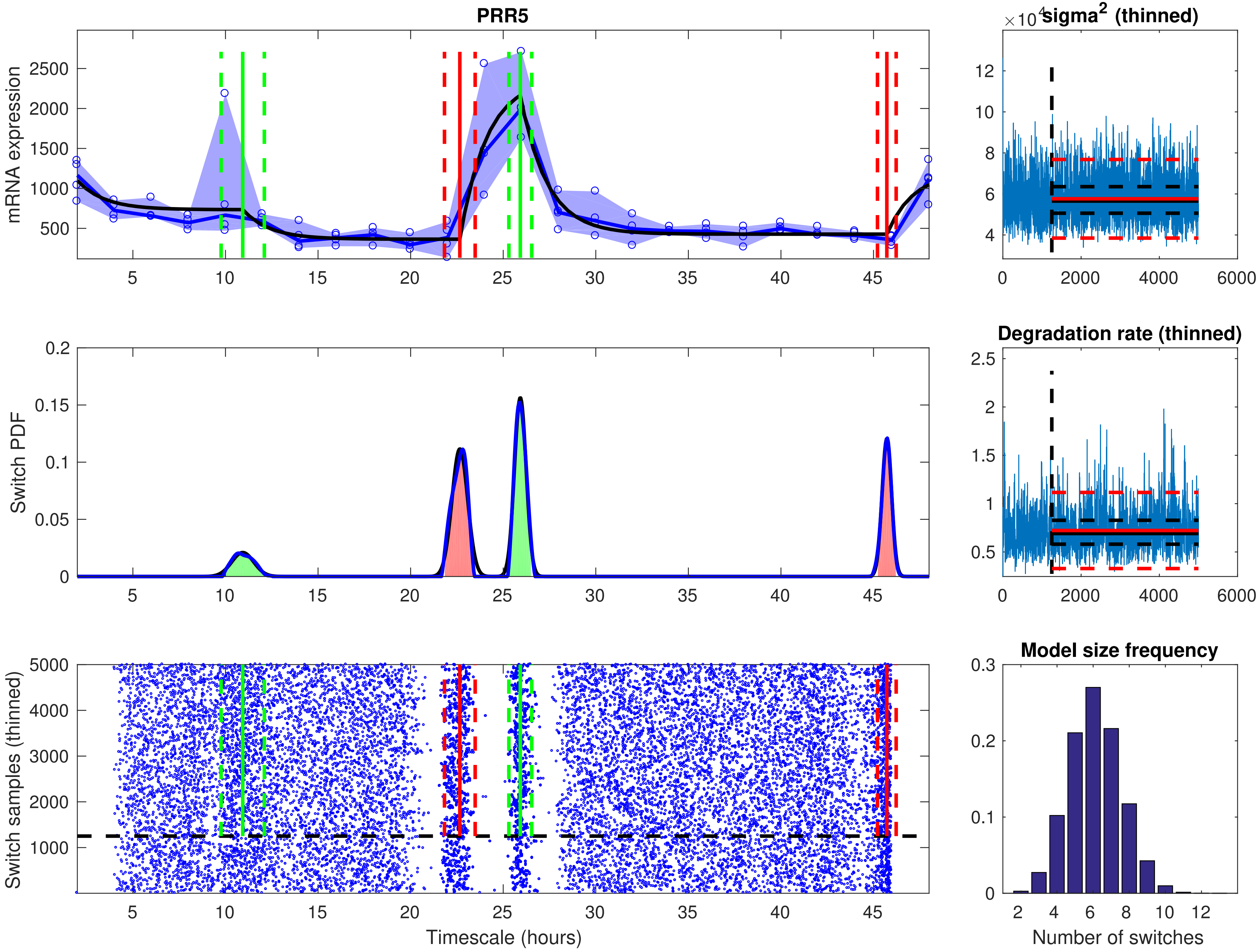
PRR5
mRNA expression
Switch PDF
Switch samples (thinned)
Timescale (hours)
$\times 10^4$ sigma^2 (thinned)
Degradation rate (thinned)
Model size frequency
Number of switches

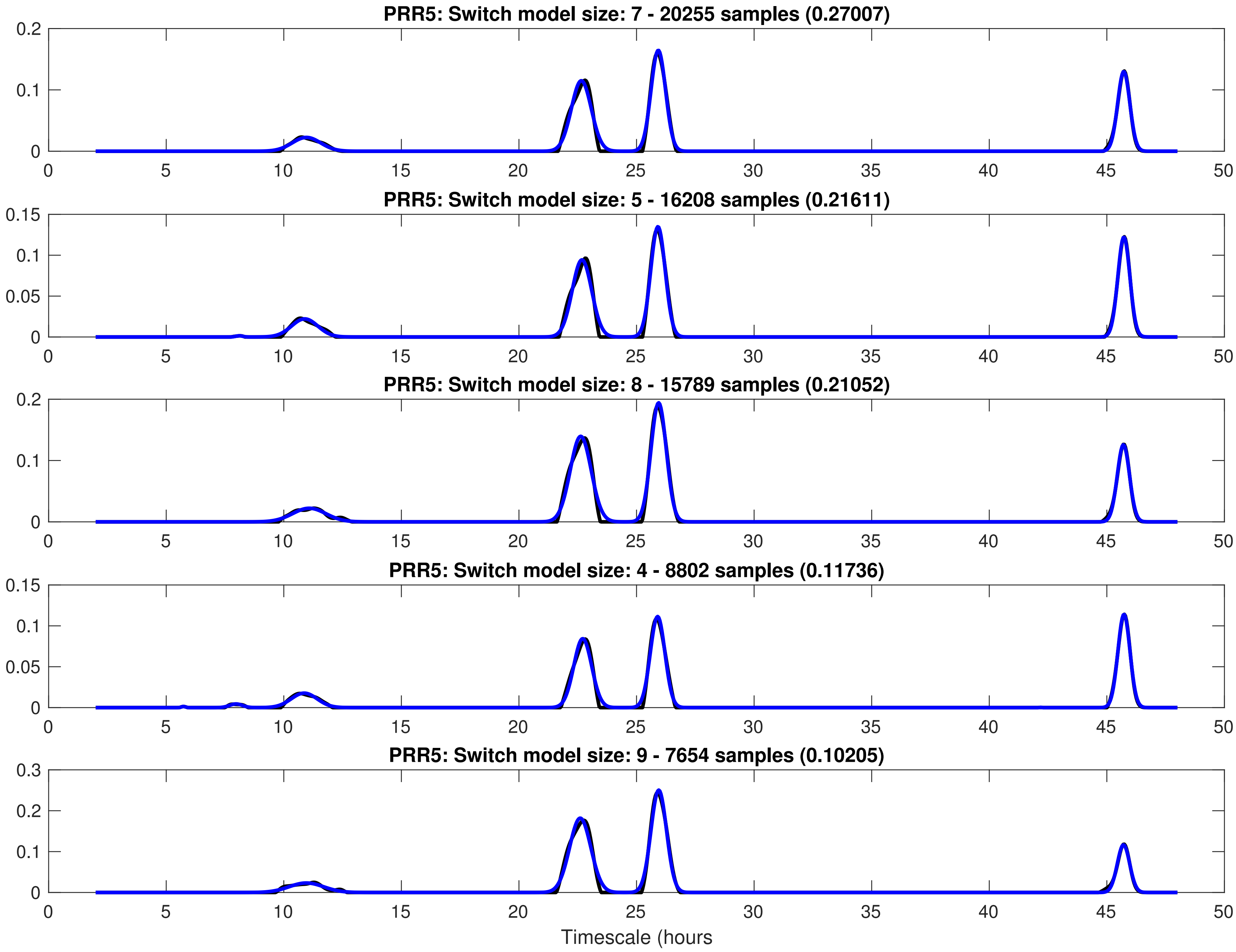

PRR5: Switch model size: 7 - 20255 samples (0.27007)
PRR5: Switch model size: 5 - 16208 samples (0.21611)
PRR5: Switch model size: 8 - 15789 samples (0.21052)
PRR5: Switch model size: 4 - 8802 samples (0.11736)
PRR5: Switch model size: 9 - 7654 samples (0.10205)
Timescale (hours

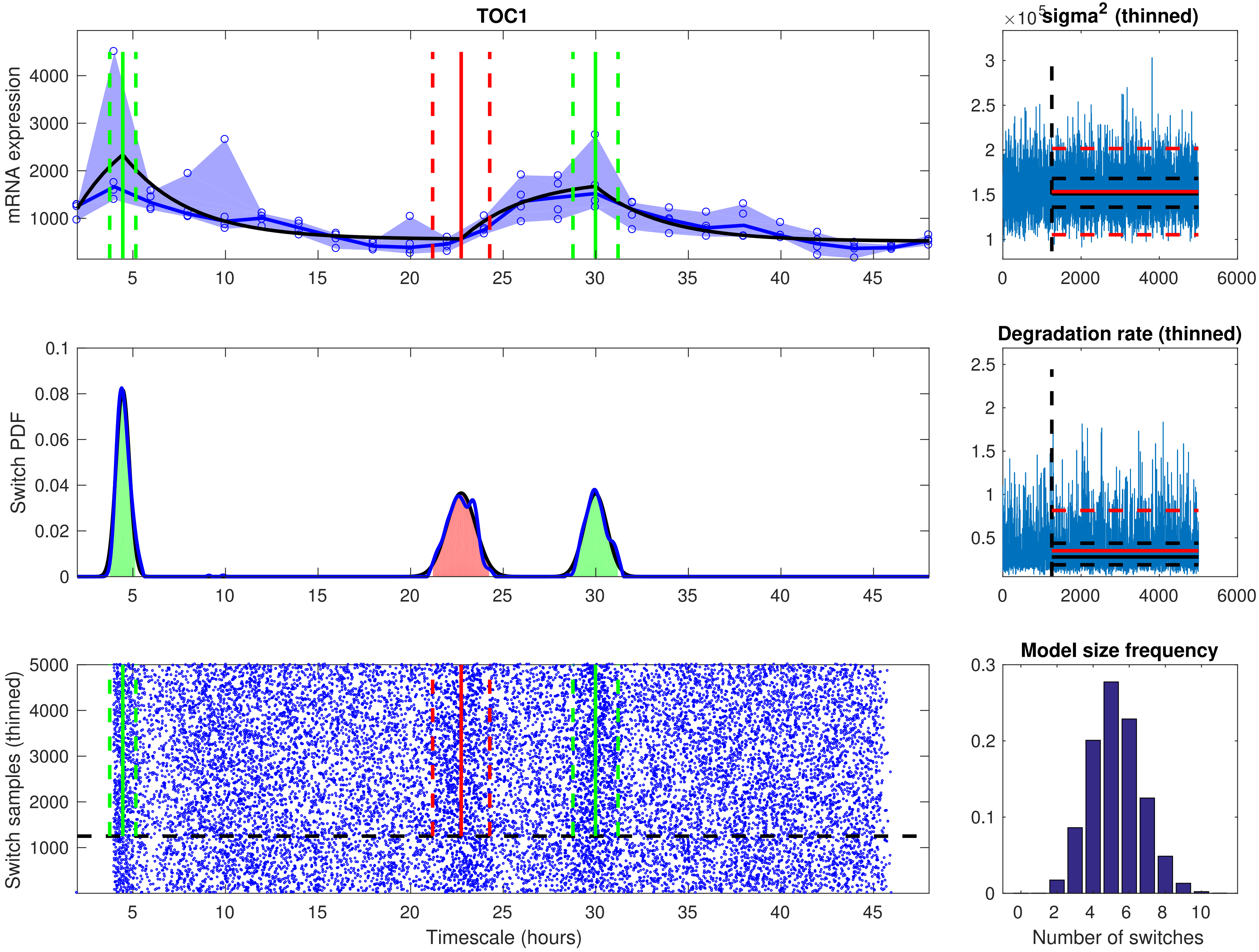

TOC1
mRNA expression
Switch PDF
Switch samples (thinned)
Timescale (hours)
×10^5 sigma^2 (thinned)
Degradation rate (thinned)
Model size frequency
Number of switches

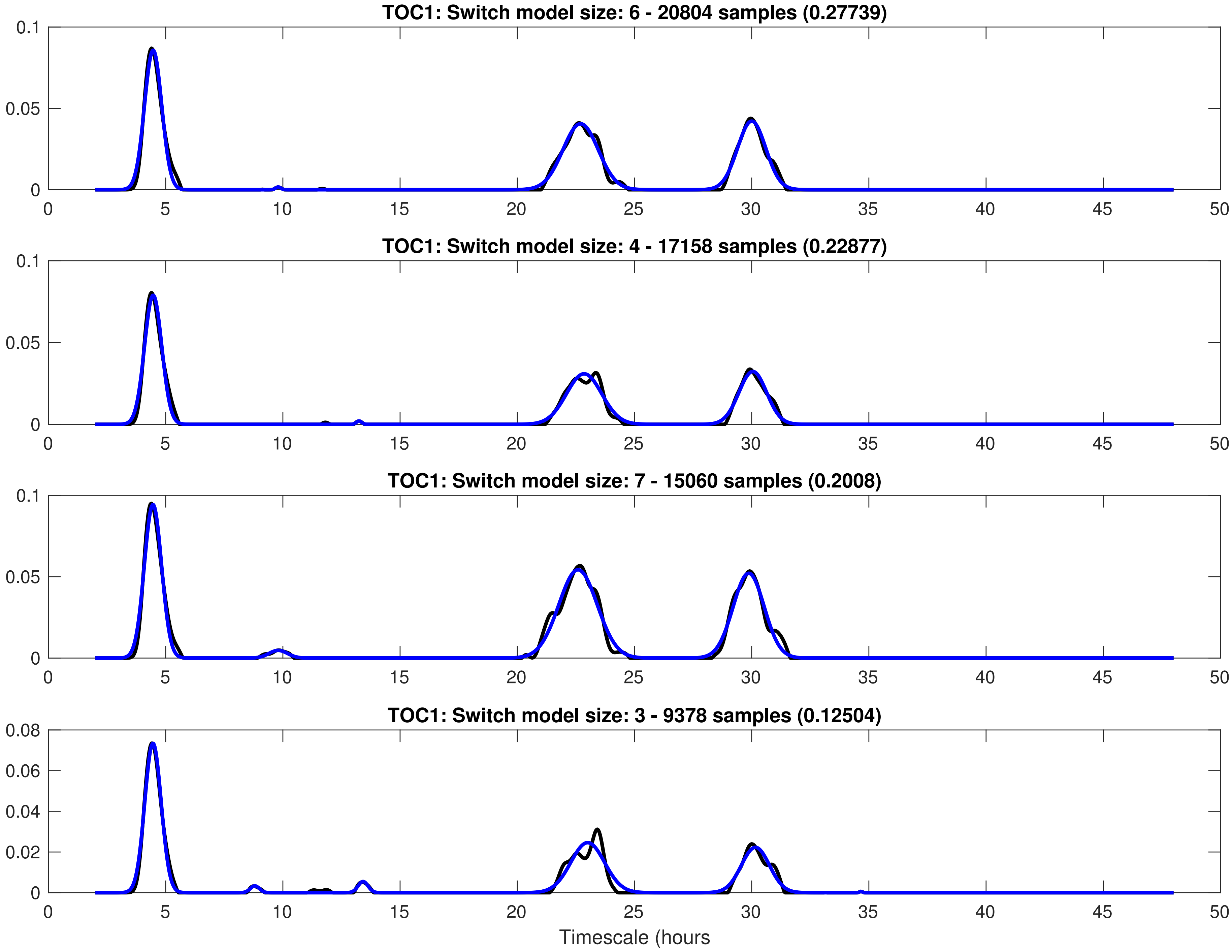
TOC1: Switch model size: 6 - 20804 samples (0.27739)
TOC1: Switch model size: 4 - 17158 samples (0.22877)
TOC1: Switch model size: 7 - 15060 samples (0.2008)
TOC1: Switch model size: 3 - 9378 samples (0.12504)
Timescale (hours

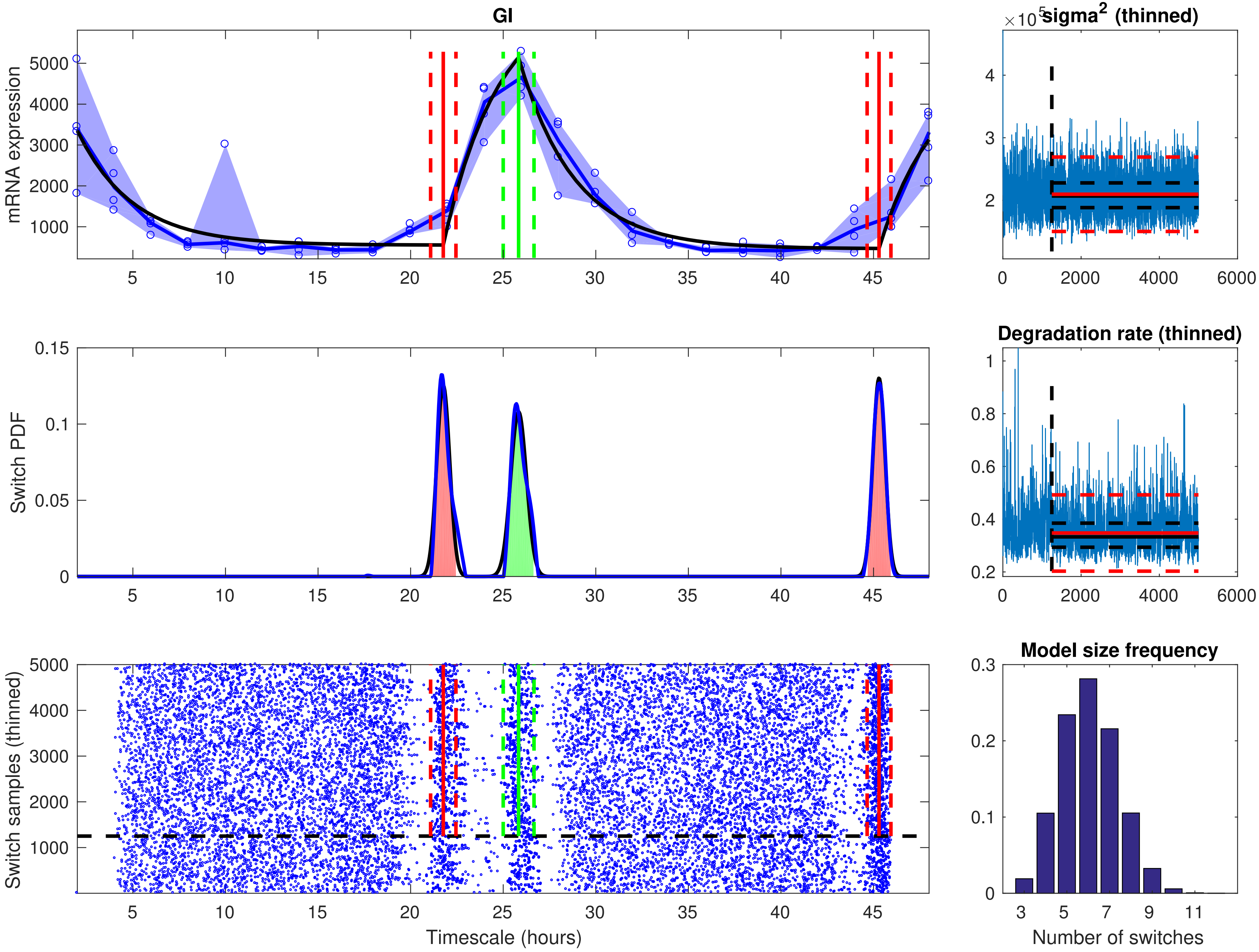
GI
mRNA expression
sigma^2 (thinned)
$\times 10^5$
Degradation rate (thinned)
Switch PDF
Switch samples (thinned)
Timescale (hours)
Model size frequency
Number of switches

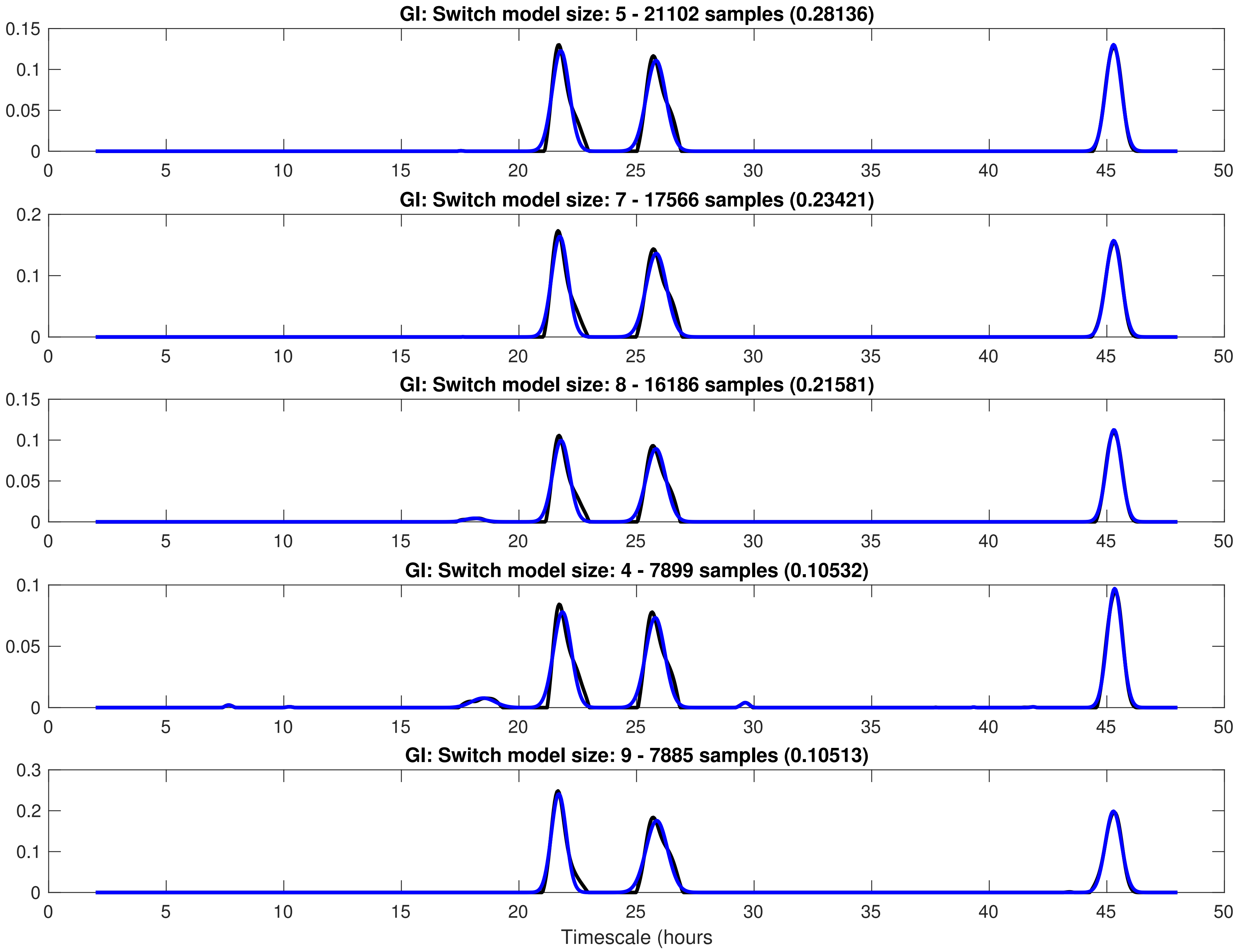

GI: Switch model size: 5 - 21102 samples (0.28136)
GI: Switch model size: 7 - 17566 samples (0.23421)
GI: Switch model size: 8 - 16186 samples (0.21581)
GI: Switch model size: 4 - 7899 samples (0.10532)
GI: Switch model size: 9 - 7885 samples (0.10513)
Timescale (hours

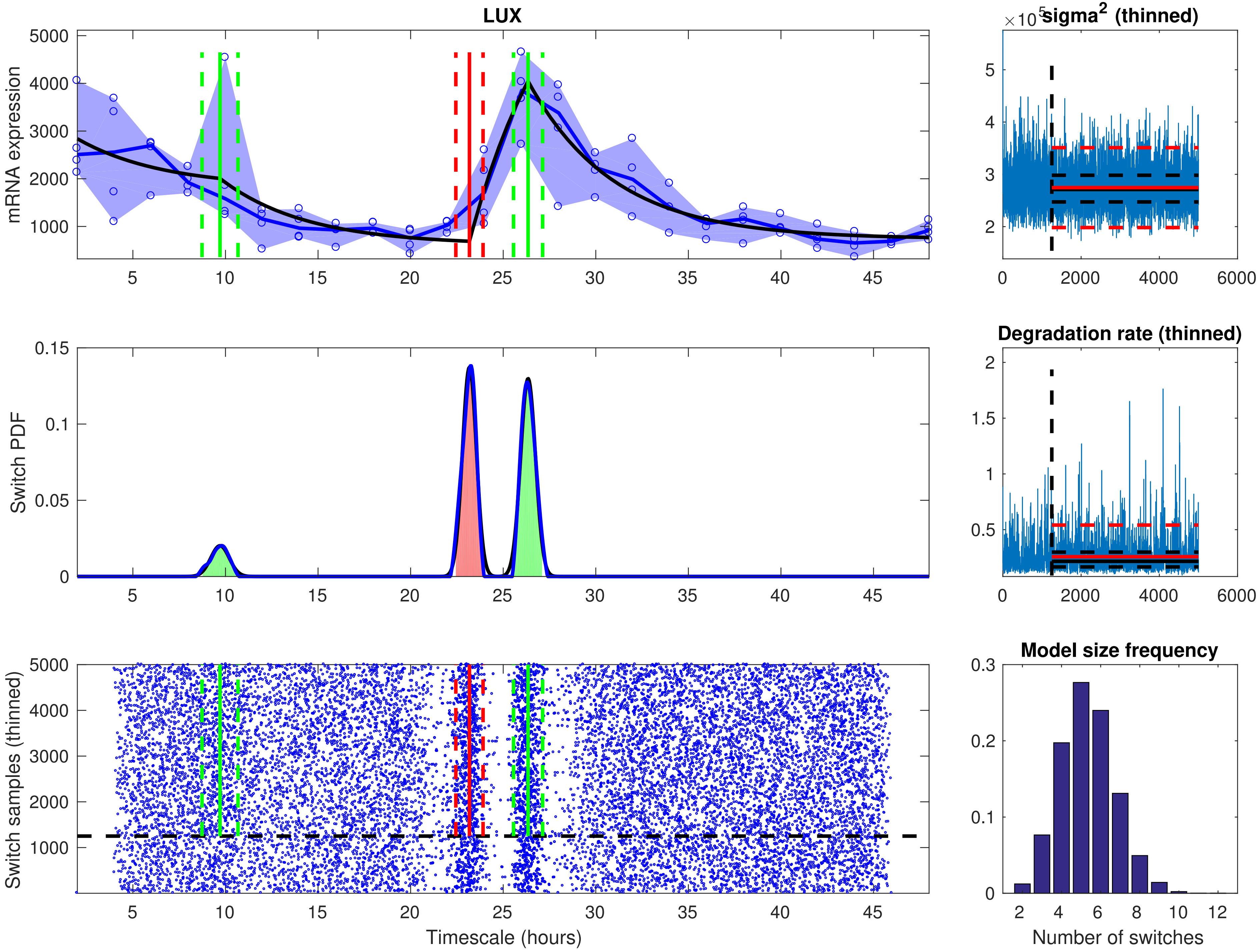

LUX
mRNA expression
Switch PDF
Switch samples (thinned)
Timescale (hours)
×10^5 sigma^2 (thinned)
Degradation rate (thinned)
Model size frequency
Number of switches

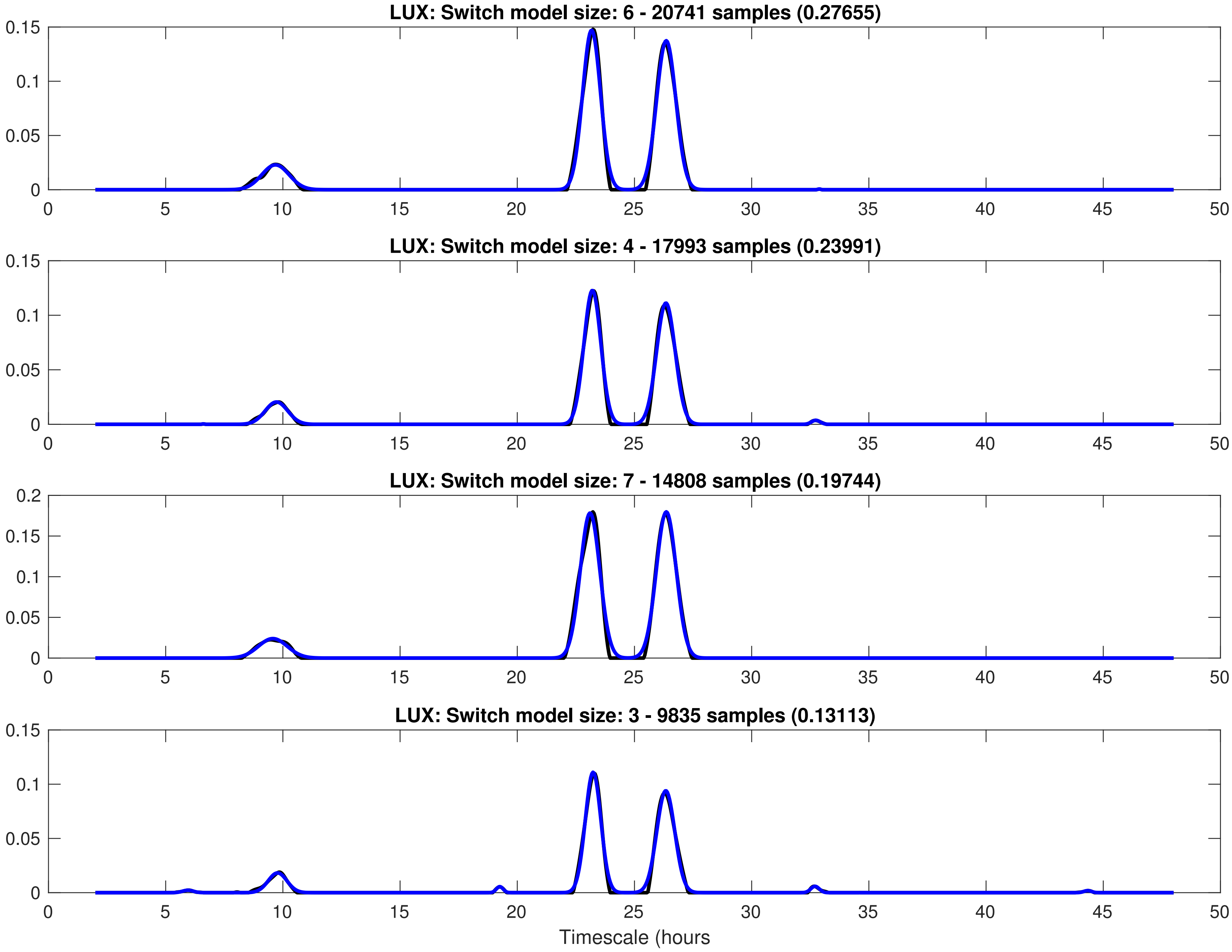
LUX: Switch model size: 6 - 20741 samples (0.27655)
LUX: Switch model size: 4 - 17993 samples (0.23991)
LUX: Switch model size: 7 - 14808 samples (0.19744)
LUX: Switch model size: 3 - 9835 samples (0.13113)
Timescale (hours

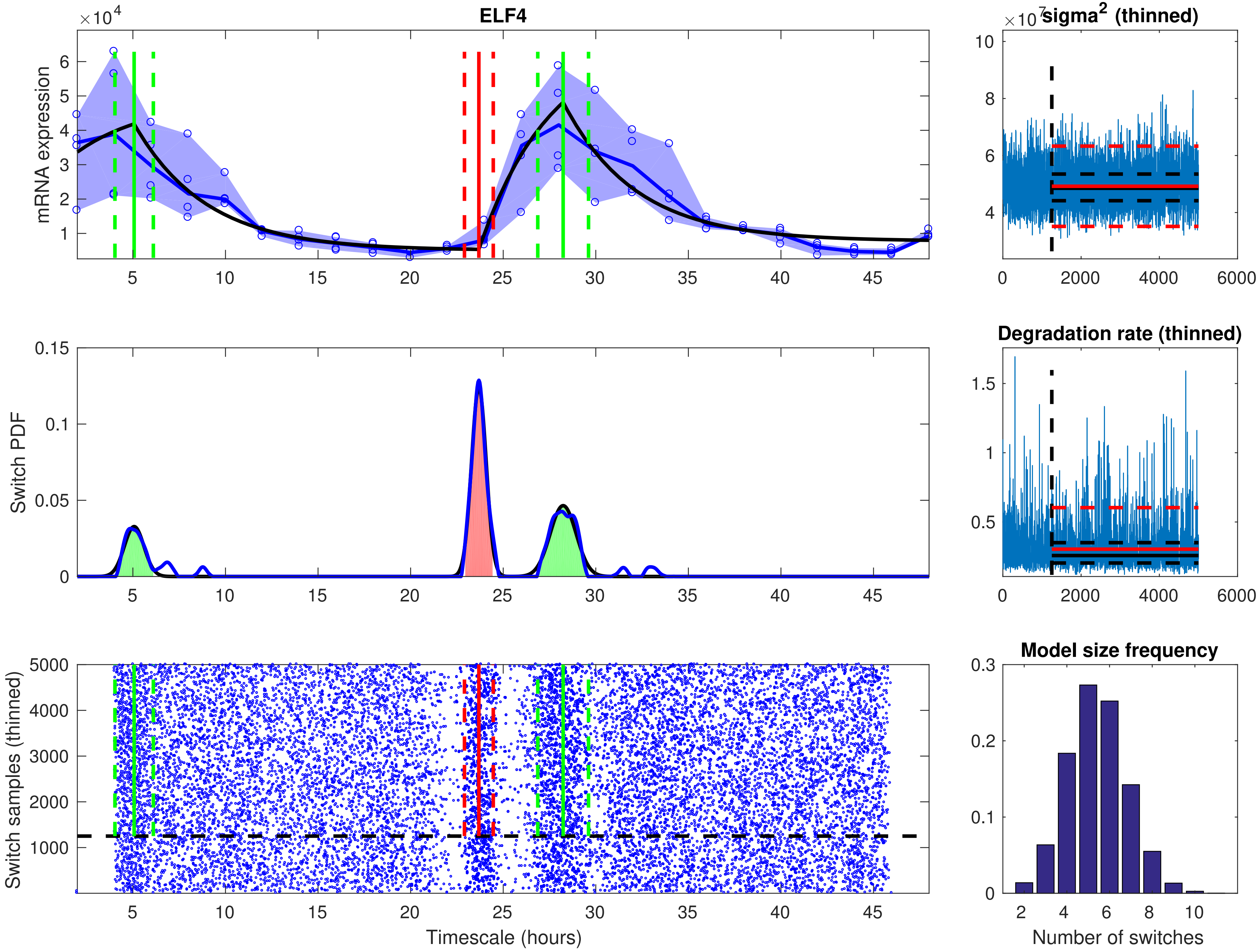

ELF4
mRNA expression
×10^4
sigma^2 (thinned)
×10^7
Switch PDF
Degradation rate (thinned)
Switch samples (thinned)
Timescale (hours)
Model size frequency
Number of switches

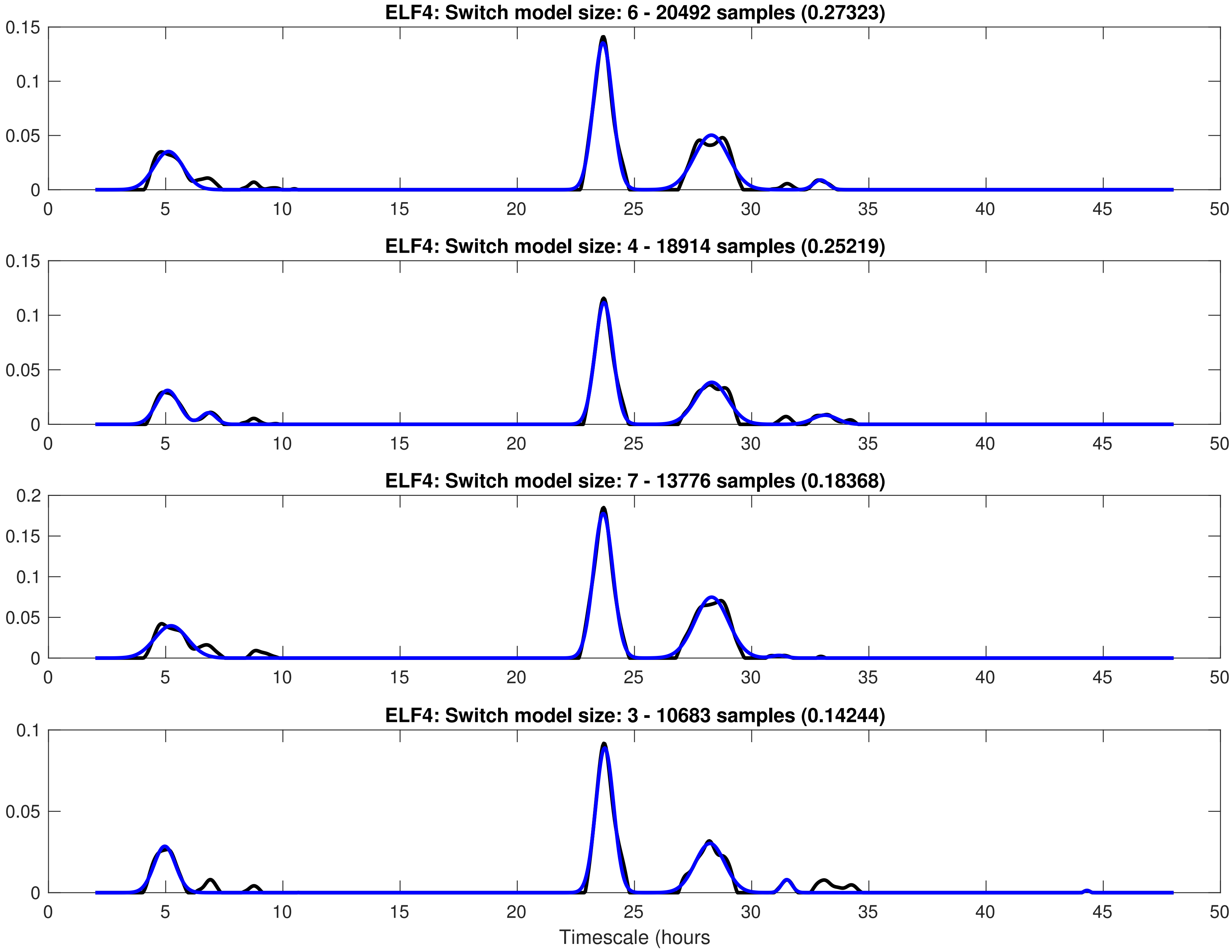
ELF4: Switch model size: 6 - 20492 samples (0.27323)
ELF4: Switch model size: 4 - 18914 samples (0.25219)
ELF4: Switch model size: 7 - 13776 samples (0.18368)
ELF4: Switch model size: 3 - 10683 samples (0.14244)
Timescale (hours

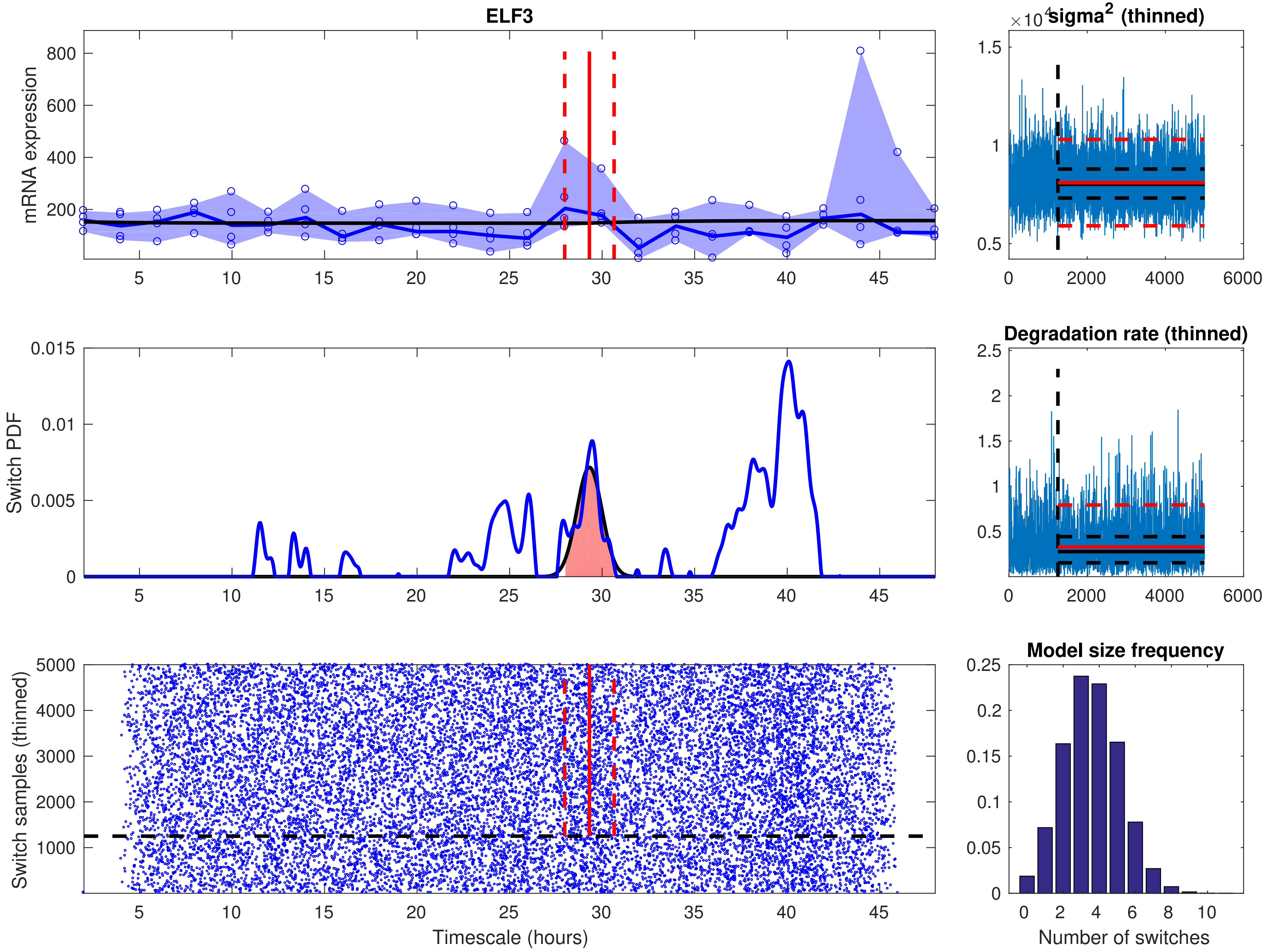

ELF3
mRNA expression
Switch PDF
Switch samples (thinned)
Timescale (hours)
×10^4 sigma^2 (thinned)
Degradation rate (thinned)
Model size frequency
Number of switches

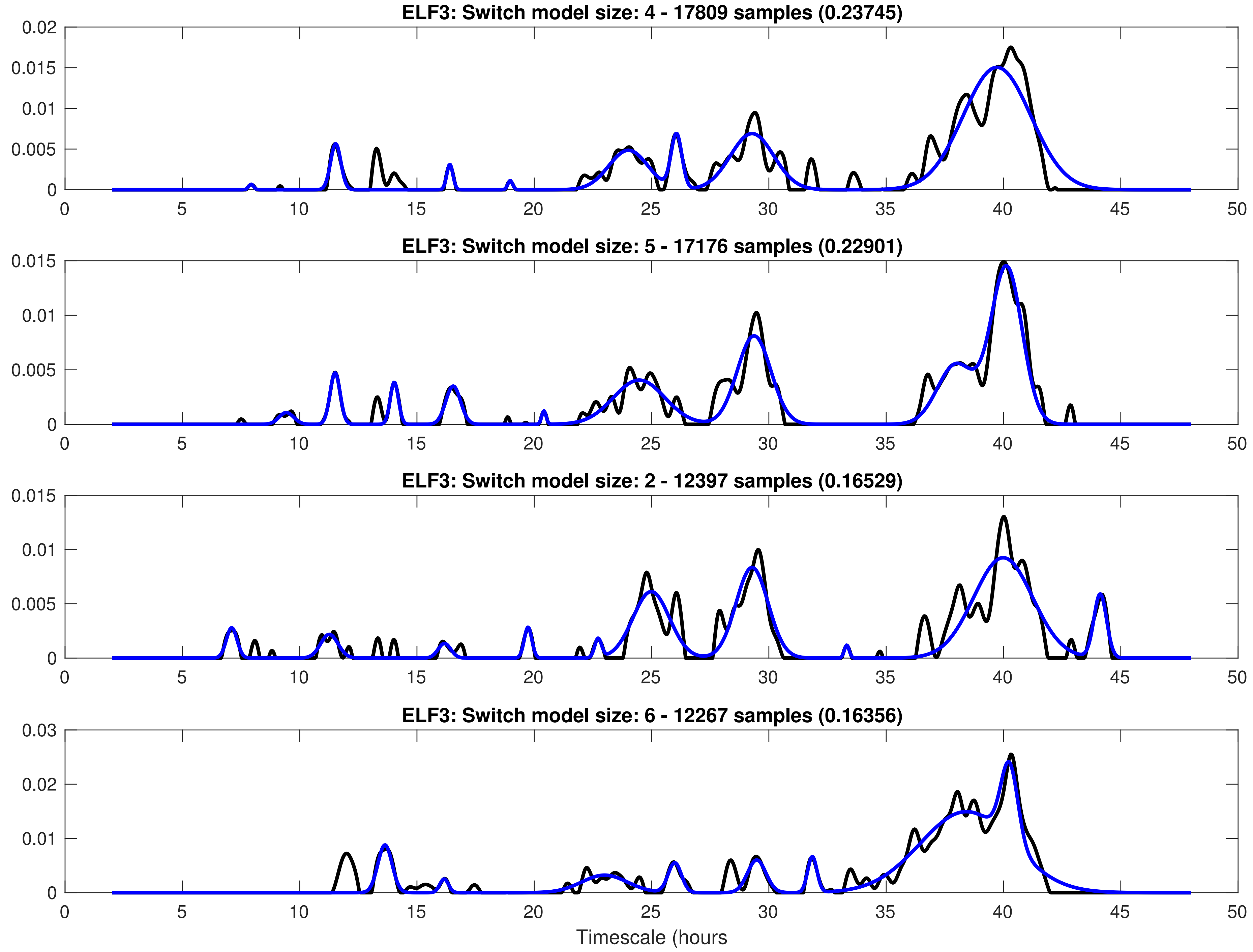

ELF3: Switch model size: 4 - 17809 samples (0.23745)
0.02
0.015
0.01
0.005
0
0
5
10
15
20
25
30
35
40
45
50
ELF3: Switch model size: 5 - 17176 samples (0.22901)
0.015
0.01
0.005
0
ELF3: Switch model size: 2 - 12397 samples (0.16529)
0.015
0.01
0.005
0
ELF3: Switch model size: 6 - 12267 samples (0.16356)
0.03
0.02
0.01
0
Timescale (hours

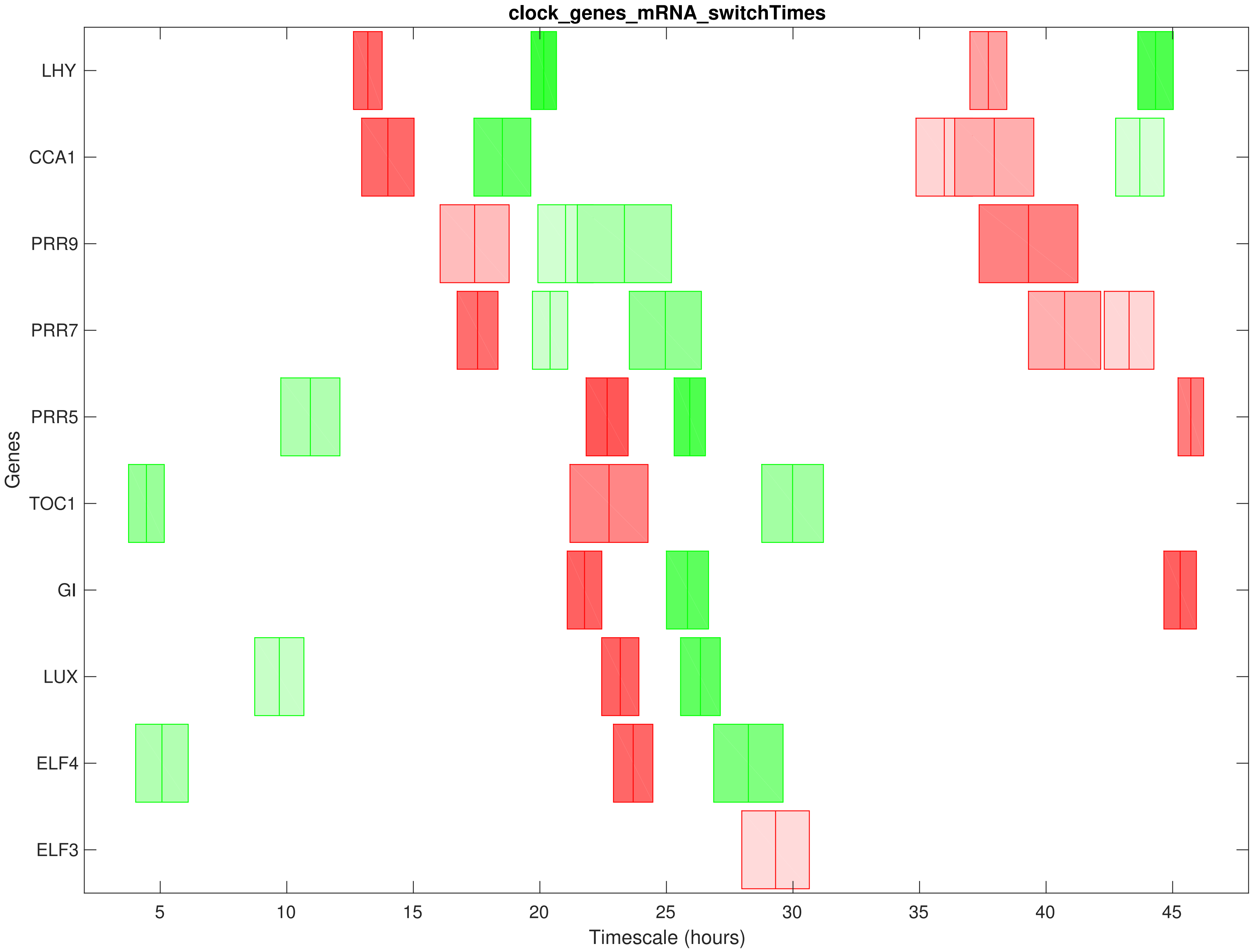

clock_genes_mRNA_switchTimes
LHY
CCA1
PRR9
PRR7
PRR5
TOC1
GI
LUX
ELF4
ELF3
Genes
5
10
15
20
25
30
35
40
45
Timescale (hours)
