# Supplementary material for: ReTrOS: a MATLAB toolbox for reconstructing transcriptional activity from gene and protein expression data
Source: BMC Bioinformatics. 2017 Jun 26;18:316. doi: 10.1186/s12859-017-1695-8 (PMC5485715; doi:10.1186/s12859-017-1695-8)
Supplement: Additional file 1 — Toolbox and data. The MATLAB code for running ReTrOS toolbox. This also includes the data used in the illustrative examples and the user manual that provides the instructions on how to run the toolbox. (ZIP 52838 kb) [file 12859_2017_1695_MOESM1_ESM.zip › ReTrOS-master/ReTrOSv5.4/ReTrOS user manual.docx]

ReTrOS user manual

Table of Contents

What is ReTrOS? 1

Running ReTrOS 1

Starting the ReTrOS graphical user interface 1

Select Data window 1

Algorithm Parameters window 2

Run Algorithm window 3

Using ReTrOS-smooth from the command-line 3

ReTrOS-smooth parameter structure 4

Using ReTrOS-switch from the command-line 4

ReTrOS-switch optional parameters 5

Expression data file format 6

ReTrOS output 6

# What is ReTrOS?

ReTrOS is a MATLAB toolbox that provides two algorithms for back-calculating gene transcription activity from either mRNA or protein expression time-series data.

# Running ReTrOS

ReTrOS can be run either through the graphical user interface (GUI) or directly from the command-line interface in MATLAB.

## Starting the ReTrOS graphical user interface

To run either method from the GUI, run from the toolbox root folder:

**ReTrOSGUI;**

There are 3 interface windows, allowing 1) selection of data and associated parameters, 2) algorithm parameters and 3) running selected algorithms.

### Select Data window

On starting the GUI the ‘Select Data’ window appears. In this window the user is able to select the expression data file (file format is described below), which is then imported and previewed in the window. There are 3 data parameters that can be changed by the user:

1. Whether to apply a de-trending algorithm to the input data (for instance to remove systematic damping effects caused by the measuring protocol) – there are two options ‘none’, or ‘log-linear’.
2. The type of expression data observed. There are two options ‘mRNA’, ‘protein reporter’. The distinction between ‘mRNA’ and ‘protein reporter’ is that in the reporter case the protocol uses a (typically fluorescent or luminescent protein) reporter construct to obtain measurement of the protein reporter expression rather than mRNA. This means we are not directly observing the mRNA or its protein of interest, and means degradation rates of the reporter system should be used rather than degradation rates of the actual protein.
3. The data column that contains the profile names the user wants to use.

An example of this window with an example data set loaded is shown in [Figure 1](#Ref273865682).


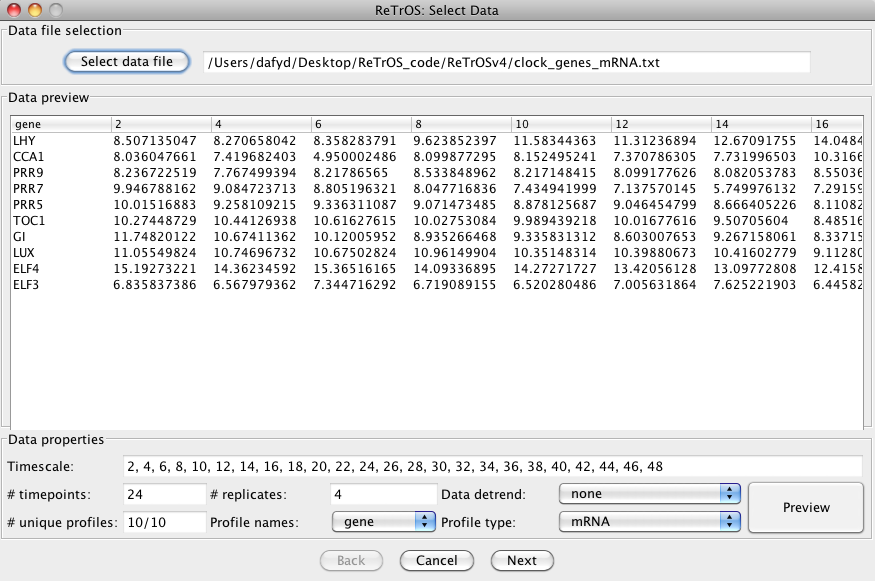


Figure 1

### Algorithm Parameters window

Next is the ‘Algorithm Parameters’ window. This window allows the user to select parameter values for most of the optional algorithm parameters. The degradation rate parameters apply to both algorithms, and allow the user to select the distribution (Normal or Gamma) and the associated parameters (mean and standard deviation for Normal; alpha and beta for Gamma). Three degradation rate options are provided:

1. ‘User input’ which allows the user to select the distribution type and parameter values.
2. ‘Arabidopsis’ which are mRNA and protein degradation rate distributions (Gamma) generated from large datasets of experimentally derived specific mRNA and protein degradation rates from the model plant *Arabidopsis thaliana*. This is the default for ‘mRNA’ and ‘protein (actual)’ expression data sets.
3. ‘luc reporter’ which are mRNA and protein degradation rate distributions (Normal) experimentally derived of the luciferase protein reporter system applied in *Arabidopsis thaliana.* This is the default for ‘protein reporter’ expression data sets.

These distributions will be used for all profiles, unless the ‘Use rates from file?’ box is checked and a valid file is selected. If the expression type is ‘protein reporter’, any protein degradation rates in the file are ignored, and any mRNA degradation rates are used as ‘native’ mRNA rates for the ‘smooth’ algorithm. Otherwise the mRNA/protein degradation rates are used individually for each matching profile. Parameters specific to each algorithm (with tooltip descriptions) are grouped in the tabbed panels, and default values are generated based on the input data, and the name of the analysis can be changed (defaults to the expression data file name).

An example of this window is shown in [Figure 2](#Ref273867499)


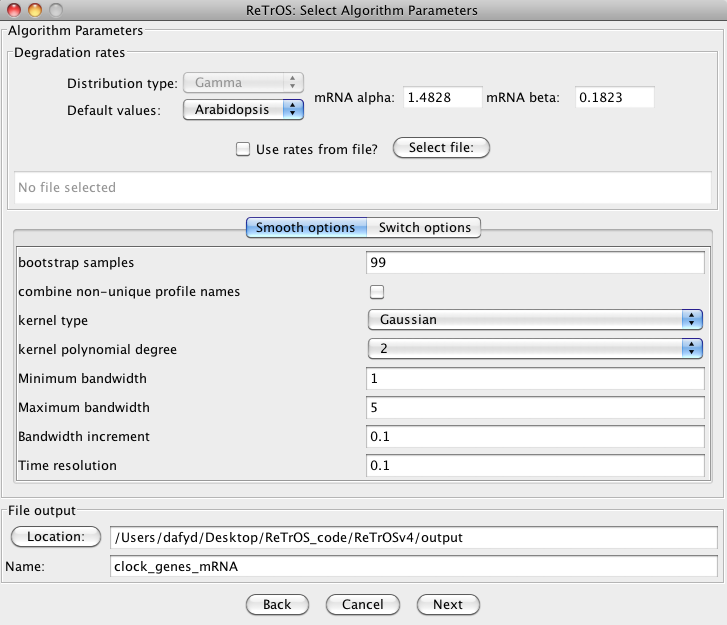


Figure 2

### Run Algorithm window

The final ‘Run Algorithm’ window simply allows the user to run none, one or both of the algorithms with the previously selected parameters. If one or both algorithms are run, the algorithm progress will be outputted to the MATLAB Command Window. Cancelling the algorithm will finish the current profile and then either move onto the next algorithm, or stop execution completely. Scripts to run the two algorithms separately are also generated by this window, and are placed in the ‘scripts’ folder. This allows the user to run the algorithms as a background process, or, with some minor modification of file paths, to run on other computers or servers.

## Using ReTrOS-smooth from the command-line

To run ReTrOS-smooth from the command-line, run from the ‘**smooth**’ folder:

**ReTrOSsmooth(paramStruct, data, time, native_mRNA_deg_mean, native_mRNA_deg_var, name);**

Where:

**paramStruct** is a structure containing the parameter values to use (see XXX for parameters),

**data** is a vector containing all observations (including replicates) for this profile,

**time** is a vector of the same length as **data** indicating the time (in hours) that the respective **data** element corresponds to,

**native_mRNA_deg_mean** is a positive scalar value corresponding to the mean degradation rate of the native mRNA transcript. Can be empty, **[]**,

**native_mRNA_deg_var** is a positive scalar value corresponding to the variance of the degradation rate of the native mRNA transcript. Can be empty, **[]**,

**name** is a string representing the name of the current profile (for example gene or protein name, specific line, etc.)

### ReTrOS-smooth parameter structure

The ReTrOS-smooth algorithm requires a parameter structure that defines a number of values that are used by the algorithm:

**expressionType** takes one of the following values ‘mRNA’, ‘protein (reporter)’ or ‘protein (actual)’ which defines what type of expression data is being used,

**bootstrapSamples** is a positive scalar value that determines the number of bootstrap samples to be generated by the algorithm (suggested default value of 99),

**detrend** takes one of the following values ‘none’, ‘linear’ or ‘log-linear’ which defines how to detrend the expression data,

**kernel** takes one of the following values ‘Ga’, ‘Ep’ or ‘Tr’ which defines which kernel to use to smooth the expression data. ‘Ga’ is the Gaussian kernel, ‘Ep’ is the Epanechnikov kernel and ‘Tr’ is the triweight kernel (suggested default value of ‘Ga’),

**polynomialDegree** is an integer between 1 and 3 which defines the degree of the polynomial used in the kernel smoothing regression (suggested default value of 1),

**reporterDegRates** is a 4-element vector defining the mean and standard deviation of the mRNA and protein observed. If mRNA expression is used then the protein degradation rates (elements 3 and 4) are set to 0 (estimates are provided for a luciferase reporter system in Arabidopsis thaliana),

**bandwidthRange** is a 3-element vector defining the minimum and maximum bandwidth values (in hours) to test for optimal smoothing fit. The third value is the increment size to use when searching for the optimal bandwidth,

**timeResolution** is a positive real scalar value which defines the time increment (in hours) used in the transcription back-calculation (suggested default value of 0.1).

## Using ReTrOS-switch from the command-line

To run ReTrOS-switch with mRNA expression data from the command-line, run from the ‘**switch**’ folder:

**ReTrOSswitch_mRNA(name,data,timescale,replicates,iterations,…);**

To run ReTrOS-switch with protein expression data from the command-line, run from the ‘**switch**’ folder:

**ReTrOSswitch_protein(name,data,timescale,replicates,iterations,…);**

Where:

**name** is a string representing the name of the current profile (for example gene or protein, specific line, etc.),

**data** is a vector containing all observations (including replicates) for this profile and is of length **replicates** x length of **time**, e.g. if we have 4 **replicates** and a **time** consisting of 10 timepoints, **data** must be of length 40,

**time** is a vector containing the unique timing (in hours) of each observation. Note that if 2 or more observations have the same timing (are replicate samples), then the time is only included once in the **time** vector,

**replicates** is a scalar positive integer value and represents the maximum number of replicate samples for any observation. Note that the number of replicate samples may vary for each observation, but the **replicates** parameter records only the maximum of these,

**iterations** is a scalar positive integer value and is the number of Markov chain Monte Carlo iterations to run,

**…** are optional ‘parameter-value’ pairs. See below for description of optional parameters and their corresponding expected values.

### ReTrOS-switch optional parameters

All ReTrOS-switch optional parameters must be supplied with a corresponding value to form a ‘parameter-value’ pair. For example to turn off all figure plotting functionality the ‘parameter-value’ pair **‘showplot’, false** is added to the end of the function call input arguments.

Parameters are valid for both ReTrOS-switch functions unless stated, and all parameters have either a fixed default value, or one calculated from the input expression data and timescale. The supported parameters (as strings) and their corresponding value type are:

**‘showplot’, <logical scalar>** - if we want to view the raw output after analysis (default: true)

**‘plotSamples’, <logical scalar>** - if we want to plot all sample data, or just the posteriors (default: true) NOTE: only used if 'showplot' is true

**‘plotSwitchFitting’, <logical scalar>** - if we want to plot various stages of the getSwitchFit function (default: false) NOTE: only used if 'showplot' is true

**‘plotDegradationDist’, <logical scalar>** - if we want to plot estimated degradation rate distribution against the prior distribution (default: false) NOTE: only used if **'showplot'** is true

**‘minSwitchStrength’, <real scalar>** - sets the minimum switch strength in the returned switch distribution (default: 0.2)

**‘switchEvidence’, <real scalar>** - sets the Bayes factor evidence threshold suggesting an N-switch model compared to a 0-switch model (default: 0)

**‘mRNADegradationRatePrior’, <real scalar>** - sets the alpha and beta parameters of the Gamma prior distribution for the mRNA degradation rate (default: [1.4828 0.1823])

**‘proteinDegradationRatePrior’, <real scalar>** - sets the alpha and beta parameters of the Gamma prior distribution for the protein degradation rate (default: [3.3765 0.0822]). Note this parameter is only valid for the **ReTrOSswitch_protein** function.

**‘dataDetrend’, <string>** - sets the data detrending method (default: ‘none’)

**‘useInitialSwitchSuggest’, <logical scalar>** - if we want to start with an initial guess of switch points (based on changes in gradient of expression level) (default:true)

**‘maxSamplesToUse’, <integer scalar>** - sets the maximum number of accepted MCMC samples to use to estimate posterior distributions. Samples are taken uniformly from accepted samples (default: all)

**‘maxSamplesToPlot’, <integer scalar>** - sets the maximum number of accepted MCMC samples plotted in figures (default: 10000)

**‘maxSwitches’, <integer scalar>** - sets the maximum number of switch points allowed by the algorithm (default: # of timepoints / 3)

**‘switchNumberPrior ‘,<integer scalar>** - sets the mean number of switch points to expect (default: 1)

**‘switchDelay’, <real scalar>** - sets the minimum delay between switch points (default: minimum time between timepoints)

**‘switchBaselineFactor’, <real scalar>** - sets the scaling factor of the switch density baseline modifier (default: 1)

**‘confidence’, <integer scalar>** - sets the confidence (number of standard deviations) at which to generate switch distributions (only used in plots) (default: 3)

**‘burnIn’, <real scalar>** - sets the proportion of samples from chain to remove as the 'burn in' period from beginning of chain (default: 0.25)

**‘percentiles’, <real vector>** - sets the percentiles to return sigma and degradation rate estimates (default: [0.5 1 2.5 5 95 97.5 99 9.5])

# Expression data file format

The data format used is a tab-delimited table that can easily be generated using standard spreadsheet software. A header row defines the observation times, and each row contains the observed data for a single sample, gene or protein. The first N columns in the table can be used to specify the name of the gene/protein or other annotation data and the remaining columns contain the observed values for the corresponding observation time in the header row. Replicate samples can be either treated as individual time-series, or combined together.

# ReTrOS output

There are 2 types of output from the ReTrOS algorithms:

1. Plots of each profile model fit
2. MATLAB structures containing model details

In addition, the GUI (and generated script files) will:

1. Save plots as PNG images and PDF documents, and combine PDFs into a single document (platform dependent functionality)
2. Save MATLAB structures for model outputs into a single .mat file.
3. Output specific algorithm parameters as text files.
