# Supplementary figures and images for: ReTrOS: a MATLAB toolbox for reconstructing transcriptional activity from gene and protein expression data
Source: BMC Bioinformatics. 2017 Jun 26;18:316. doi: 10.1186/s12859-017-1695-8 (PMC5485715; doi:10.1186/s12859-017-1695-8)

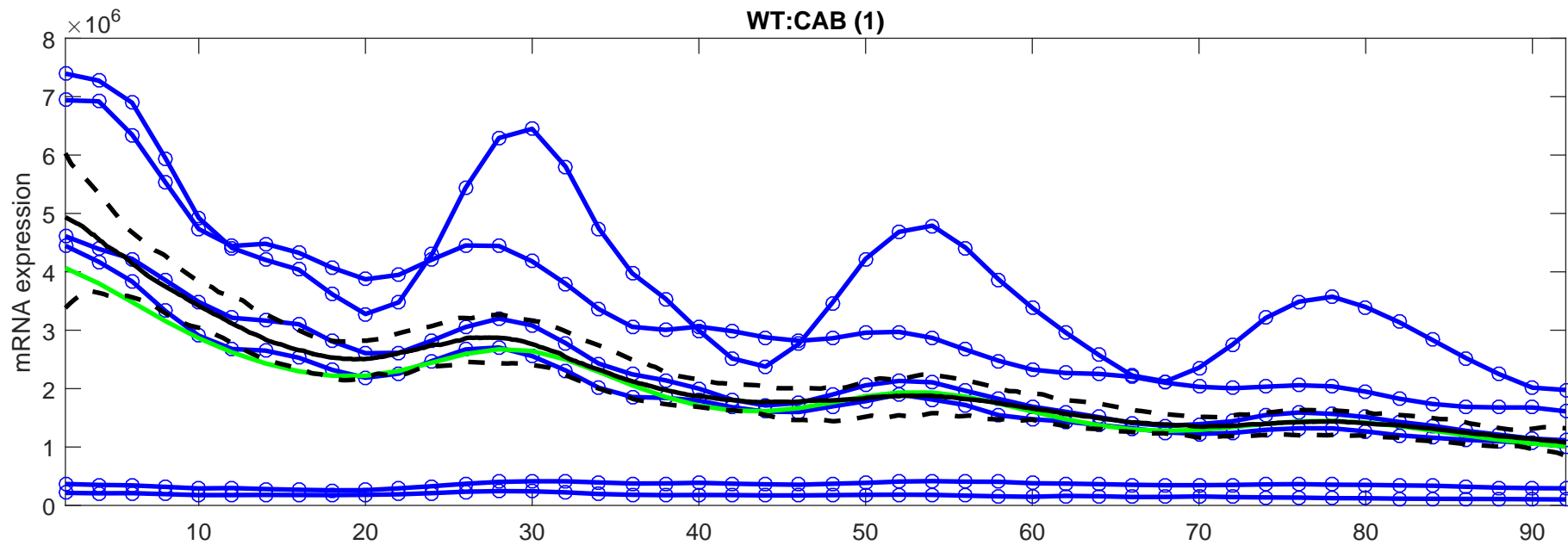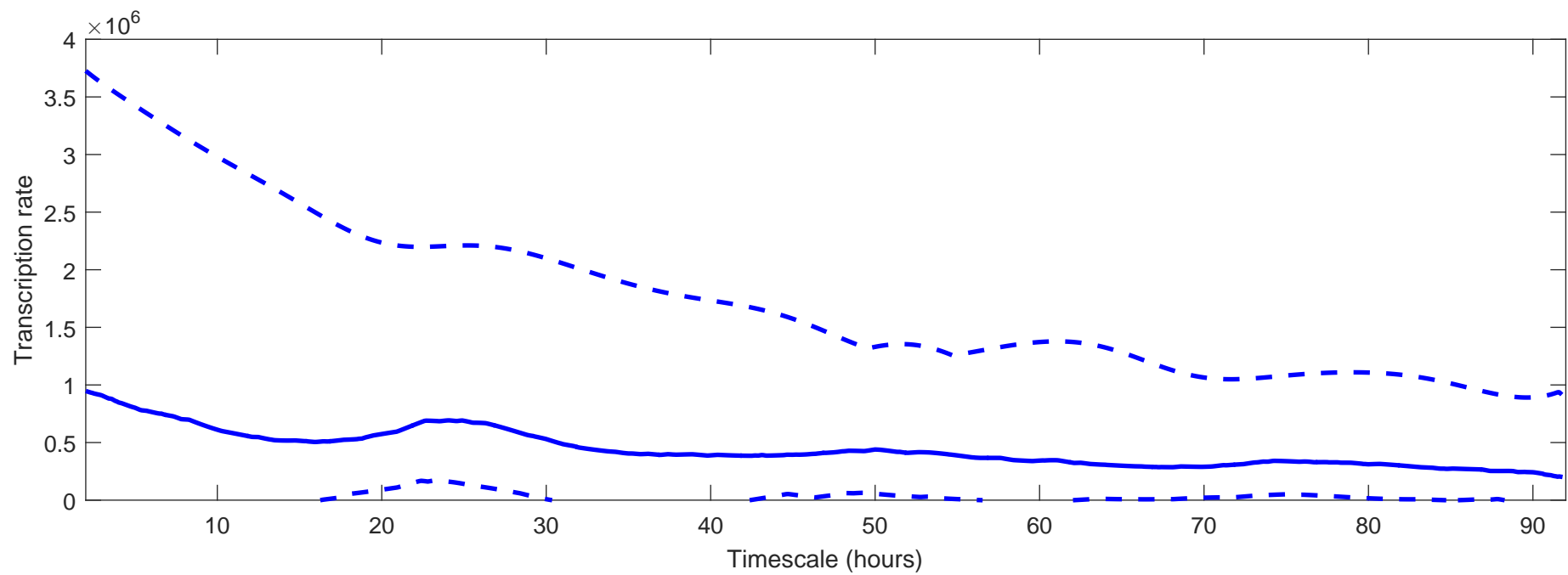

Supplement: Additional file 1 — Toolbox and data. The MATLAB code for running ReTrOS toolbox. This also includes the data used in the illustrative examples and the user manual that provides the instructions on how to run the toolbox. (ZIP 52838 kb) [file 12859_2017_1695_MOESM1_ESM.zip › ReTrOS-master/ReTrOSv5.4/output/17C_RBL/plots_smooth/WT_CAB (1).pdf]

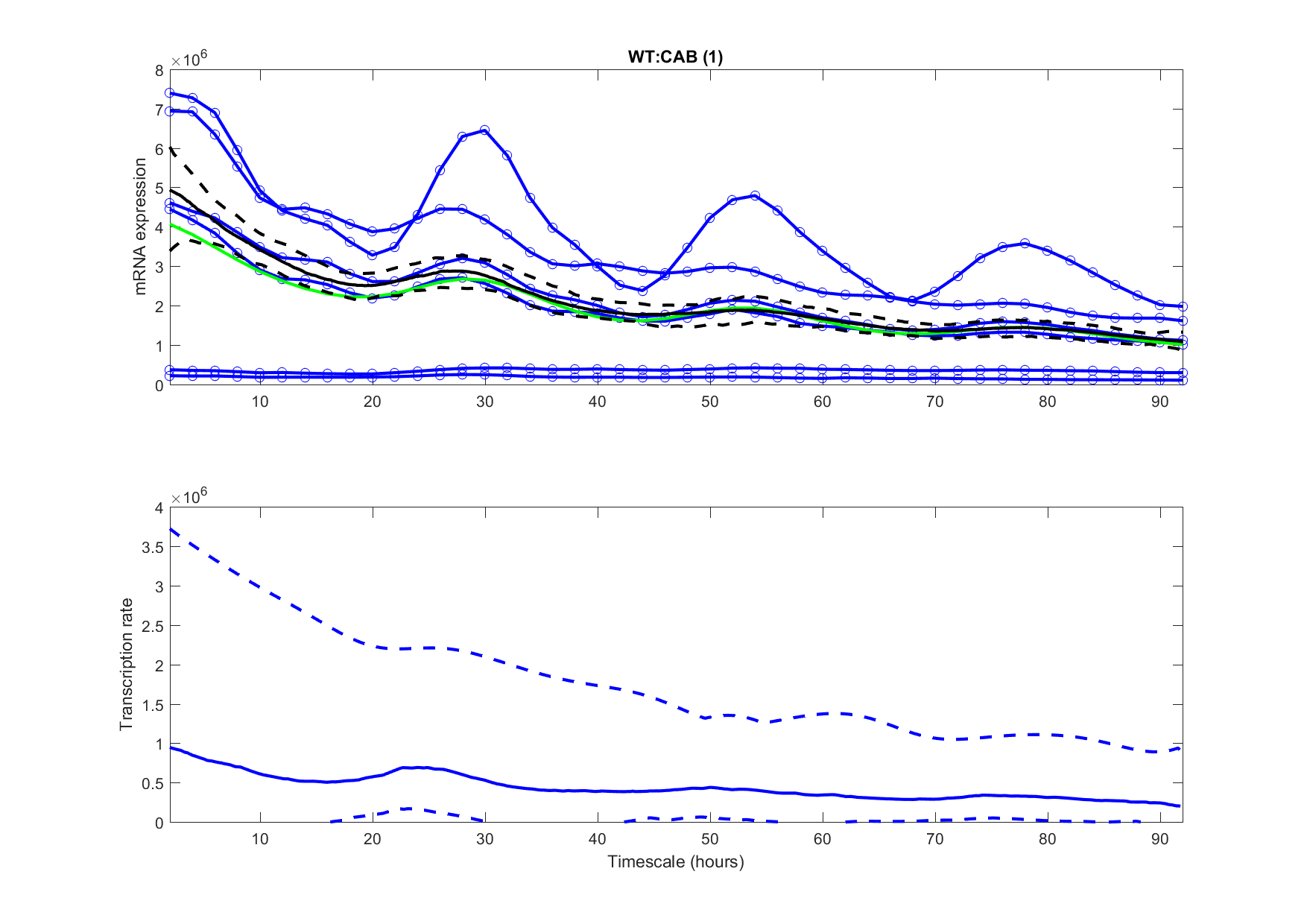

Supplement: Additional file 1 — Toolbox and data. The MATLAB code for running ReTrOS toolbox. This also includes the data used in the illustrative examples and the user manual that provides the instructions on how to run the toolbox. (ZIP 52838 kb) [file 12859_2017_1695_MOESM1_ESM.zip › ReTrOS-master/ReTrOSv5.4/output/17C_RBL/plots_smooth/WT_CAB (1).png]

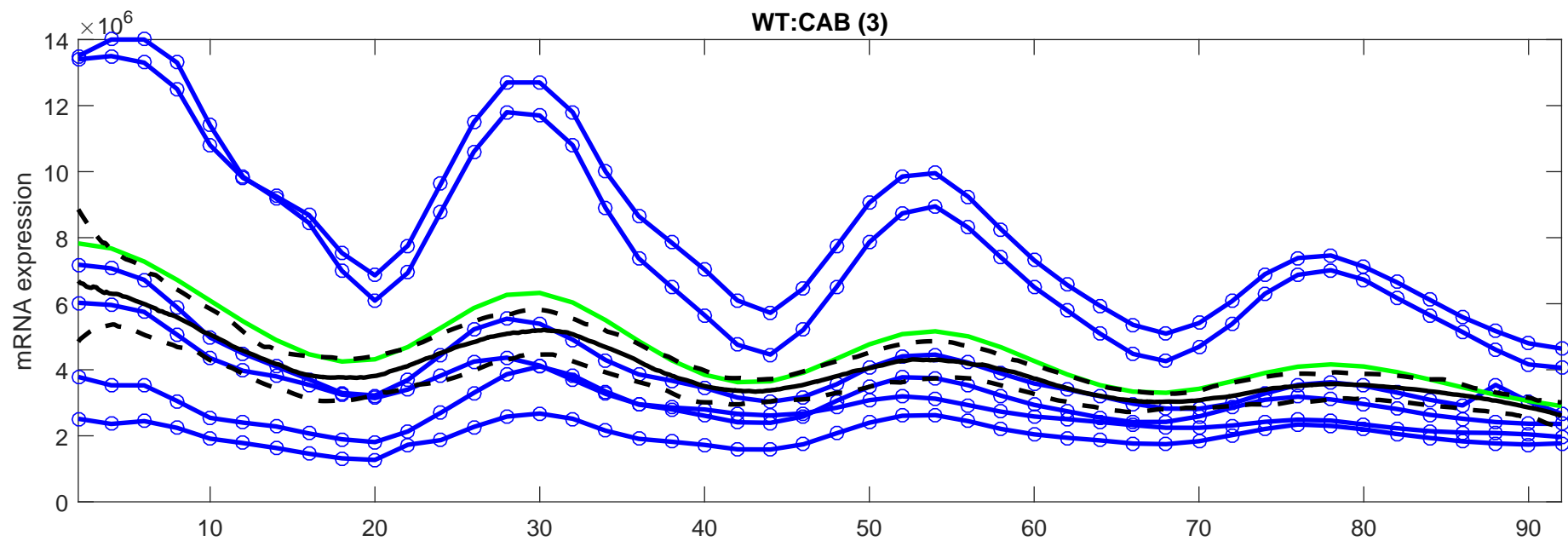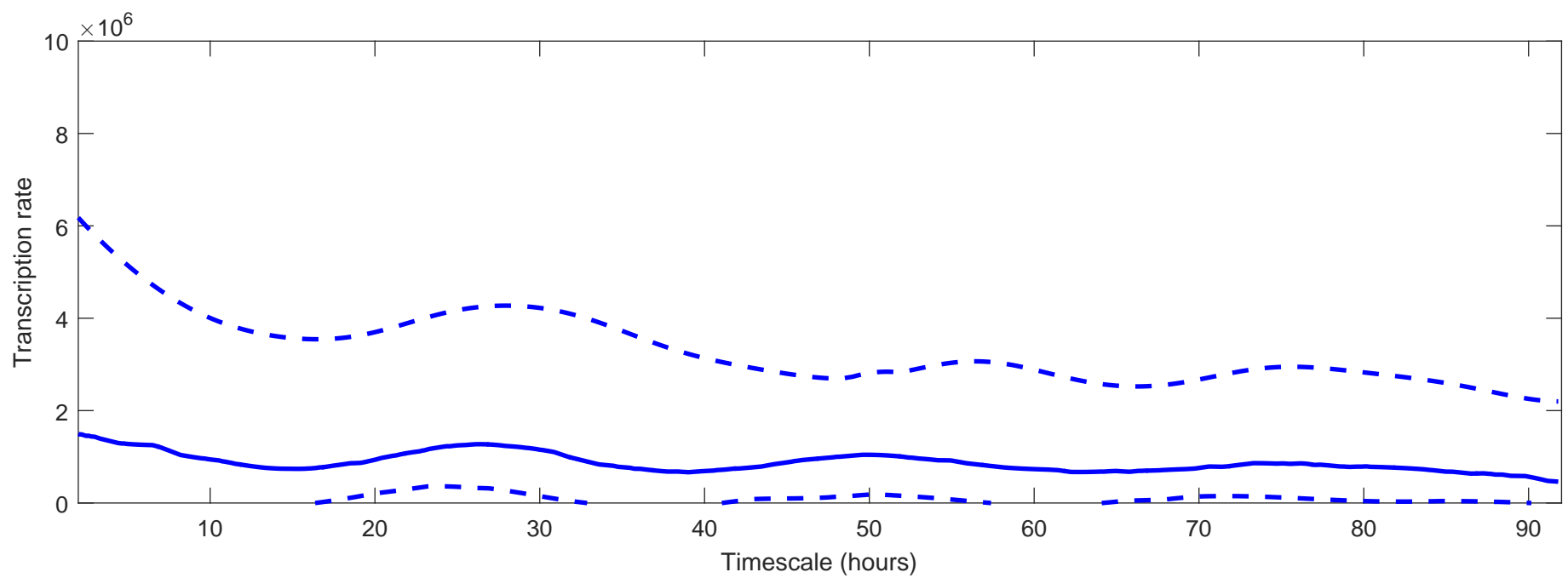

Supplement: Additional file 1 — Toolbox and data. The MATLAB code for running ReTrOS toolbox. This also includes the data used in the illustrative examples and the user manual that provides the instructions on how to run the toolbox. (ZIP 52838 kb) [file 12859_2017_1695_MOESM1_ESM.zip › ReTrOS-master/ReTrOSv5.4/output/17C_RBL/plots_smooth/WT_CAB (3).pdf]

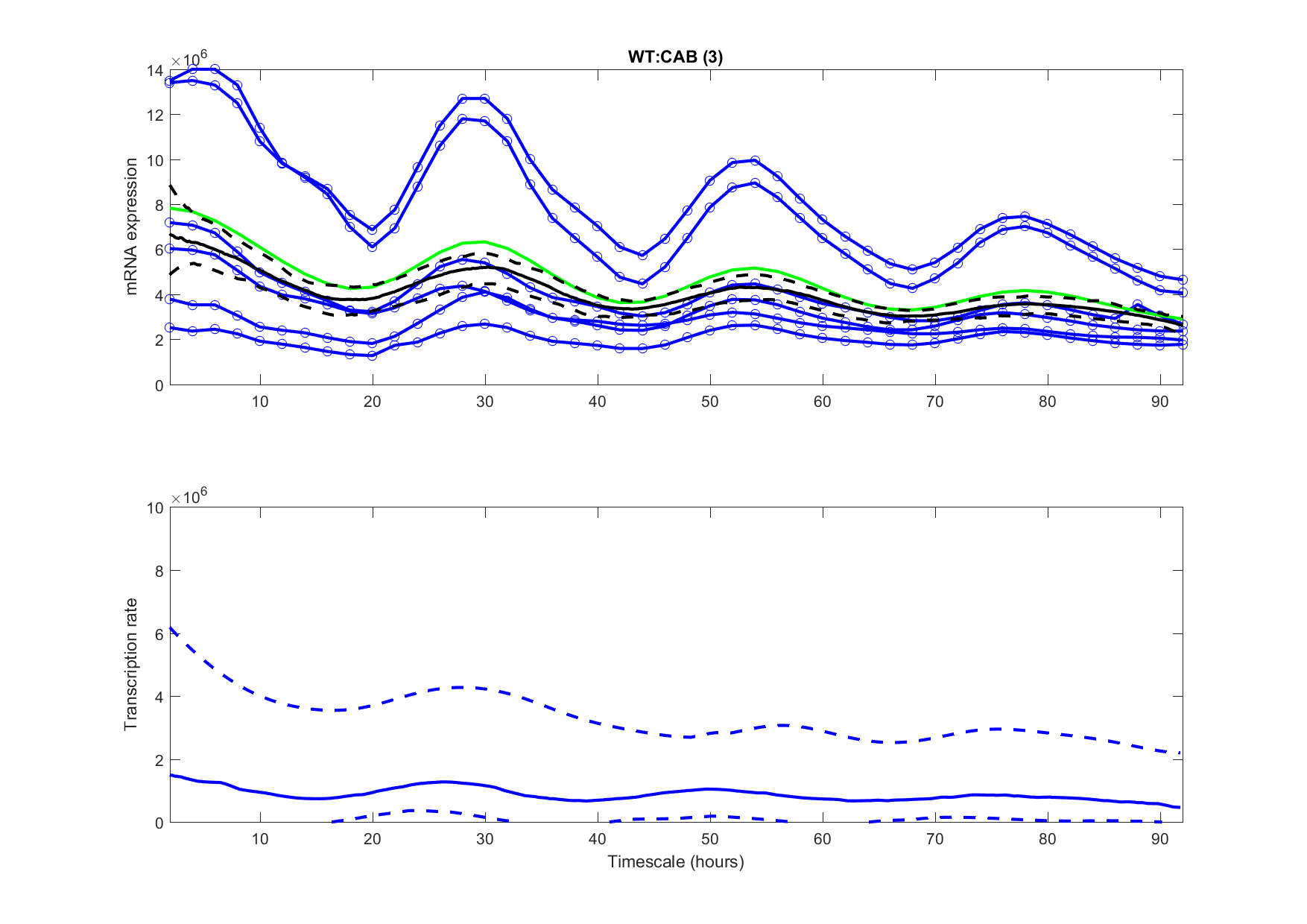

Supplement: Additional file 1 — Toolbox and data. The MATLAB code for running ReTrOS toolbox. This also includes the data used in the illustrative examples and the user manual that provides the instructions on how to run the toolbox. (ZIP 52838 kb) [file 12859_2017_1695_MOESM1_ESM.zip › ReTrOS-master/ReTrOSv5.4/output/17C_RBL/plots_smooth/WT_CAB (3).png]

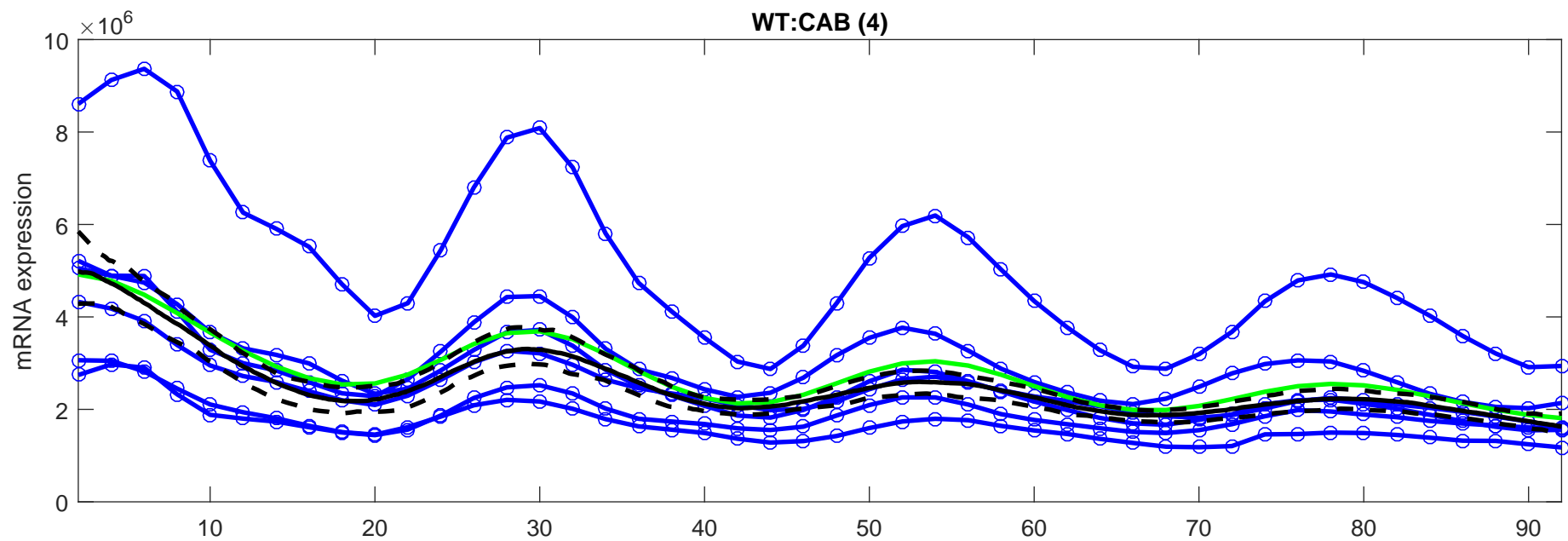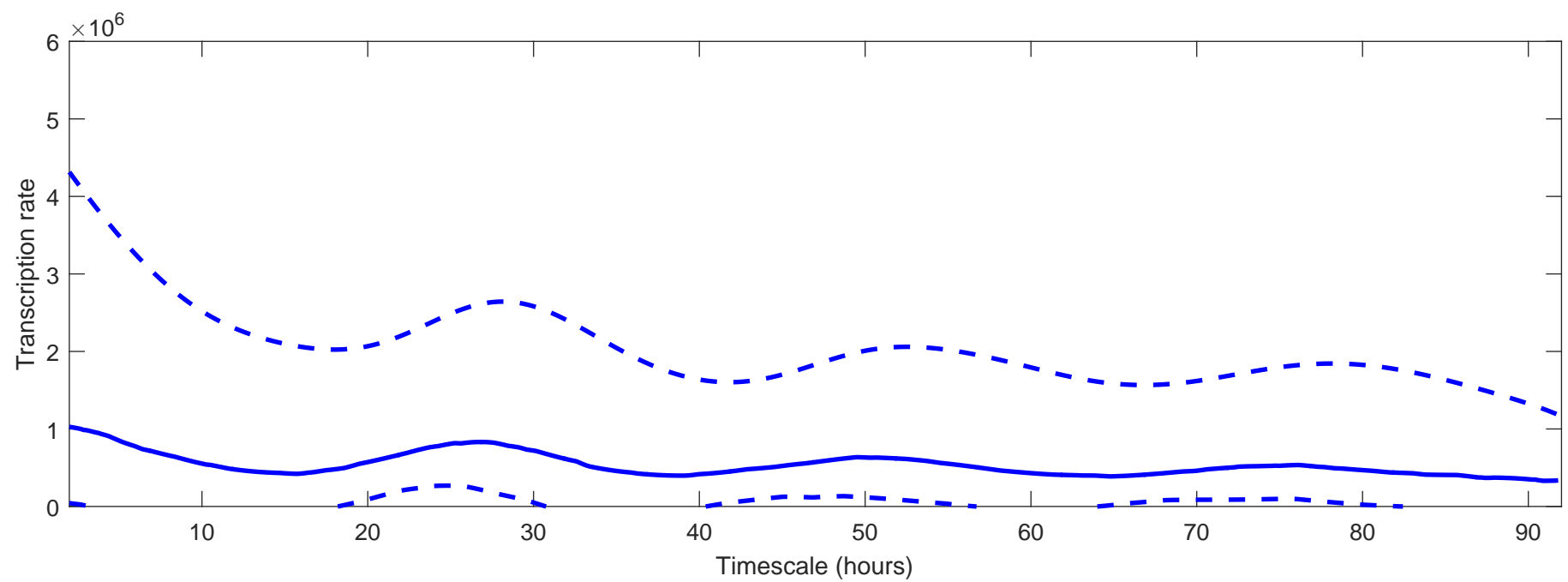

Supplement: Additional file 1 — Toolbox and data. The MATLAB code for running ReTrOS toolbox. This also includes the data used in the illustrative examples and the user manual that provides the instructions on how to run the toolbox. (ZIP 52838 kb) [file 12859_2017_1695_MOESM1_ESM.zip › ReTrOS-master/ReTrOSv5.4/output/17C_RBL/plots_smooth/WT_CAB (4).pdf]

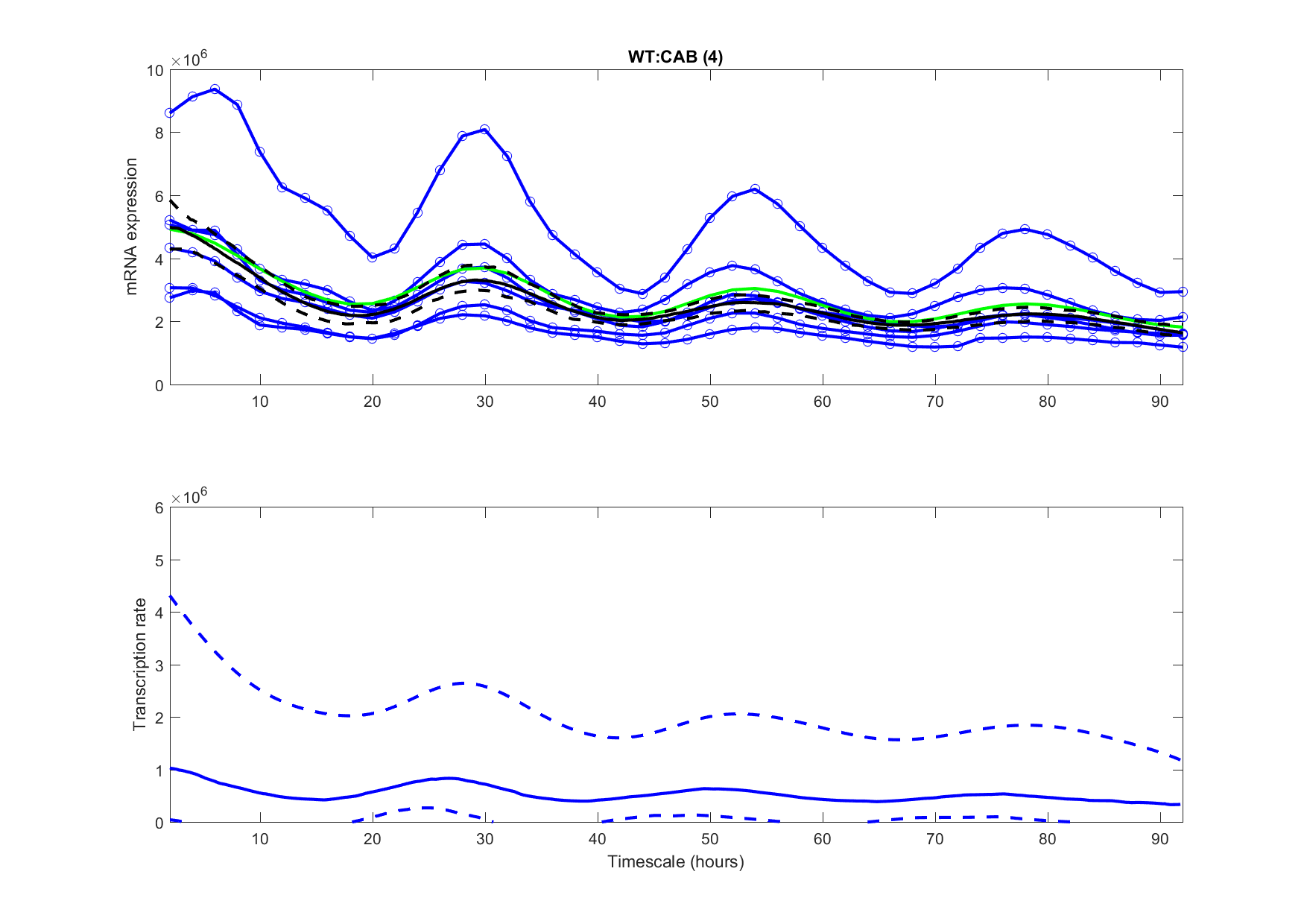

Supplement: Additional file 1 — Toolbox and data. The MATLAB code for running ReTrOS toolbox. This also includes the data used in the illustrative examples and the user manual that provides the instructions on how to run the toolbox. (ZIP 52838 kb) [file 12859_2017_1695_MOESM1_ESM.zip › ReTrOS-master/ReTrOSv5.4/output/17C_RBL/plots_smooth/WT_CAB (4).png]

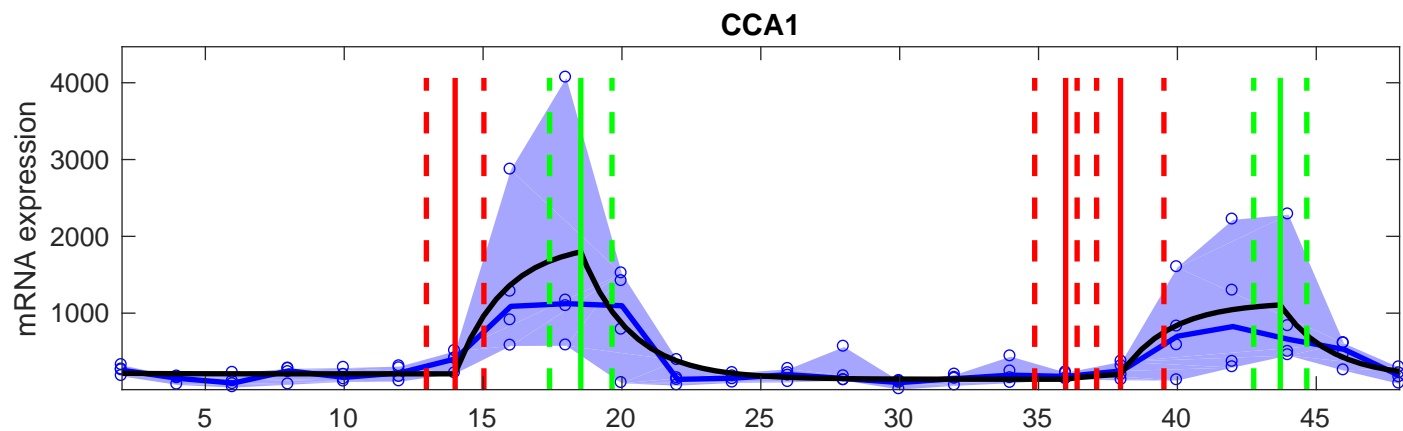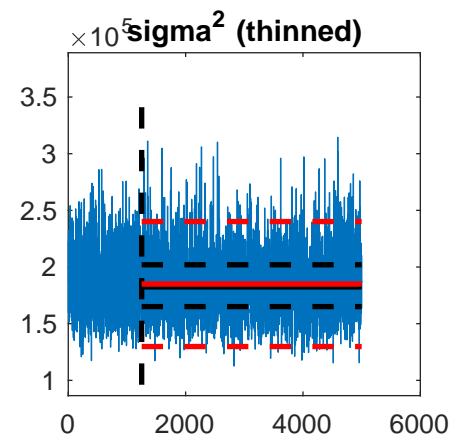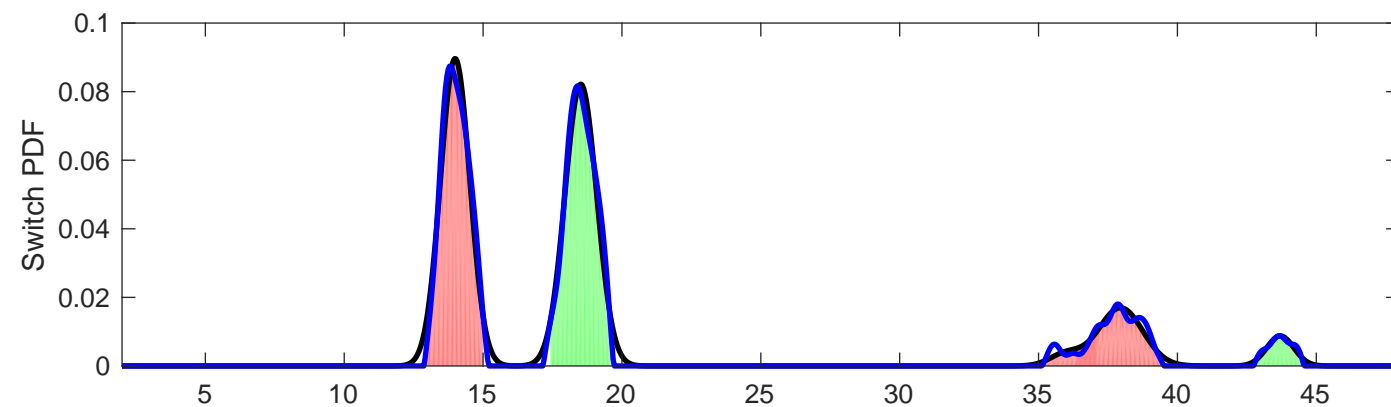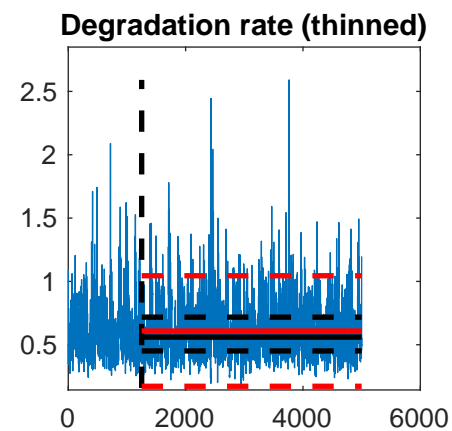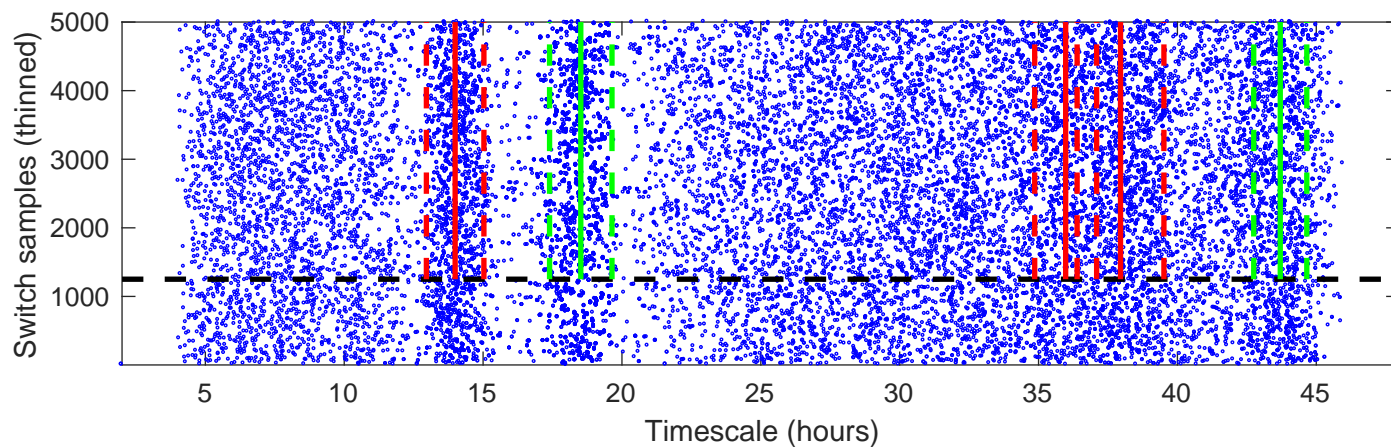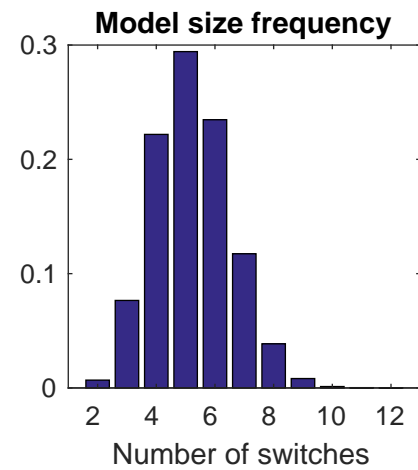

Supplement: Additional file 1 — Toolbox and data. The MATLAB code for running ReTrOS toolbox. This also includes the data used in the illustrative examples and the user manual that provides the instructions on how to run the toolbox. (ZIP 52838 kb) [file 12859_2017_1695_MOESM1_ESM.zip › ReTrOS-master/ReTrOSv5.4/output/clock_genes_mRNA/plots_switch/CCA1.pdf]

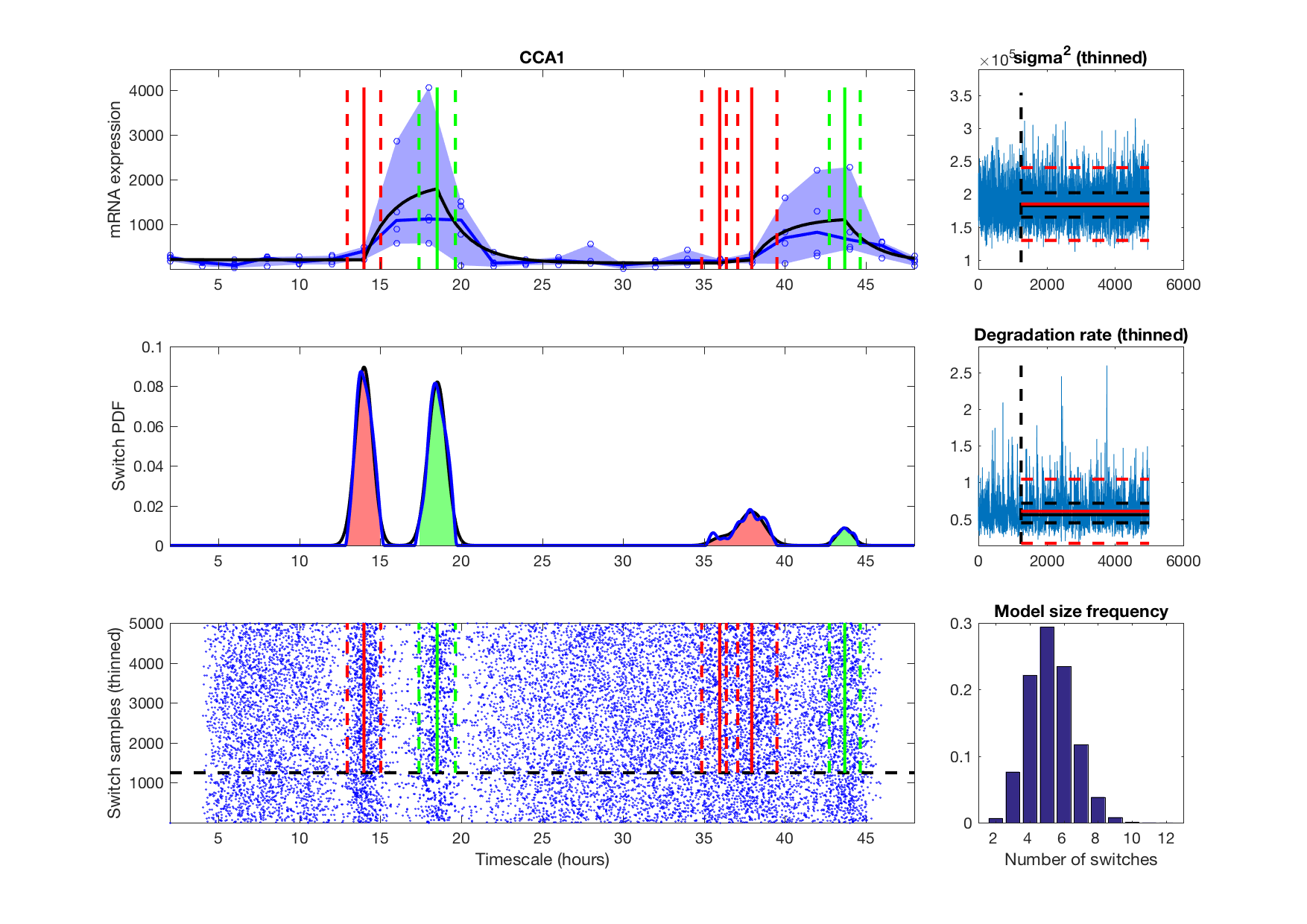

Supplement: Additional file 1 — Toolbox and data. The MATLAB code for running ReTrOS toolbox. This also includes the data used in the illustrative examples and the user manual that provides the instructions on how to run the toolbox. (ZIP 52838 kb) [file 12859_2017_1695_MOESM1_ESM.zip › ReTrOS-master/ReTrOSv5.4/output/clock_genes_mRNA/plots_switch/CCA1.png]

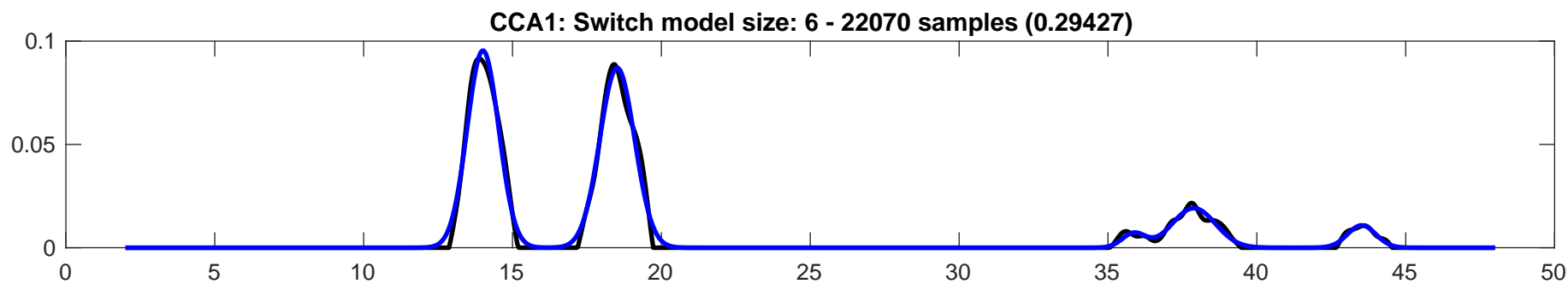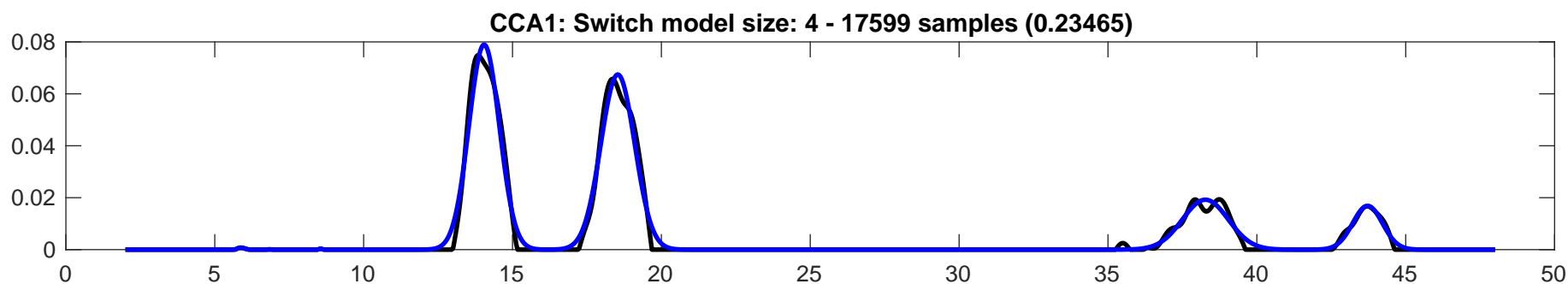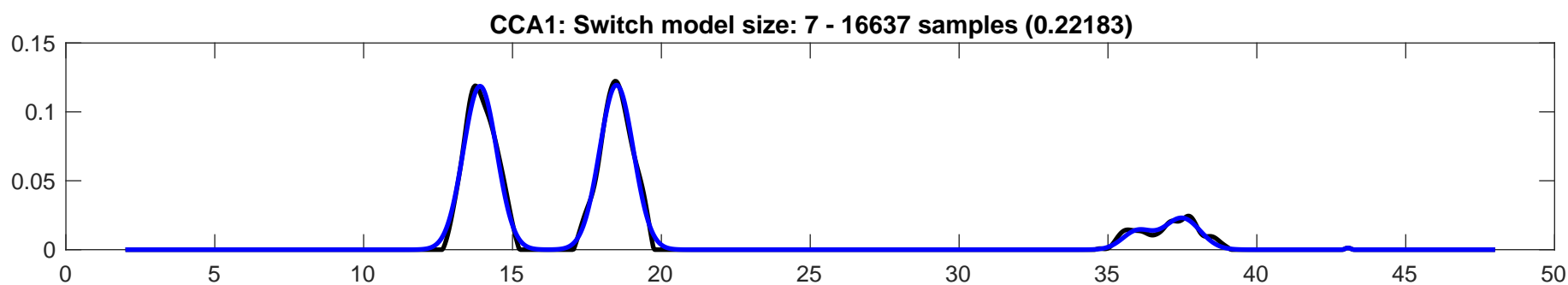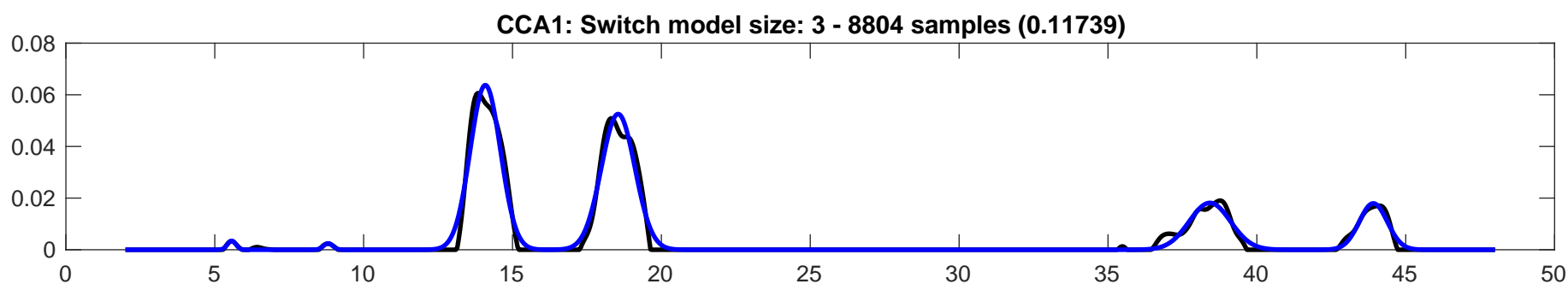

Timescale (hours)

Supplement: Additional file 1 — Toolbox and data. The MATLAB code for running ReTrOS toolbox. This also includes the data used in the illustrative examples and the user manual that provides the instructions on how to run the toolbox. (ZIP 52838 kb) [file 12859_2017_1695_MOESM1_ESM.zip › ReTrOS-master/ReTrOSv5.4/output/clock_genes_mRNA/plots_switch/CCA1_models.pdf]

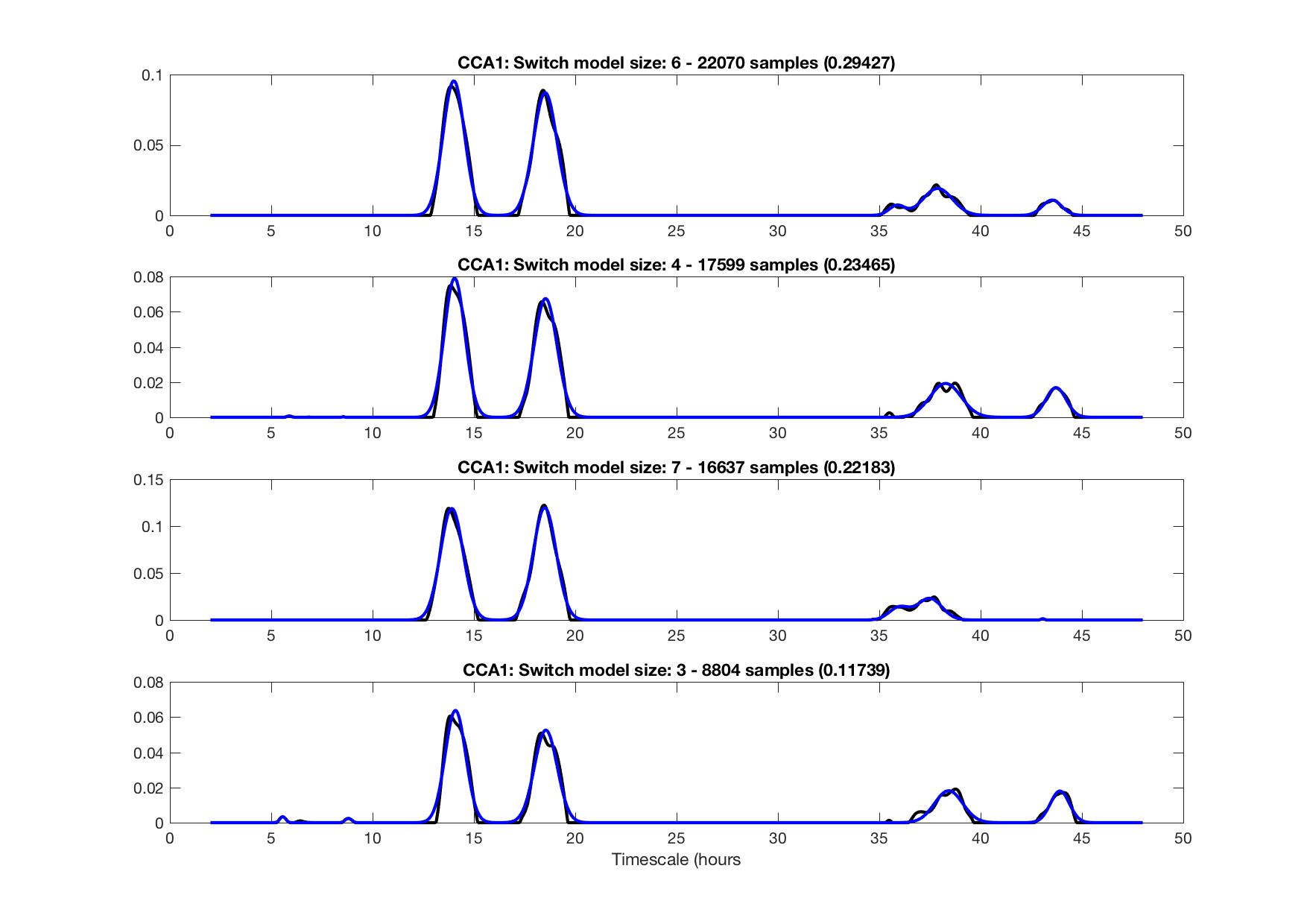

Supplement: Additional file 1 — Toolbox and data. The MATLAB code for running ReTrOS toolbox. This also includes the data used in the illustrative examples and the user manual that provides the instructions on how to run the toolbox. (ZIP 52838 kb) [file 12859_2017_1695_MOESM1_ESM.zip › ReTrOS-master/ReTrOSv5.4/output/clock_genes_mRNA/plots_switch/CCA1_models.png]

**ELF3**

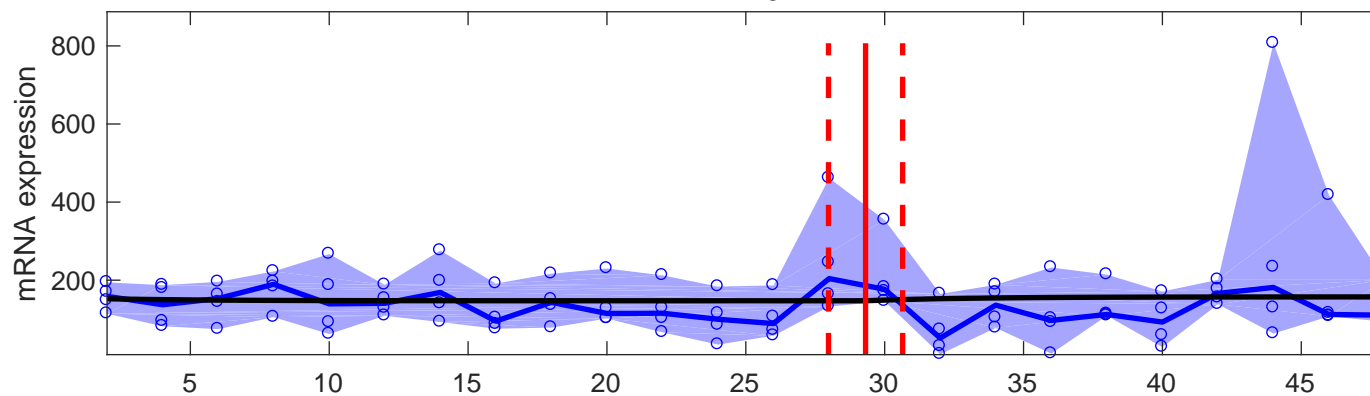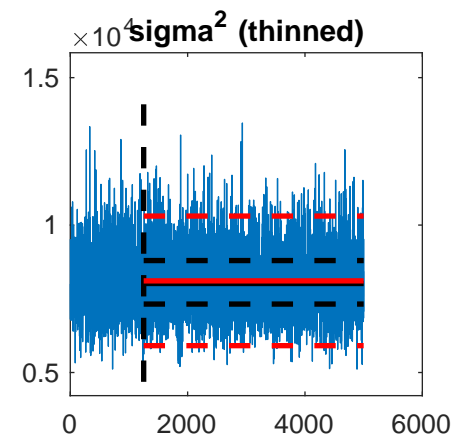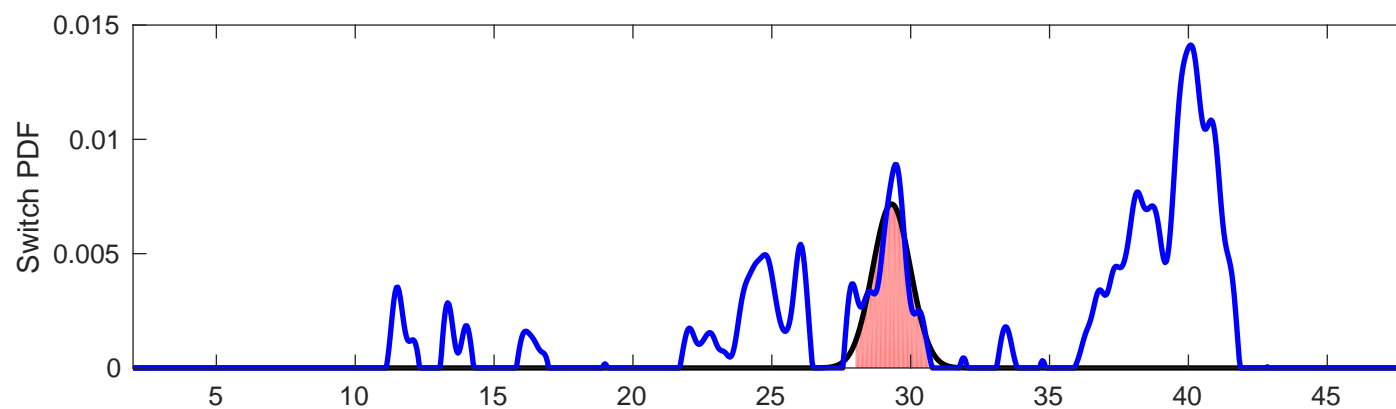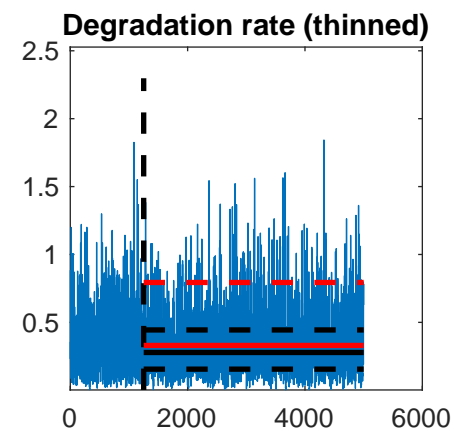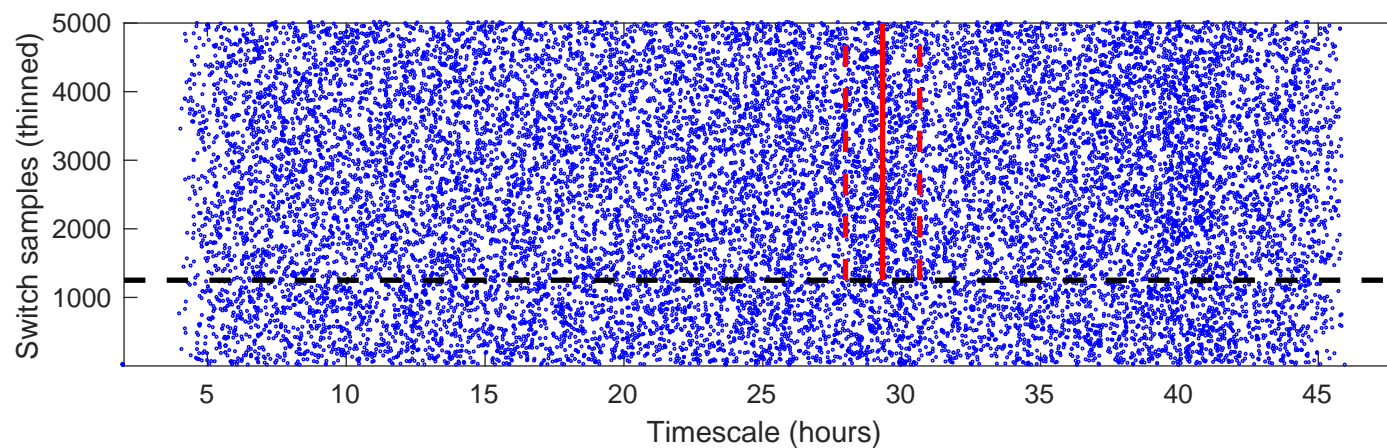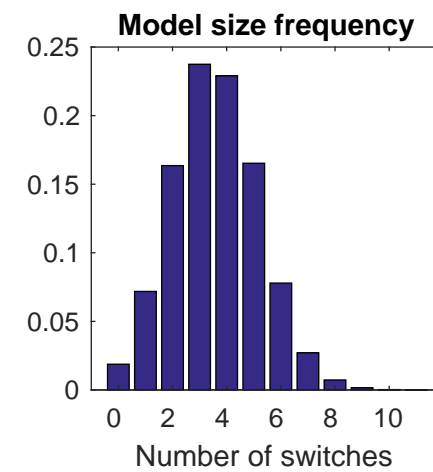

Supplement: Additional file 1 — Toolbox and data. The MATLAB code for running ReTrOS toolbox. This also includes the data used in the illustrative examples and the user manual that provides the instructions on how to run the toolbox. (ZIP 52838 kb) [file 12859_2017_1695_MOESM1_ESM.zip › ReTrOS-master/ReTrOSv5.4/output/clock_genes_mRNA/plots_switch/ELF3.pdf]

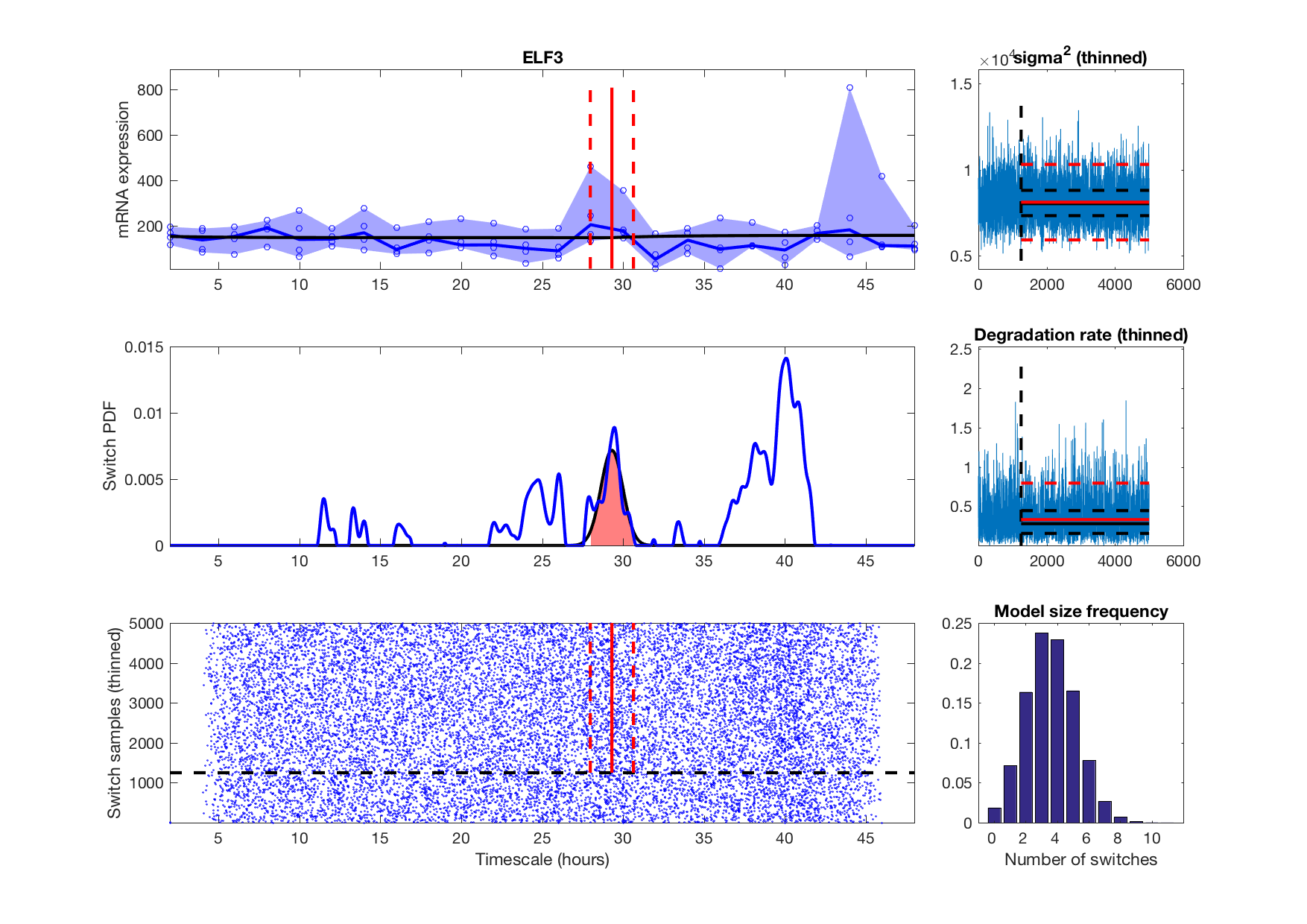

Supplement: Additional file 1 — Toolbox and data. The MATLAB code for running ReTrOS toolbox. This also includes the data used in the illustrative examples and the user manual that provides the instructions on how to run the toolbox. (ZIP 52838 kb) [file 12859_2017_1695_MOESM1_ESM.zip › ReTrOS-master/ReTrOSv5.4/output/clock_genes_mRNA/plots_switch/ELF3.png]

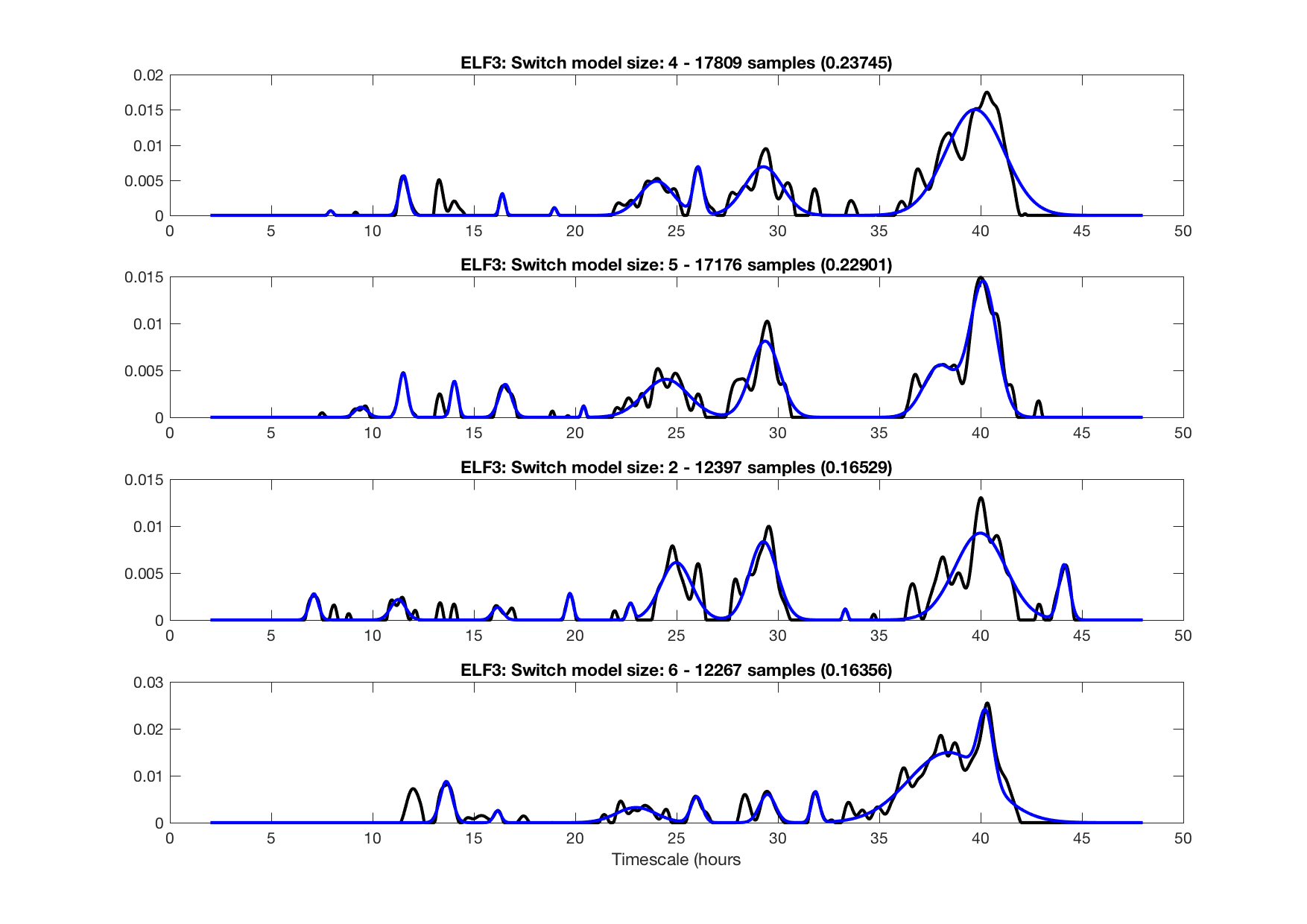

Supplement: Additional file 1 — Toolbox and data. The MATLAB code for running ReTrOS toolbox. This also includes the data used in the illustrative examples and the user manual that provides the instructions on how to run the toolbox. (ZIP 52838 kb) [file 12859_2017_1695_MOESM1_ESM.zip › ReTrOS-master/ReTrOSv5.4/output/clock_genes_mRNA/plots_switch/ELF3_models.png]

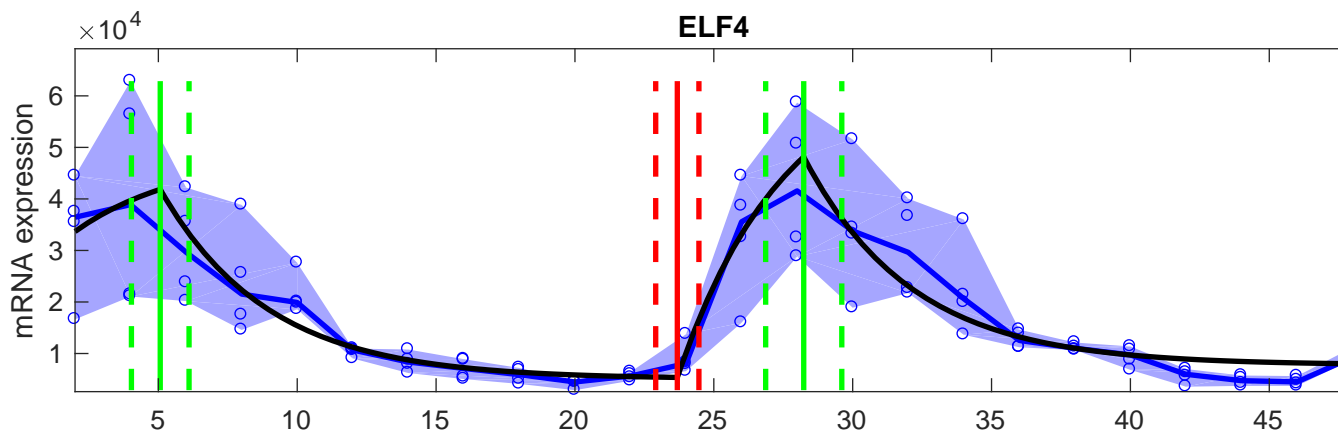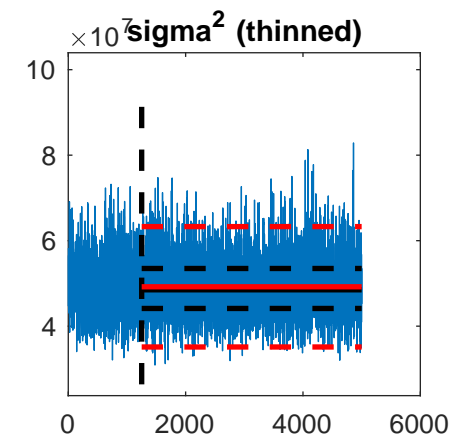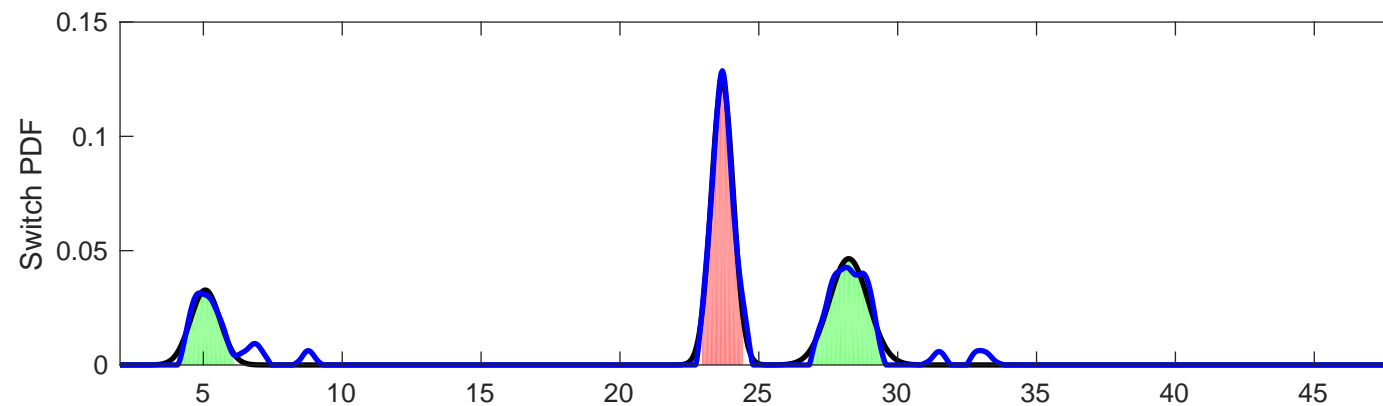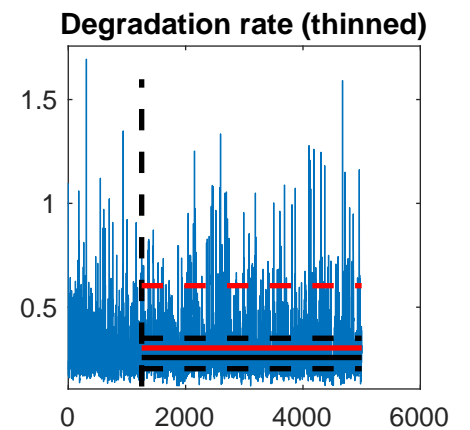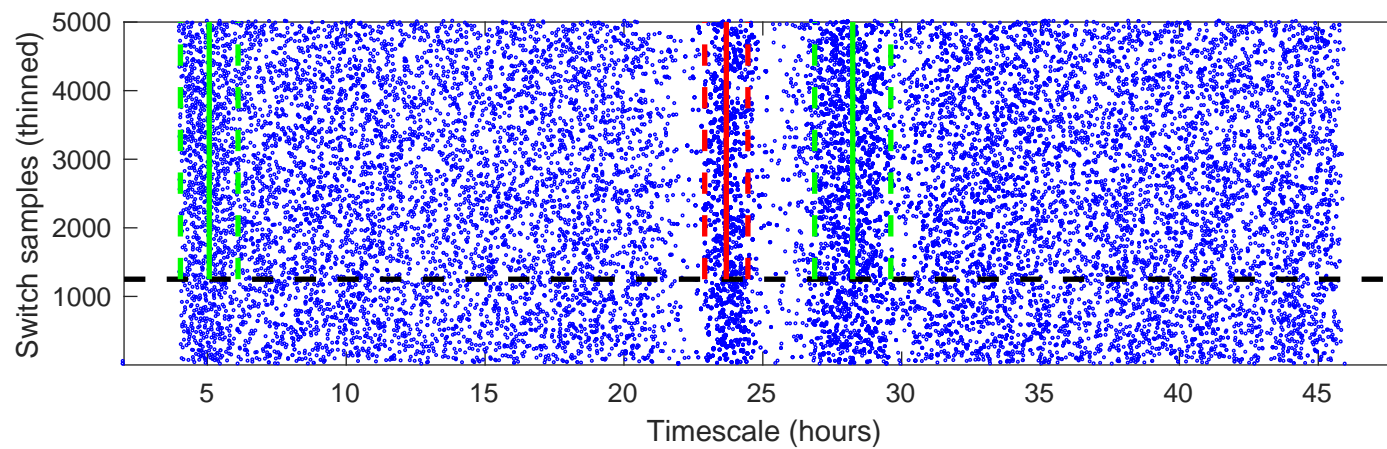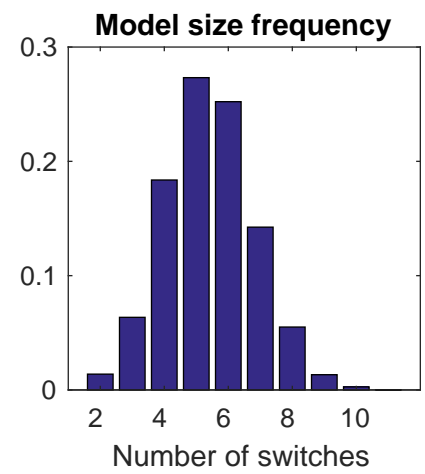

Supplement: Additional file 1 — Toolbox and data. The MATLAB code for running ReTrOS toolbox. This also includes the data used in the illustrative examples and the user manual that provides the instructions on how to run the toolbox. (ZIP 52838 kb) [file 12859_2017_1695_MOESM1_ESM.zip › ReTrOS-master/ReTrOSv5.4/output/clock_genes_mRNA/plots_switch/ELF4.pdf]

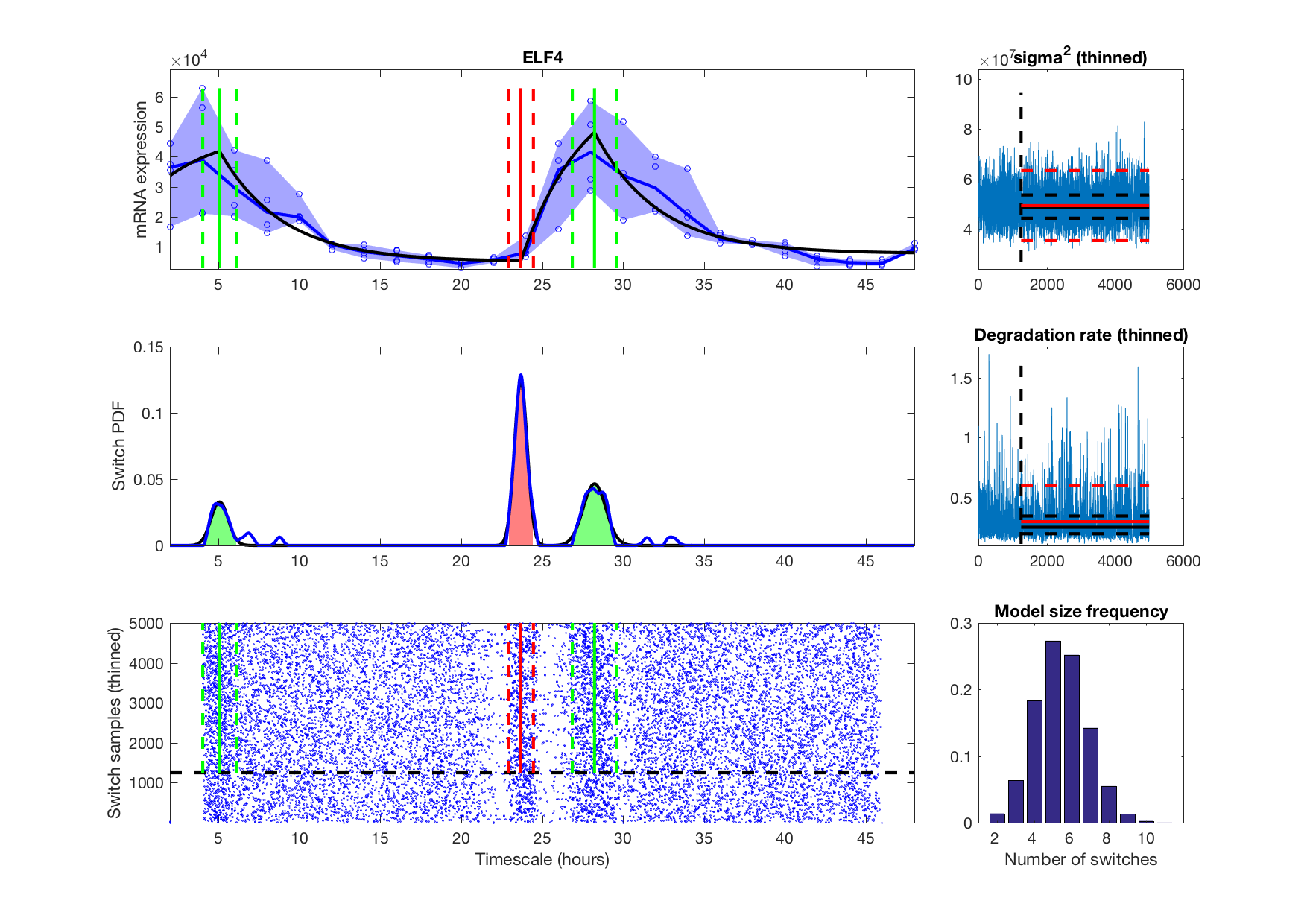

Supplement: Additional file 1 — Toolbox and data. The MATLAB code for running ReTrOS toolbox. This also includes the data used in the illustrative examples and the user manual that provides the instructions on how to run the toolbox. (ZIP 52838 kb) [file 12859_2017_1695_MOESM1_ESM.zip › ReTrOS-master/ReTrOSv5.4/output/clock_genes_mRNA/plots_switch/ELF4.png]

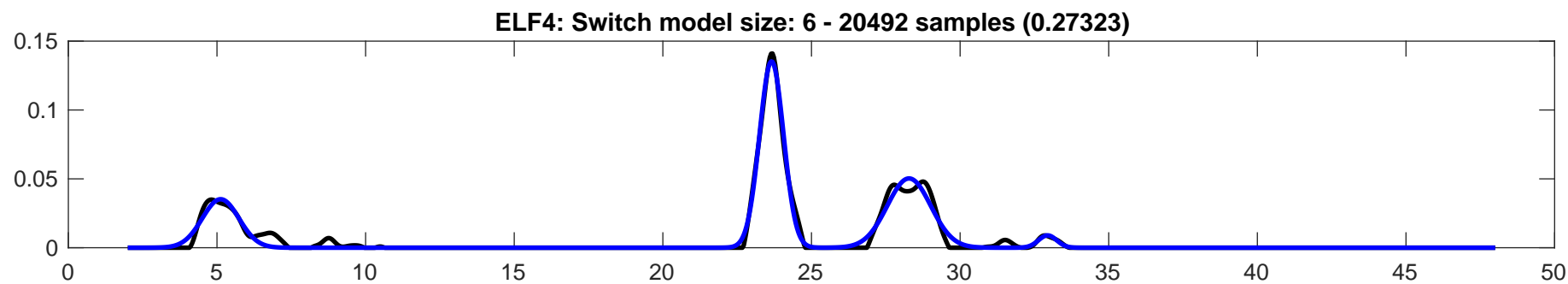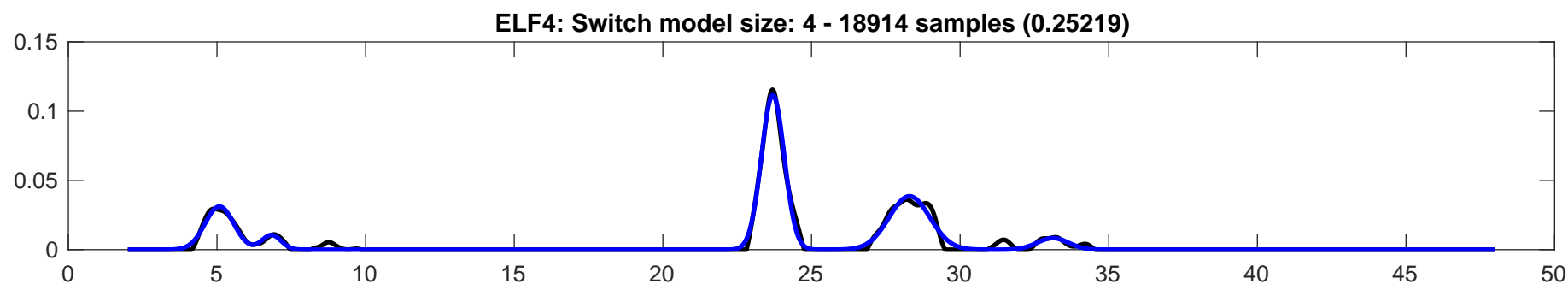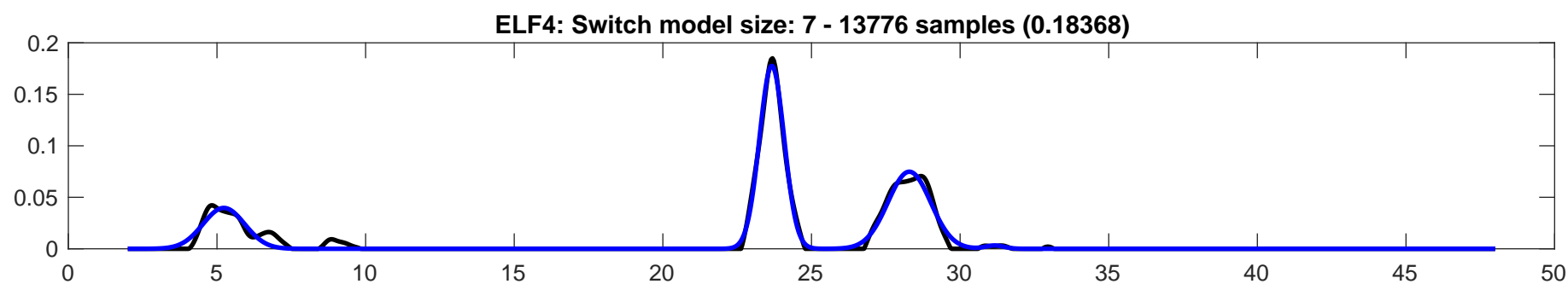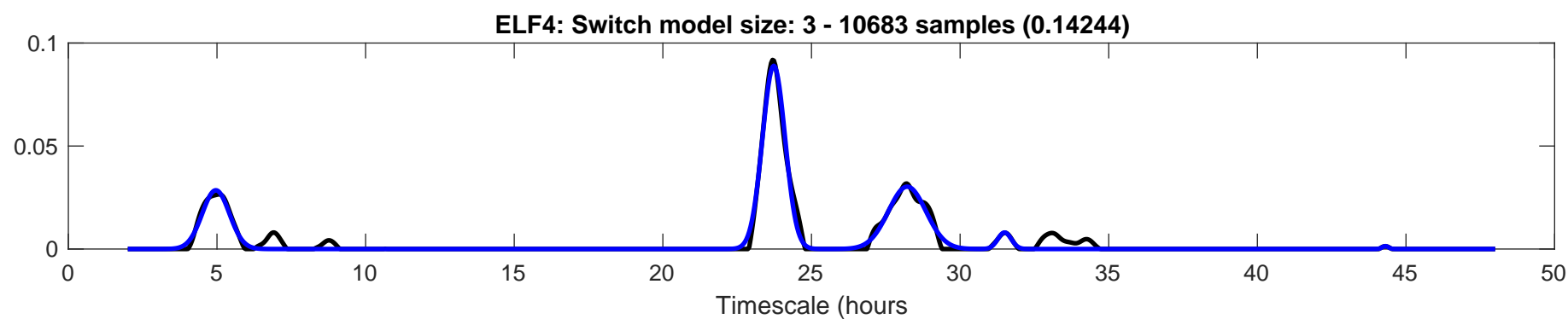

Supplement: Additional file 1 — Toolbox and data. The MATLAB code for running ReTrOS toolbox. This also includes the data used in the illustrative examples and the user manual that provides the instructions on how to run the toolbox. (ZIP 52838 kb) [file 12859_2017_1695_MOESM1_ESM.zip › ReTrOS-master/ReTrOSv5.4/output/clock_genes_mRNA/plots_switch/ELF4_models.pdf]

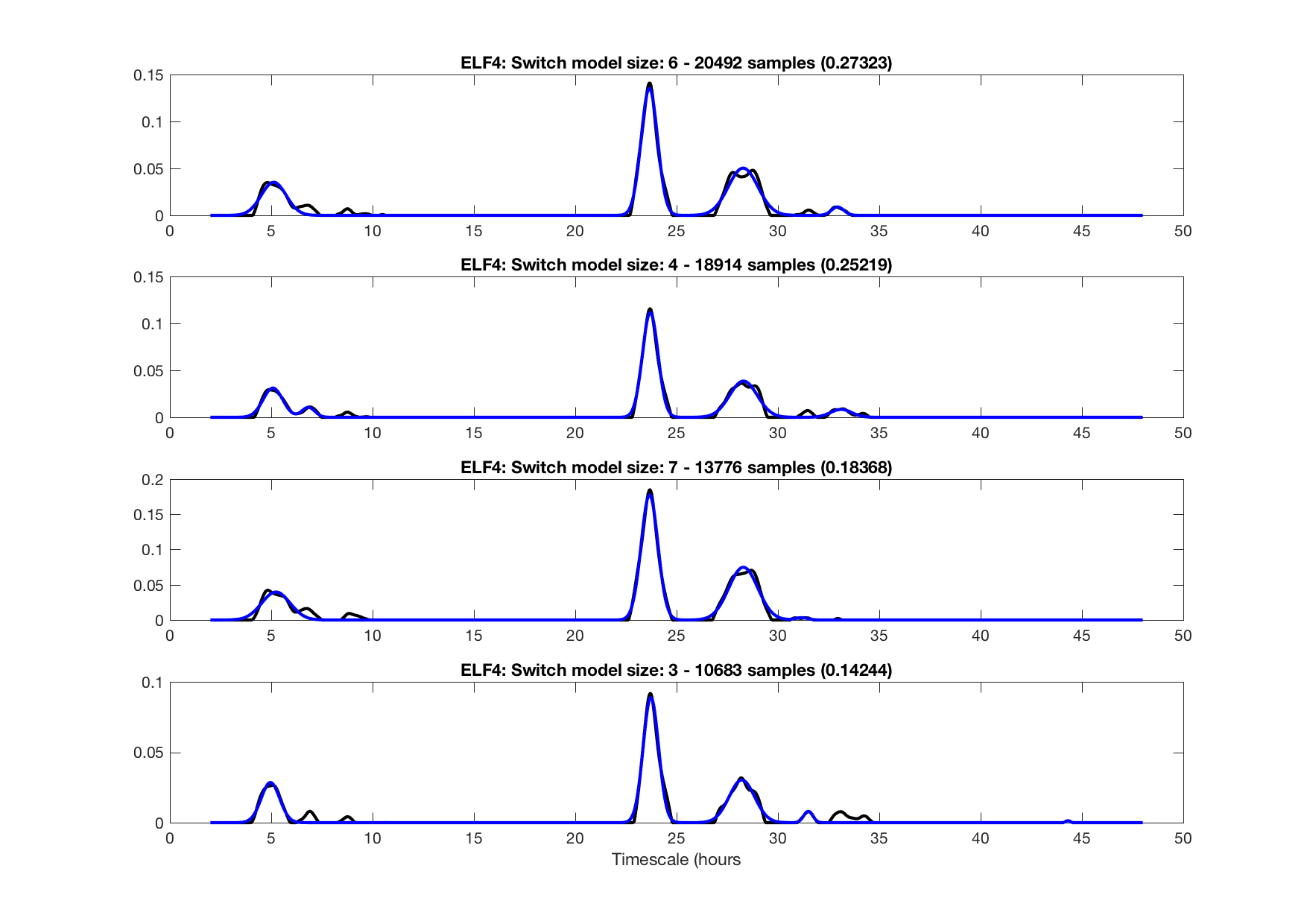

Supplement: Additional file 1 — Toolbox and data. The MATLAB code for running ReTrOS toolbox. This also includes the data used in the illustrative examples and the user manual that provides the instructions on how to run the toolbox. (ZIP 52838 kb) [file 12859_2017_1695_MOESM1_ESM.zip › ReTrOS-master/ReTrOSv5.4/output/clock_genes_mRNA/plots_switch/ELF4_models.png]

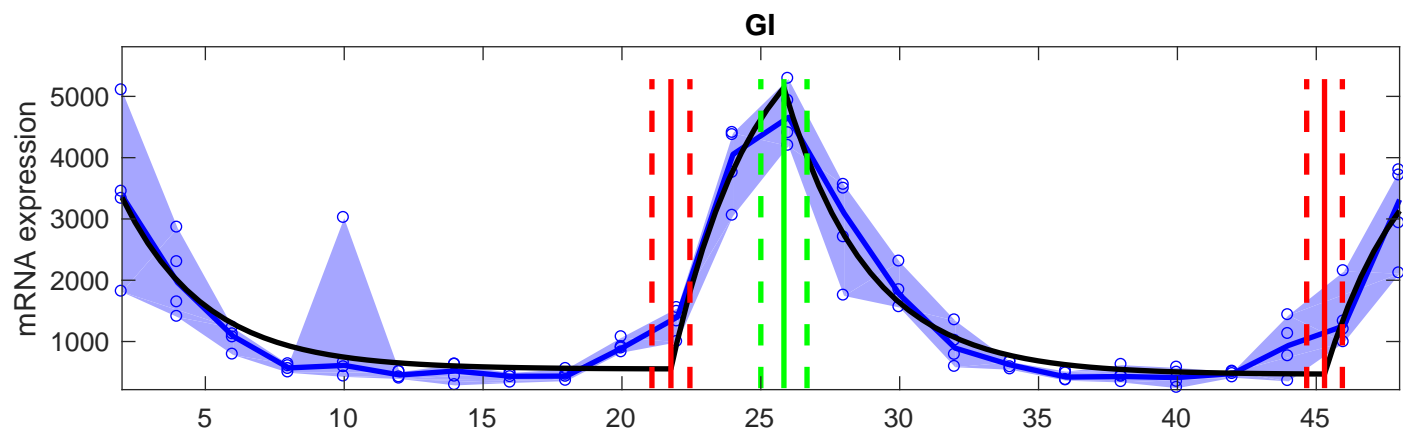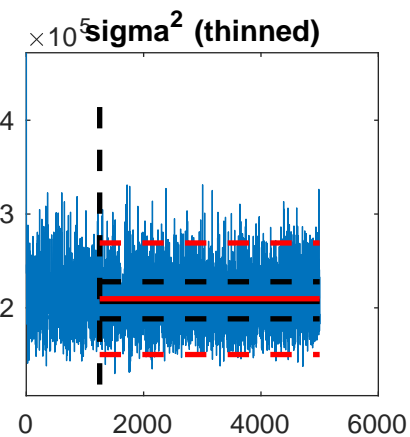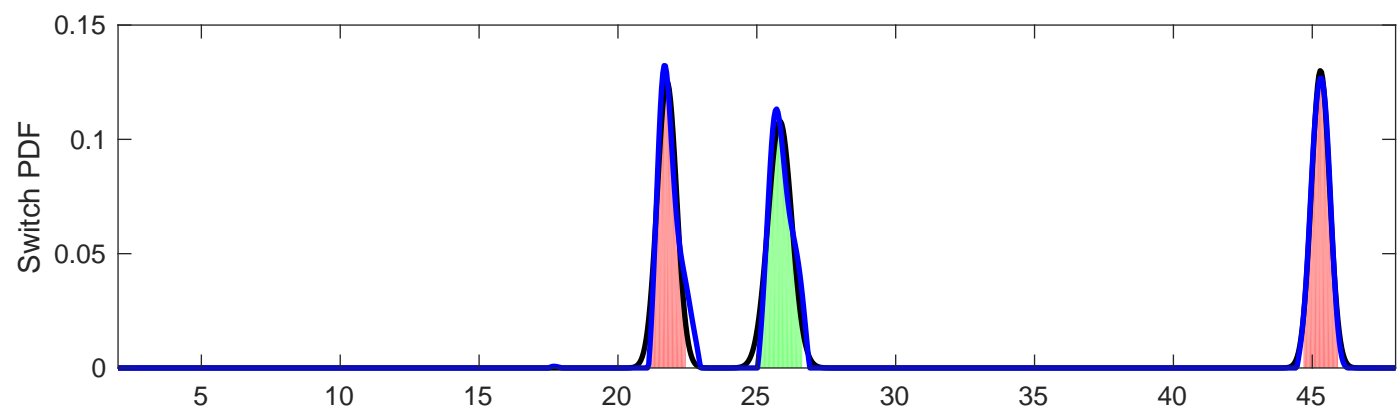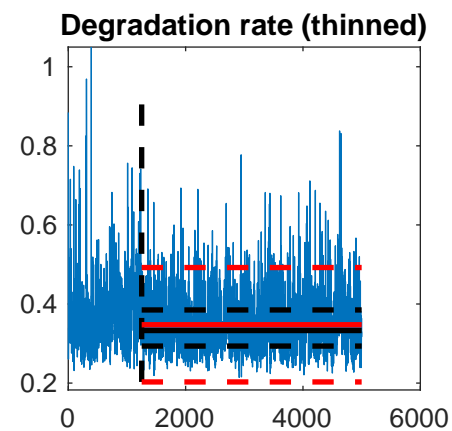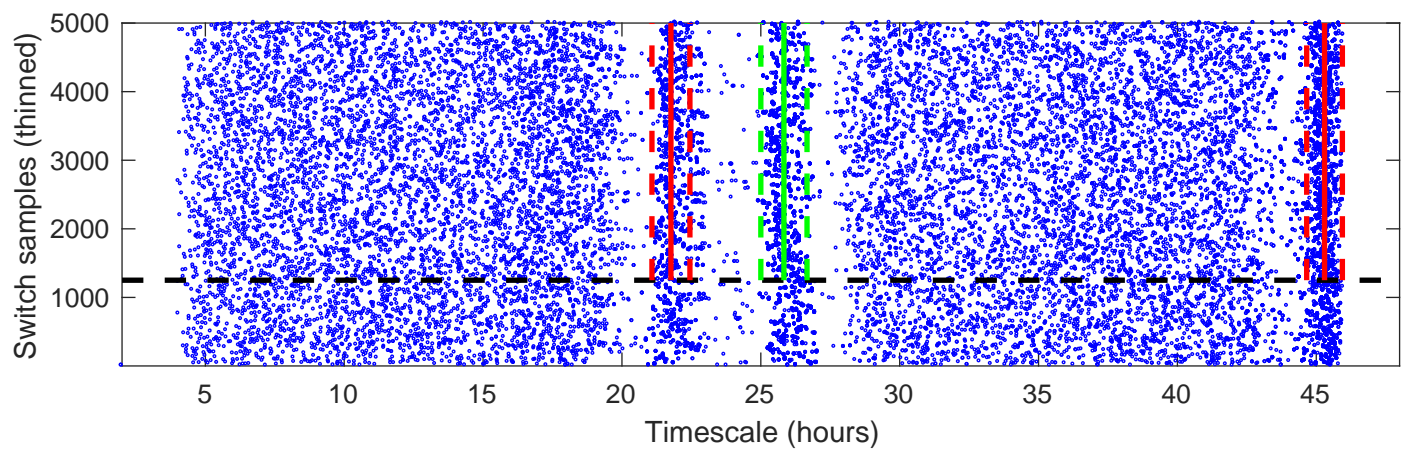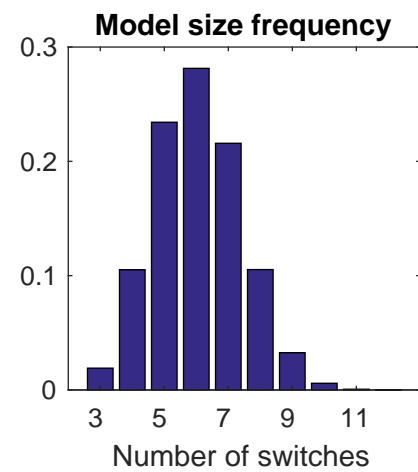

Supplement: Additional file 1 — Toolbox and data. The MATLAB code for running ReTrOS toolbox. This also includes the data used in the illustrative examples and the user manual that provides the instructions on how to run the toolbox. (ZIP 52838 kb) [file 12859_2017_1695_MOESM1_ESM.zip › ReTrOS-master/ReTrOSv5.4/output/clock_genes_mRNA/plots_switch/GI.pdf]

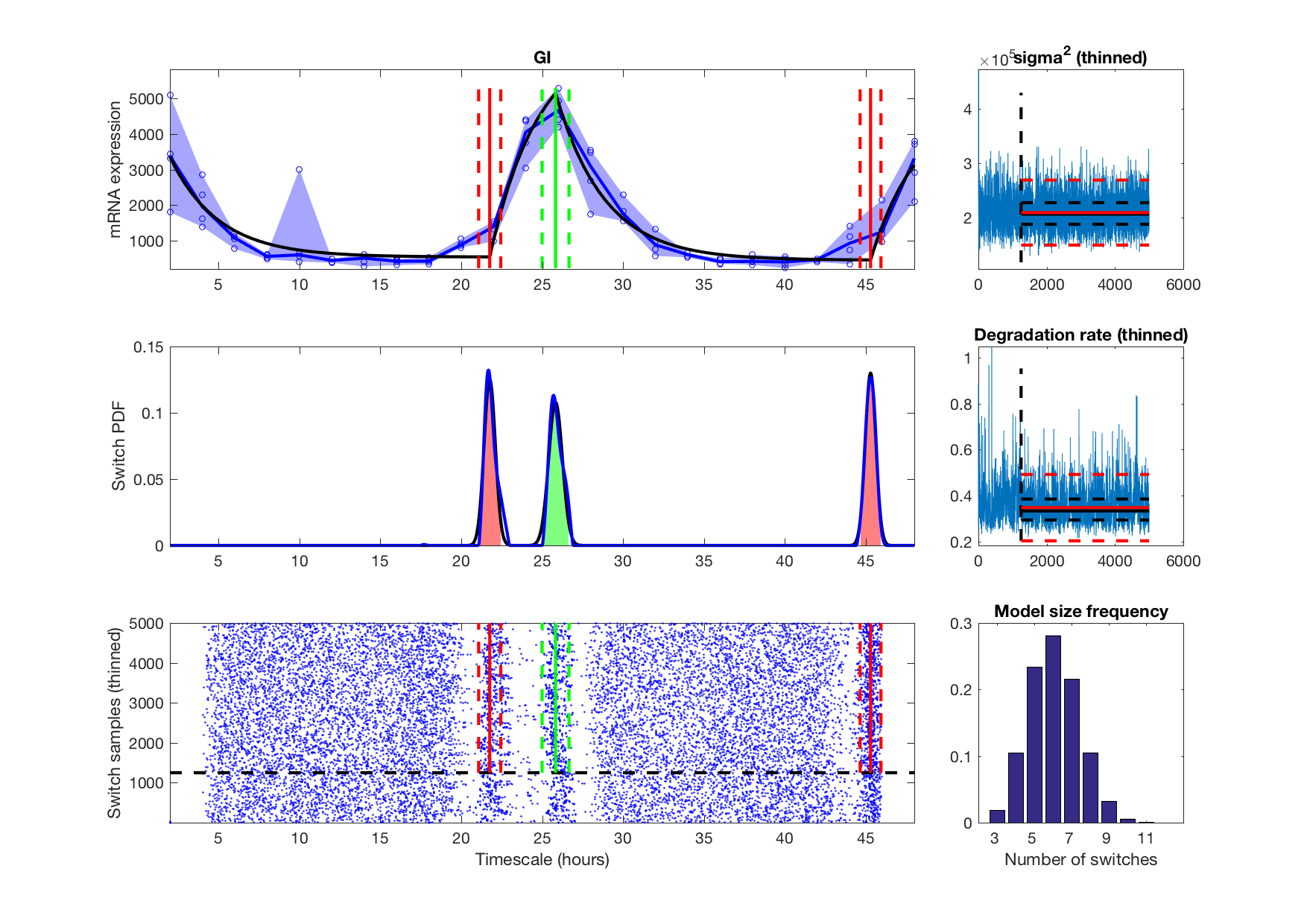

Supplement: Additional file 1 — Toolbox and data. The MATLAB code for running ReTrOS toolbox. This also includes the data used in the illustrative examples and the user manual that provides the instructions on how to run the toolbox. (ZIP 52838 kb) [file 12859_2017_1695_MOESM1_ESM.zip › ReTrOS-master/ReTrOSv5.4/output/clock_genes_mRNA/plots_switch/GI.png]

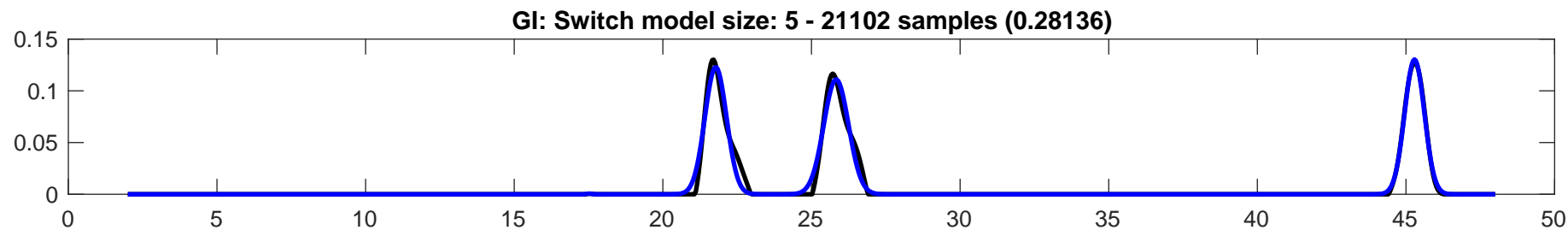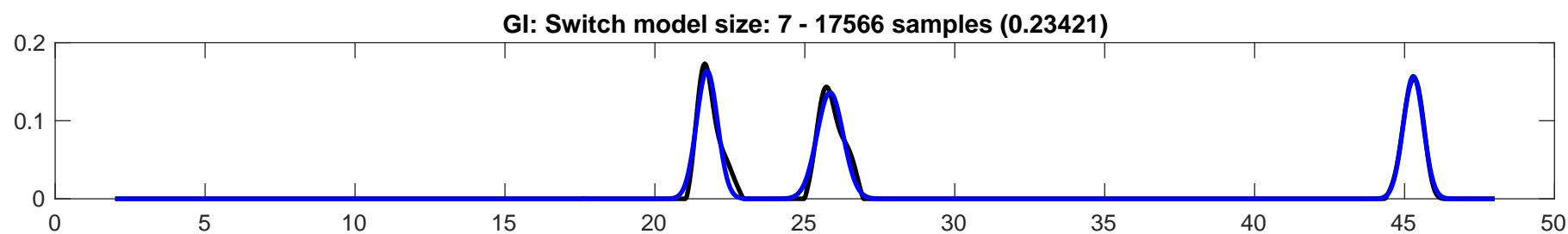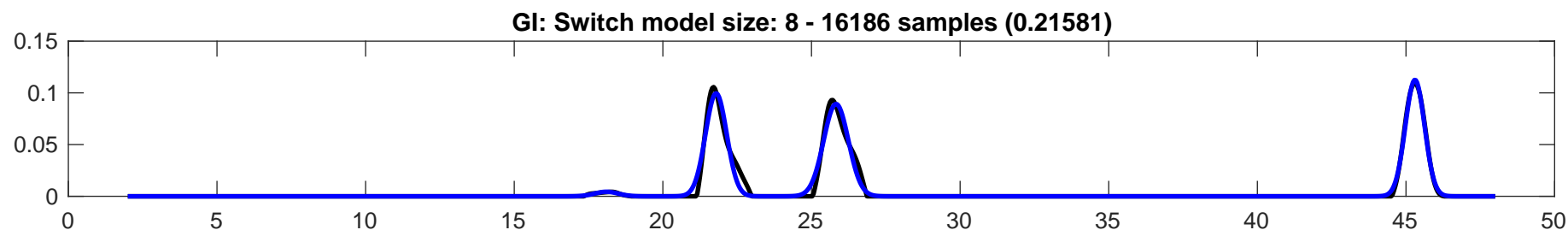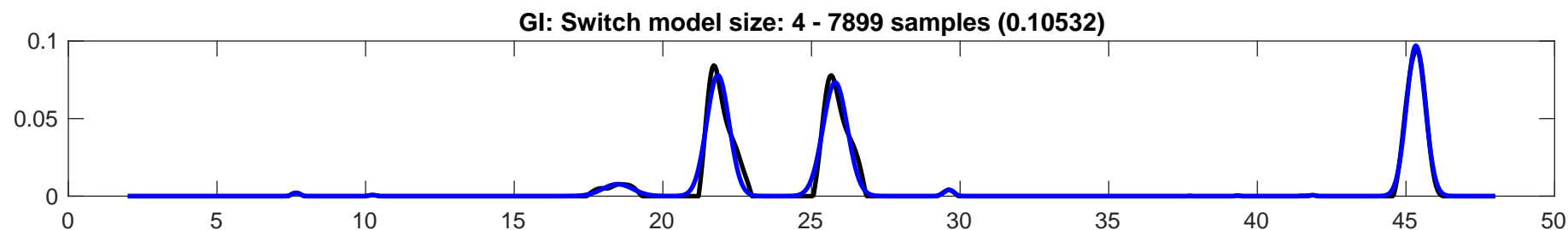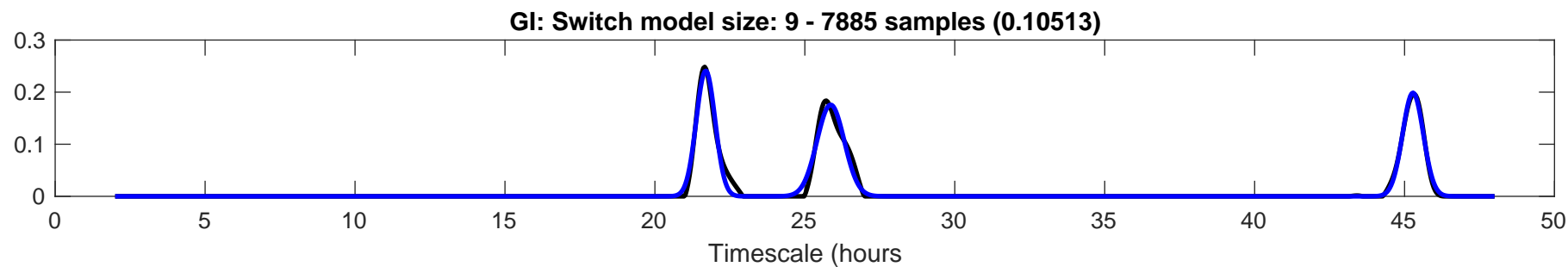

Supplement: Additional file 1 — Toolbox and data. The MATLAB code for running ReTrOS toolbox. This also includes the data used in the illustrative examples and the user manual that provides the instructions on how to run the toolbox. (ZIP 52838 kb) [file 12859_2017_1695_MOESM1_ESM.zip › ReTrOS-master/ReTrOSv5.4/output/clock_genes_mRNA/plots_switch/GI_models.pdf]

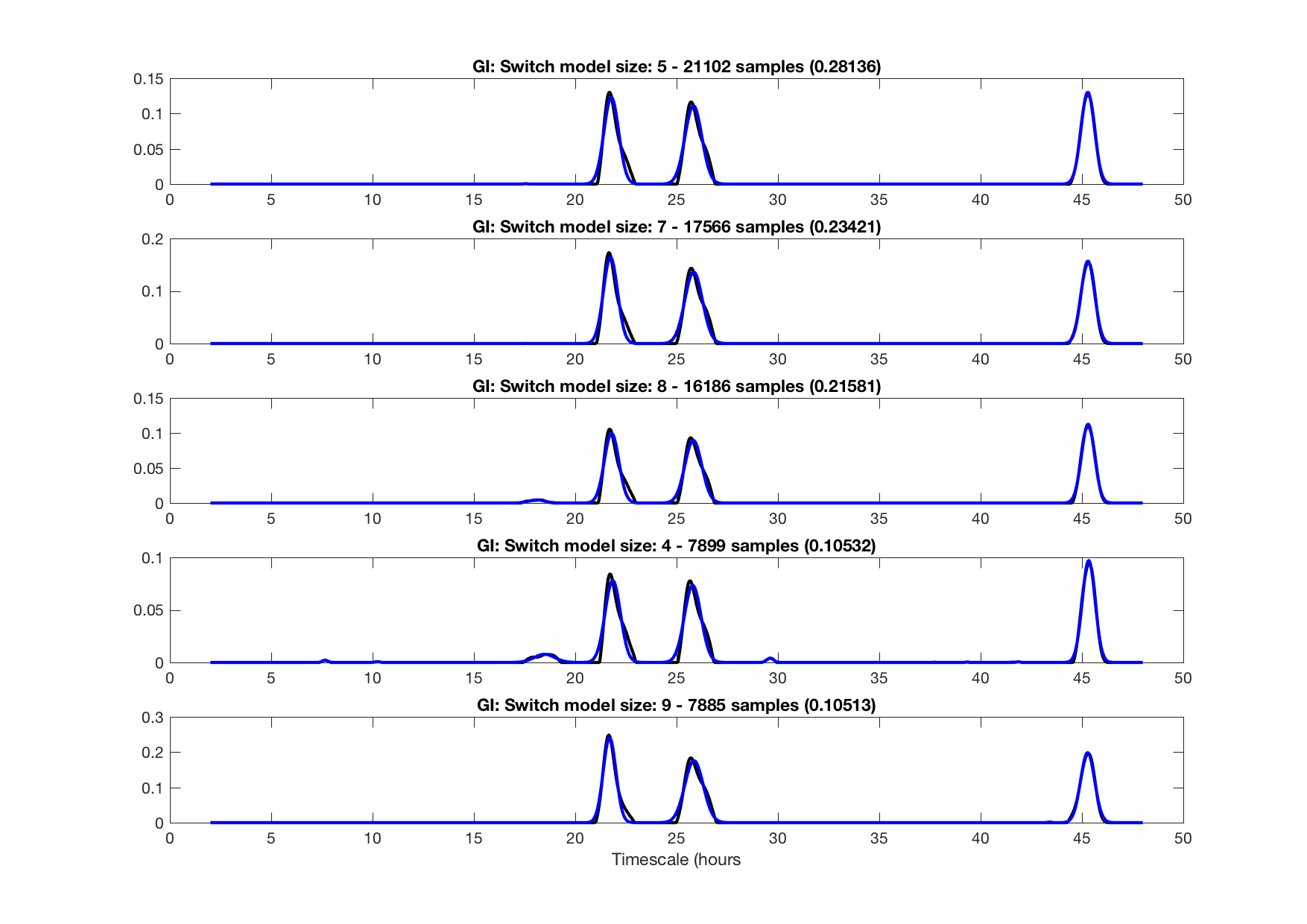

Supplement: Additional file 1 — Toolbox and data. The MATLAB code for running ReTrOS toolbox. This also includes the data used in the illustrative examples and the user manual that provides the instructions on how to run the toolbox. (ZIP 52838 kb) [file 12859_2017_1695_MOESM1_ESM.zip › ReTrOS-master/ReTrOSv5.4/output/clock_genes_mRNA/plots_switch/GI_models.png]

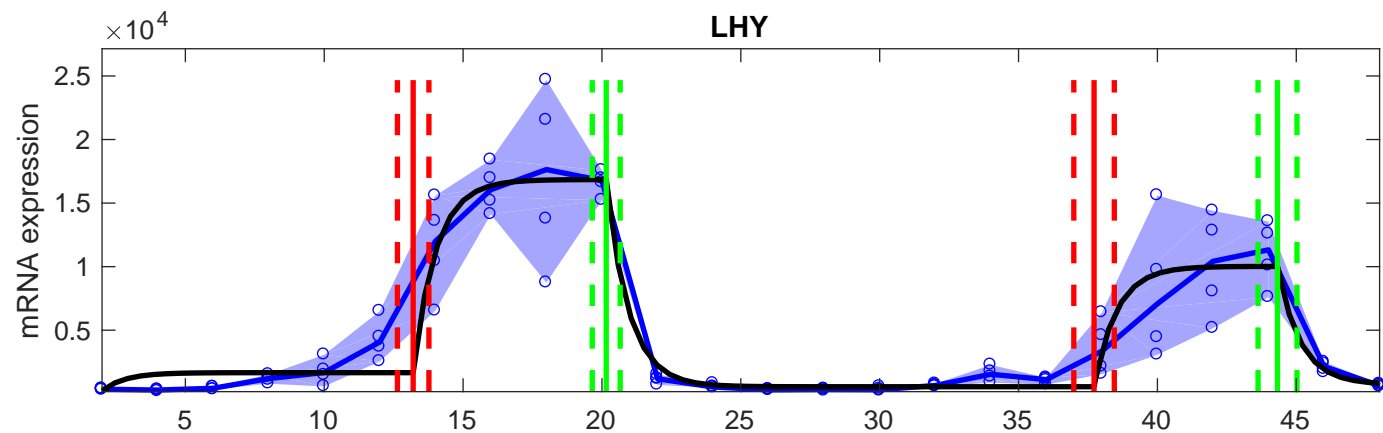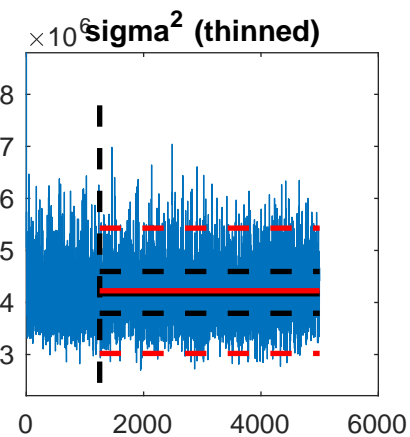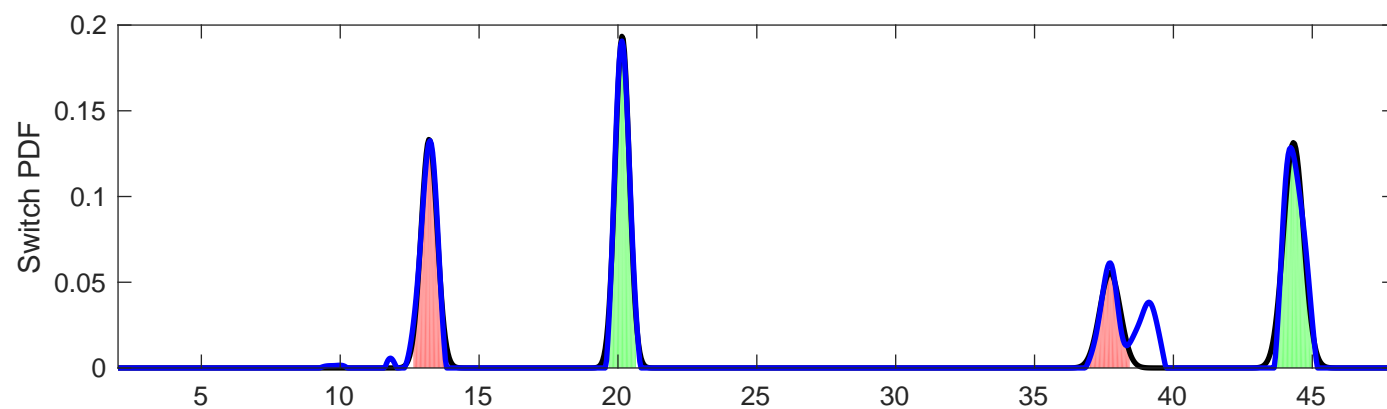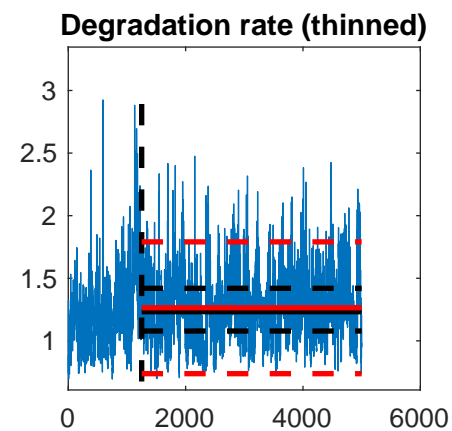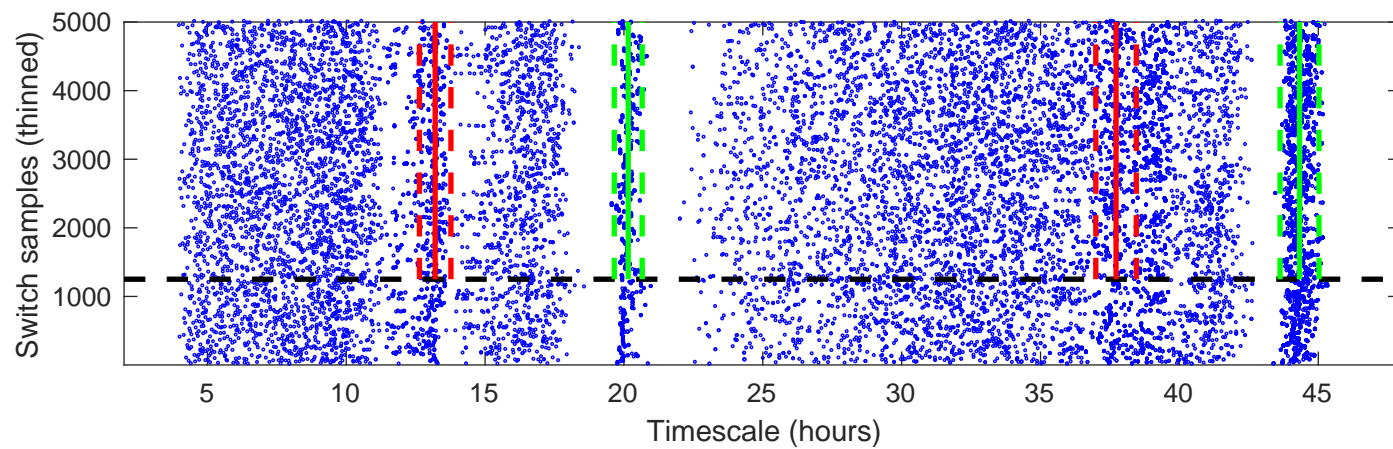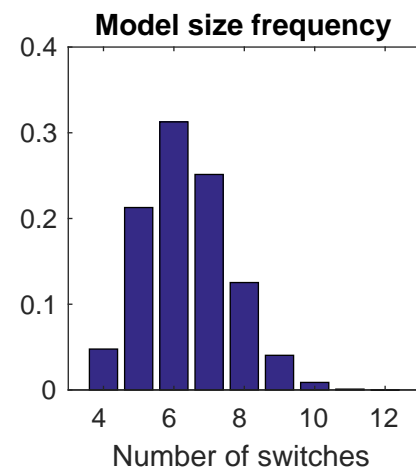

Supplement: Additional file 1 — Toolbox and data. The MATLAB code for running ReTrOS toolbox. This also includes the data used in the illustrative examples and the user manual that provides the instructions on how to run the toolbox. (ZIP 52838 kb) [file 12859_2017_1695_MOESM1_ESM.zip › ReTrOS-master/ReTrOSv5.4/output/clock_genes_mRNA/plots_switch/LHY.pdf]

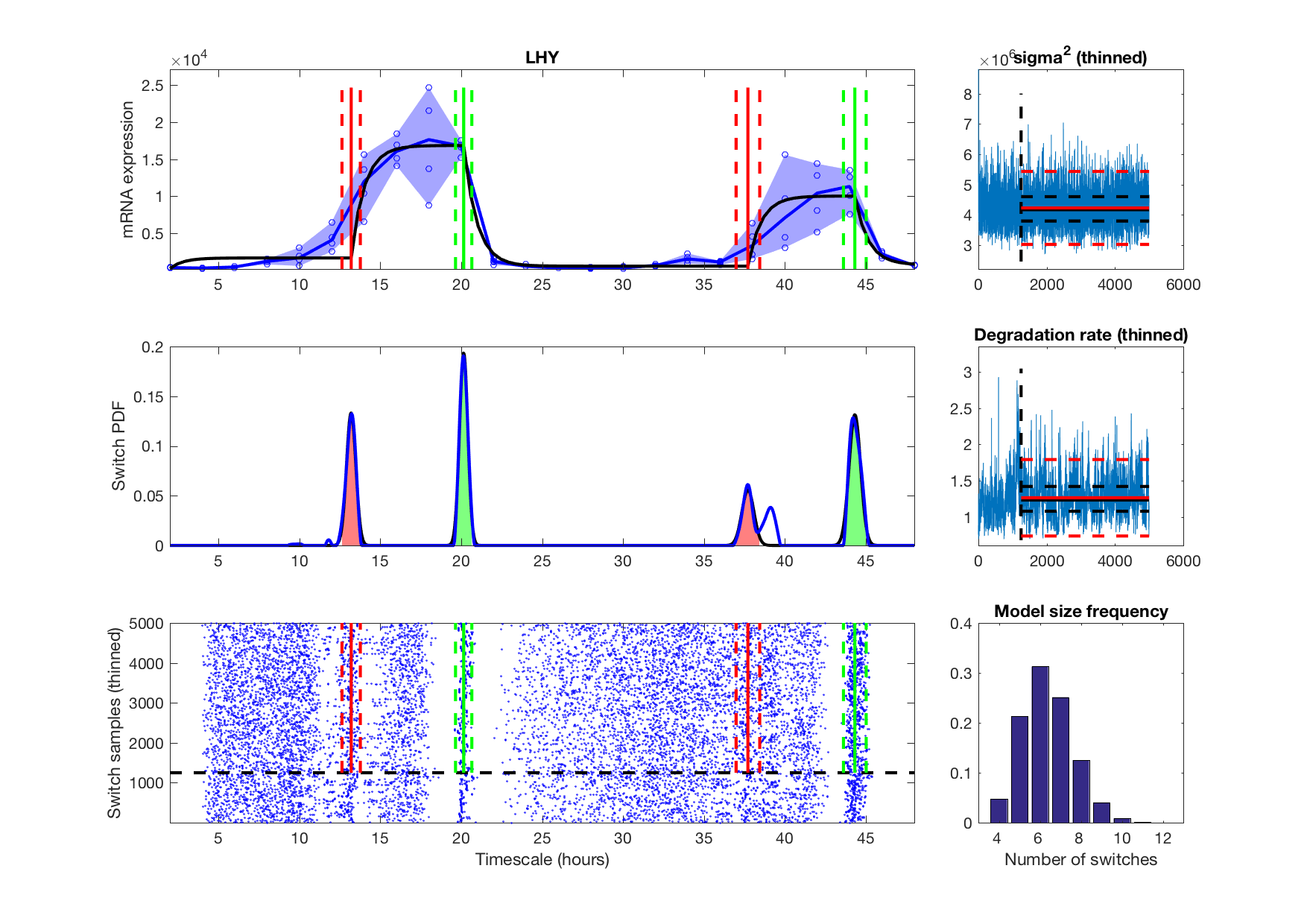

Supplement: Additional file 1 — Toolbox and data. The MATLAB code for running ReTrOS toolbox. This also includes the data used in the illustrative examples and the user manual that provides the instructions on how to run the toolbox. (ZIP 52838 kb) [file 12859_2017_1695_MOESM1_ESM.zip › ReTrOS-master/ReTrOSv5.4/output/clock_genes_mRNA/plots_switch/LHY.png]

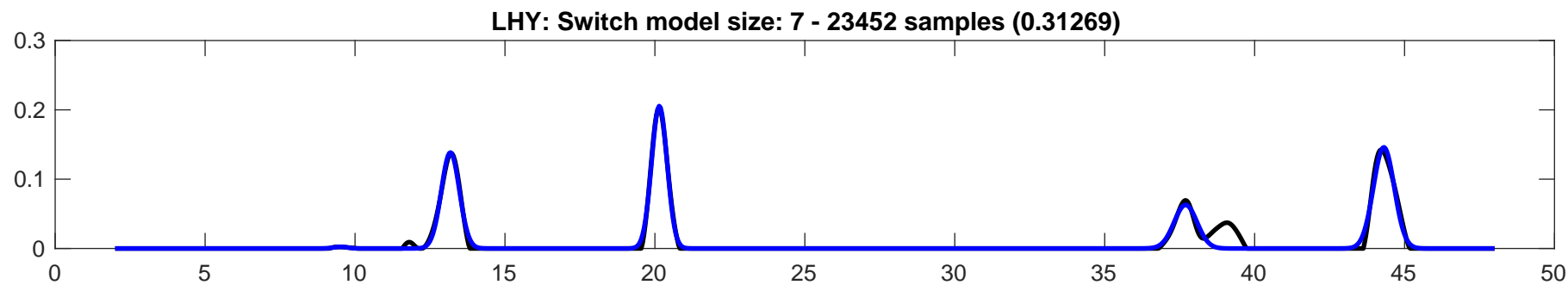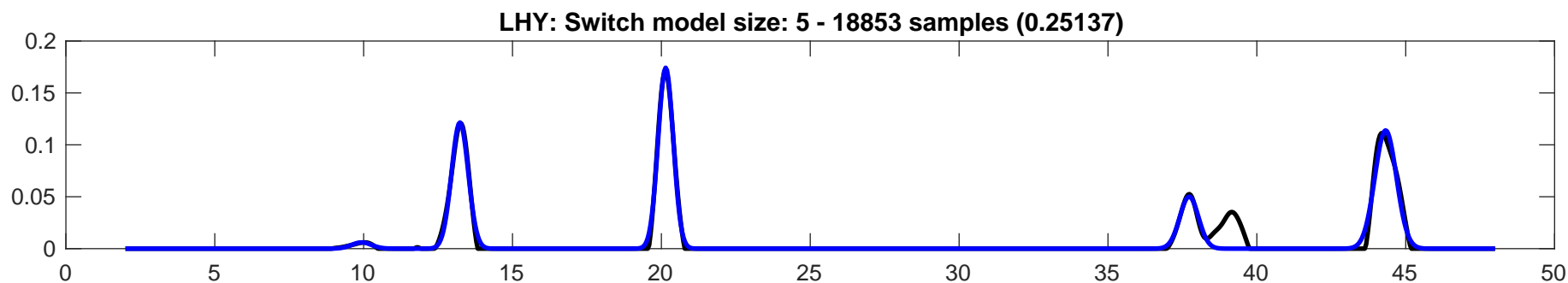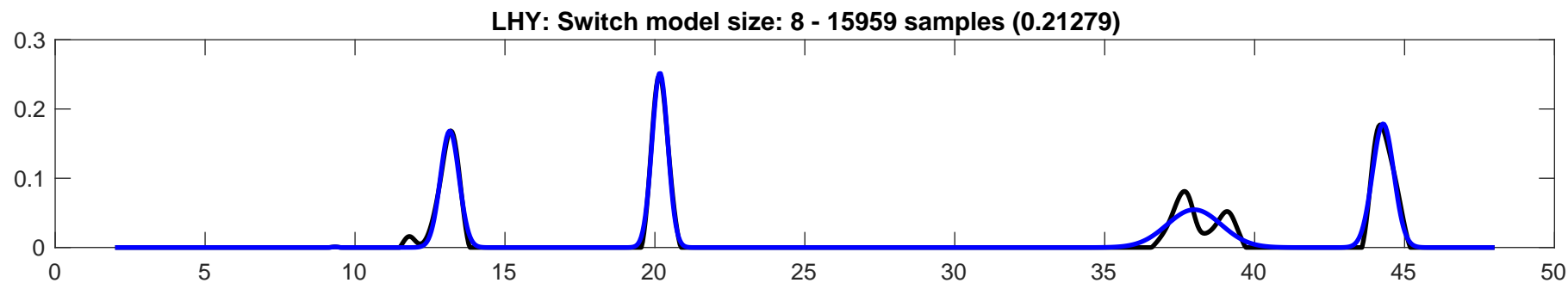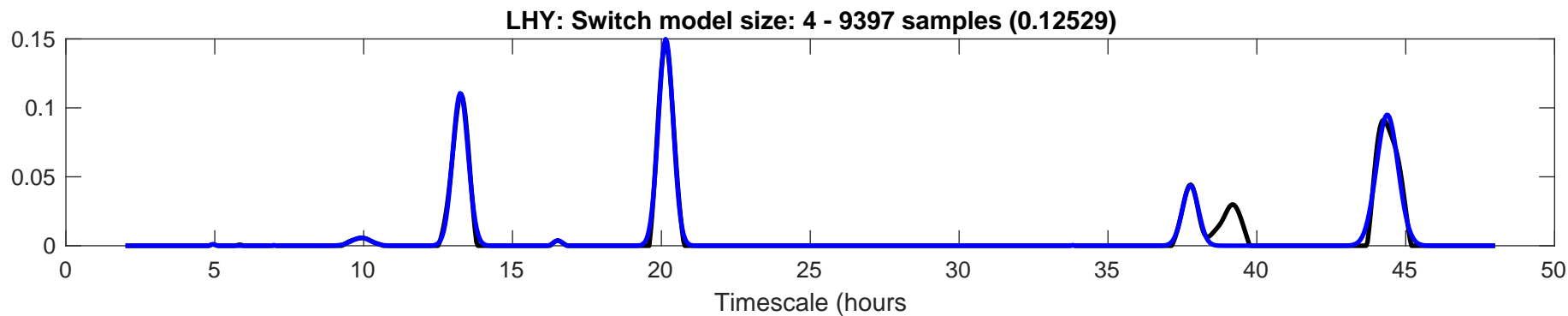

Supplement: Additional file 1 — Toolbox and data. The MATLAB code for running ReTrOS toolbox. This also includes the data used in the illustrative examples and the user manual that provides the instructions on how to run the toolbox. (ZIP 52838 kb) [file 12859_2017_1695_MOESM1_ESM.zip › ReTrOS-master/ReTrOSv5.4/output/clock_genes_mRNA/plots_switch/LHY_models.pdf]

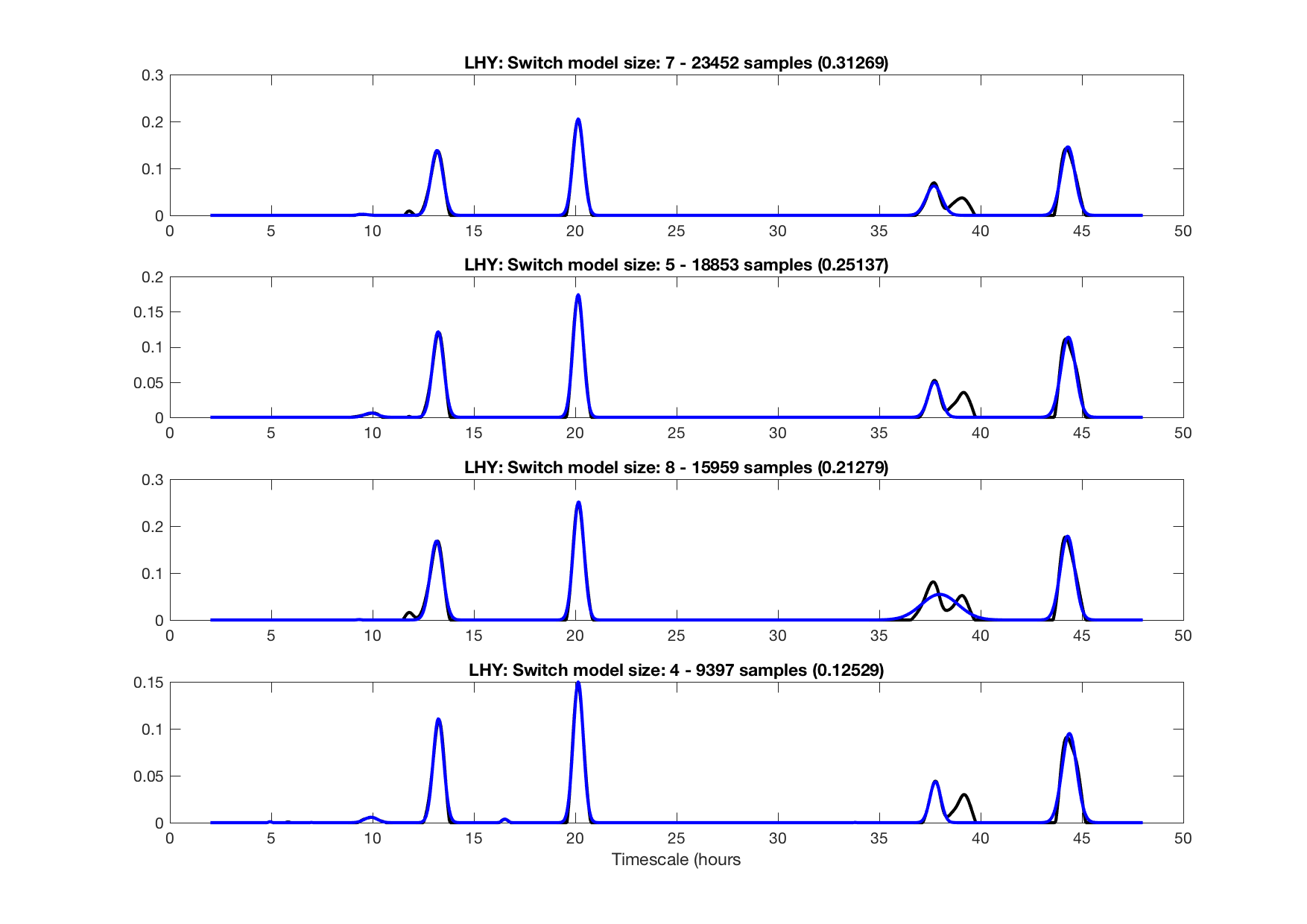

Supplement: Additional file 1 — Toolbox and data. The MATLAB code for running ReTrOS toolbox. This also includes the data used in the illustrative examples and the user manual that provides the instructions on how to run the toolbox. (ZIP 52838 kb) [file 12859_2017_1695_MOESM1_ESM.zip › ReTrOS-master/ReTrOSv5.4/output/clock_genes_mRNA/plots_switch/LHY_models.png]

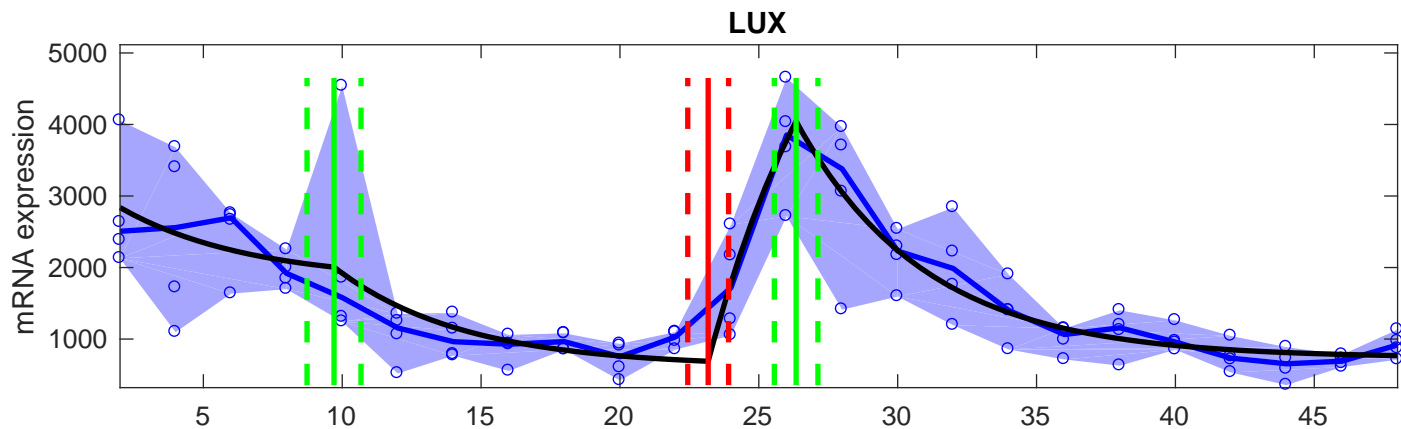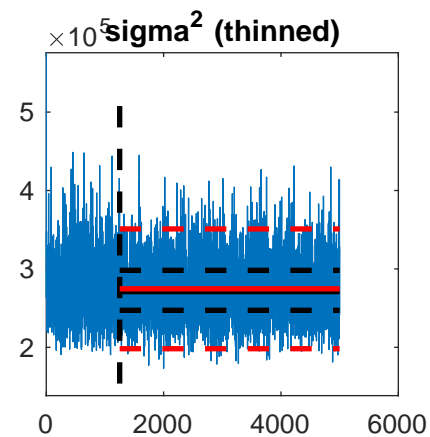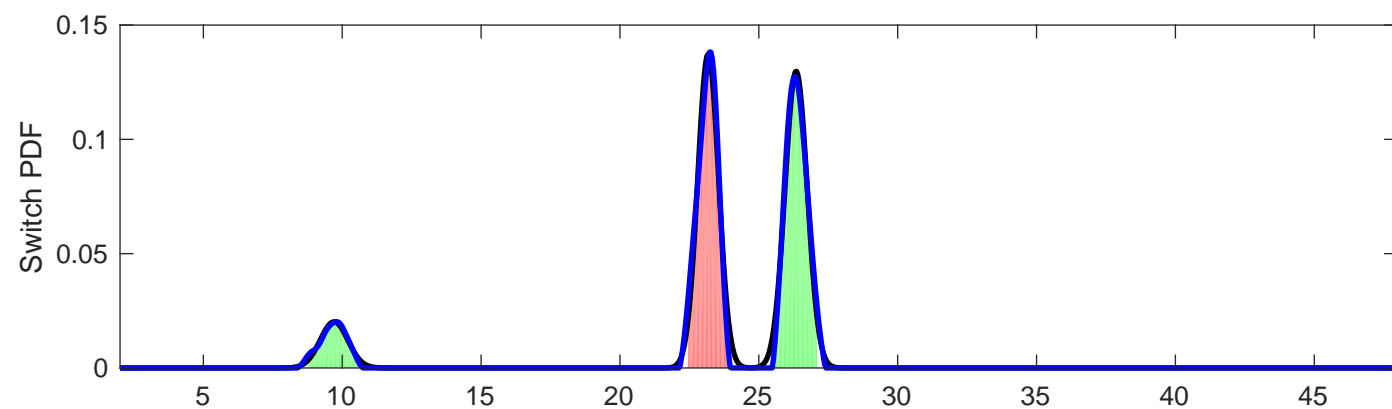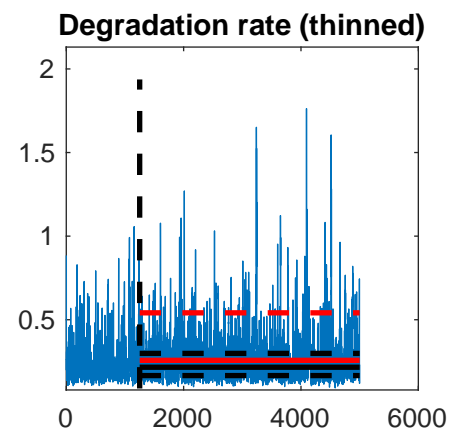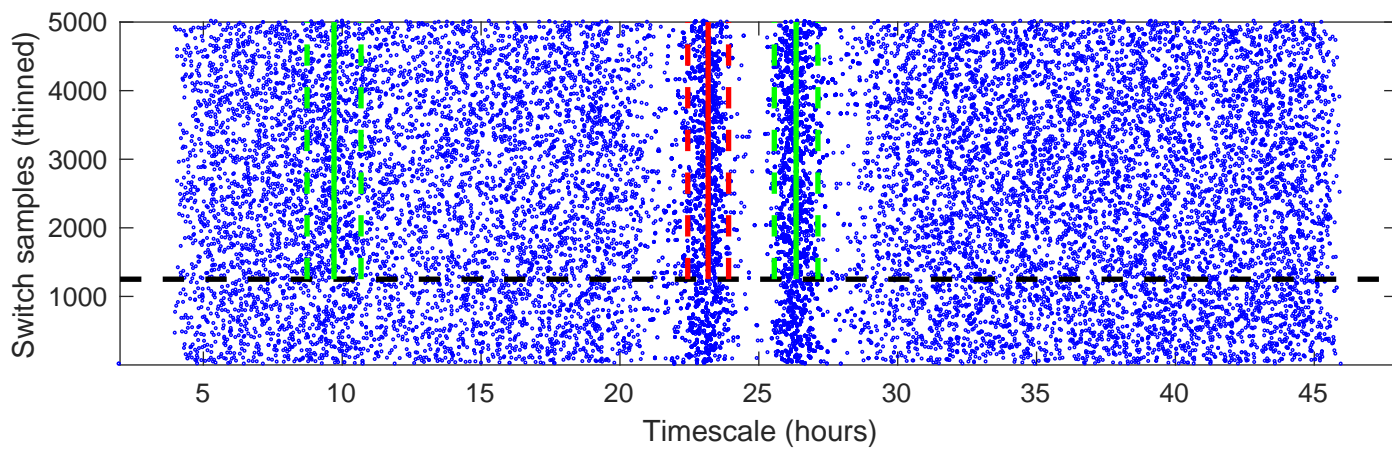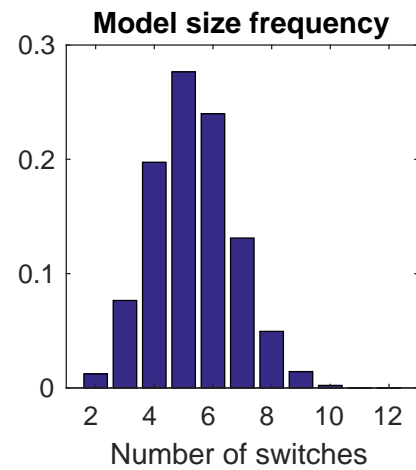

Supplement: Additional file 1 — Toolbox and data. The MATLAB code for running ReTrOS toolbox. This also includes the data used in the illustrative examples and the user manual that provides the instructions on how to run the toolbox. (ZIP 52838 kb) [file 12859_2017_1695_MOESM1_ESM.zip › ReTrOS-master/ReTrOSv5.4/output/clock_genes_mRNA/plots_switch/LUX.pdf]

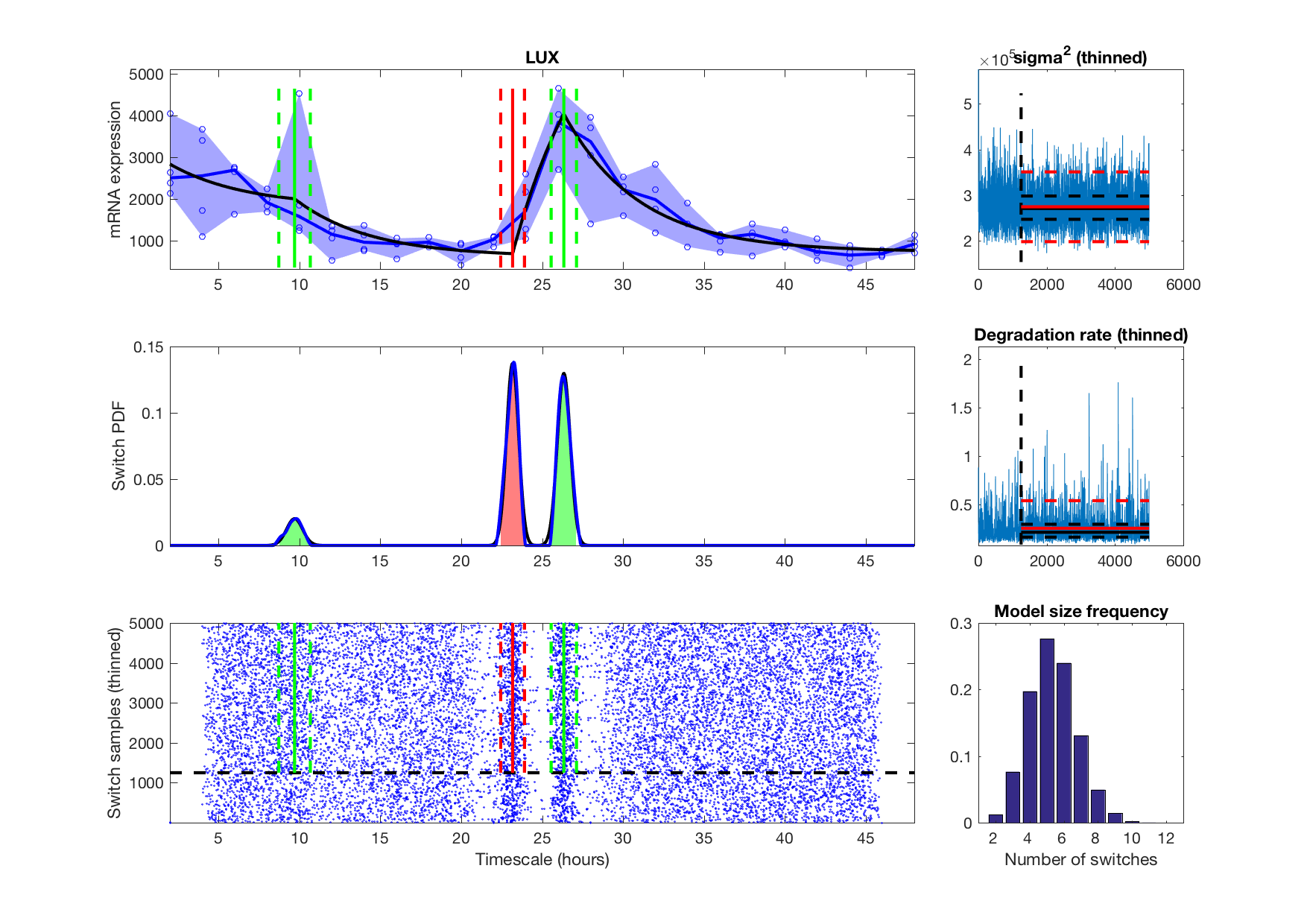

Supplement: Additional file 1 — Toolbox and data. The MATLAB code for running ReTrOS toolbox. This also includes the data used in the illustrative examples and the user manual that provides the instructions on how to run the toolbox. (ZIP 52838 kb) [file 12859_2017_1695_MOESM1_ESM.zip › ReTrOS-master/ReTrOSv5.4/output/clock_genes_mRNA/plots_switch/LUX.png]

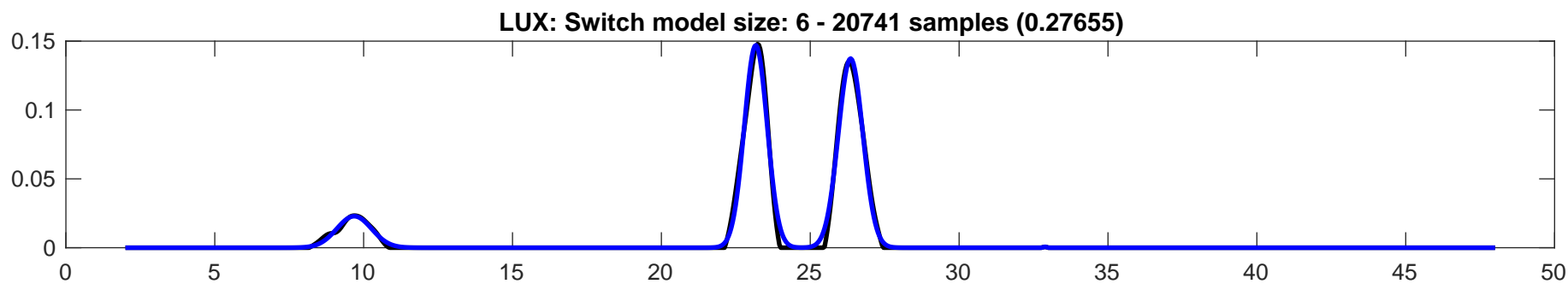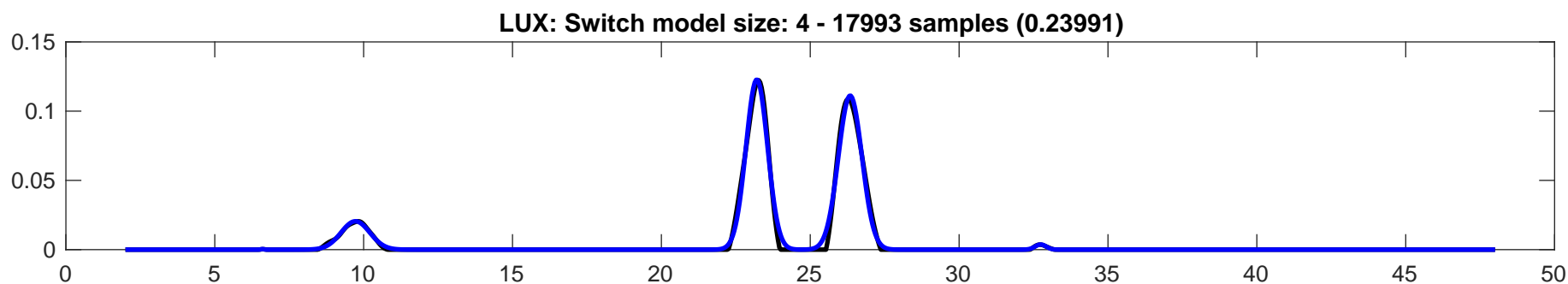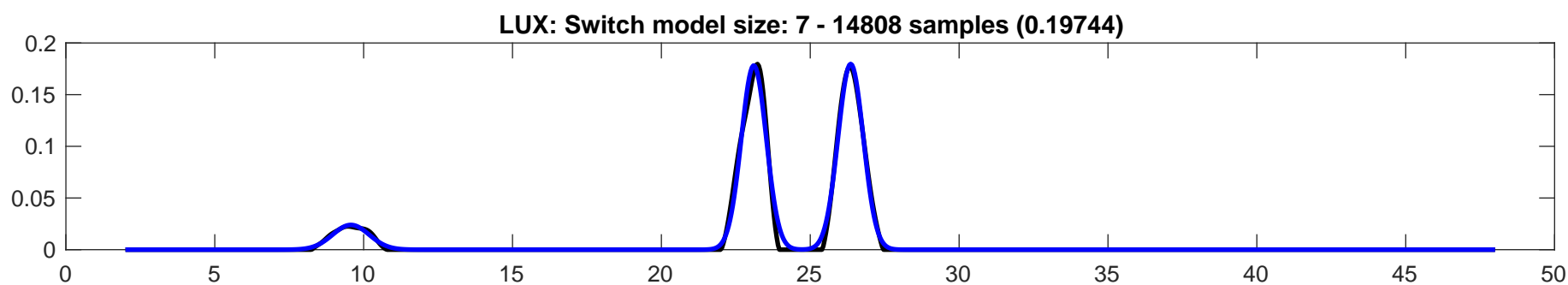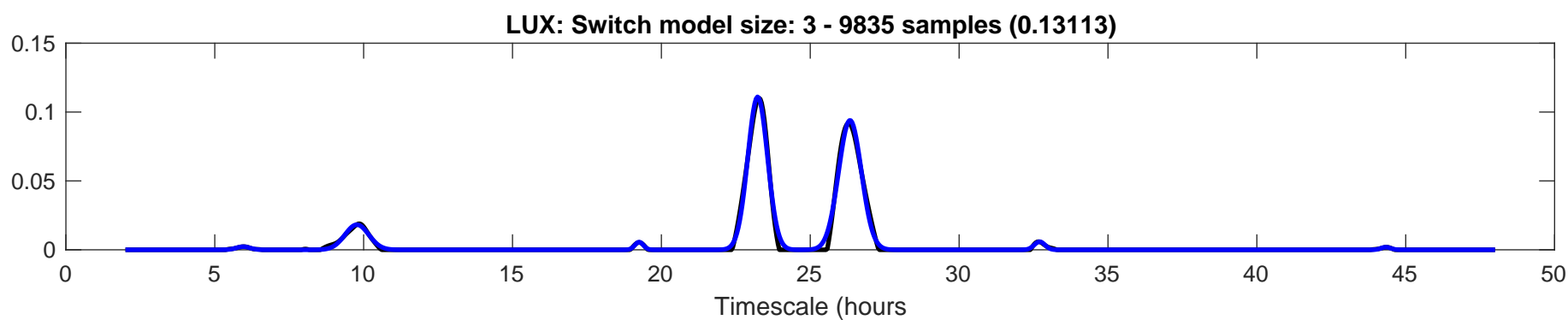

Supplement: Additional file 1 — Toolbox and data. The MATLAB code for running ReTrOS toolbox. This also includes the data used in the illustrative examples and the user manual that provides the instructions on how to run the toolbox. (ZIP 52838 kb) [file 12859_2017_1695_MOESM1_ESM.zip › ReTrOS-master/ReTrOSv5.4/output/clock_genes_mRNA/plots_switch/LUX_models.pdf]

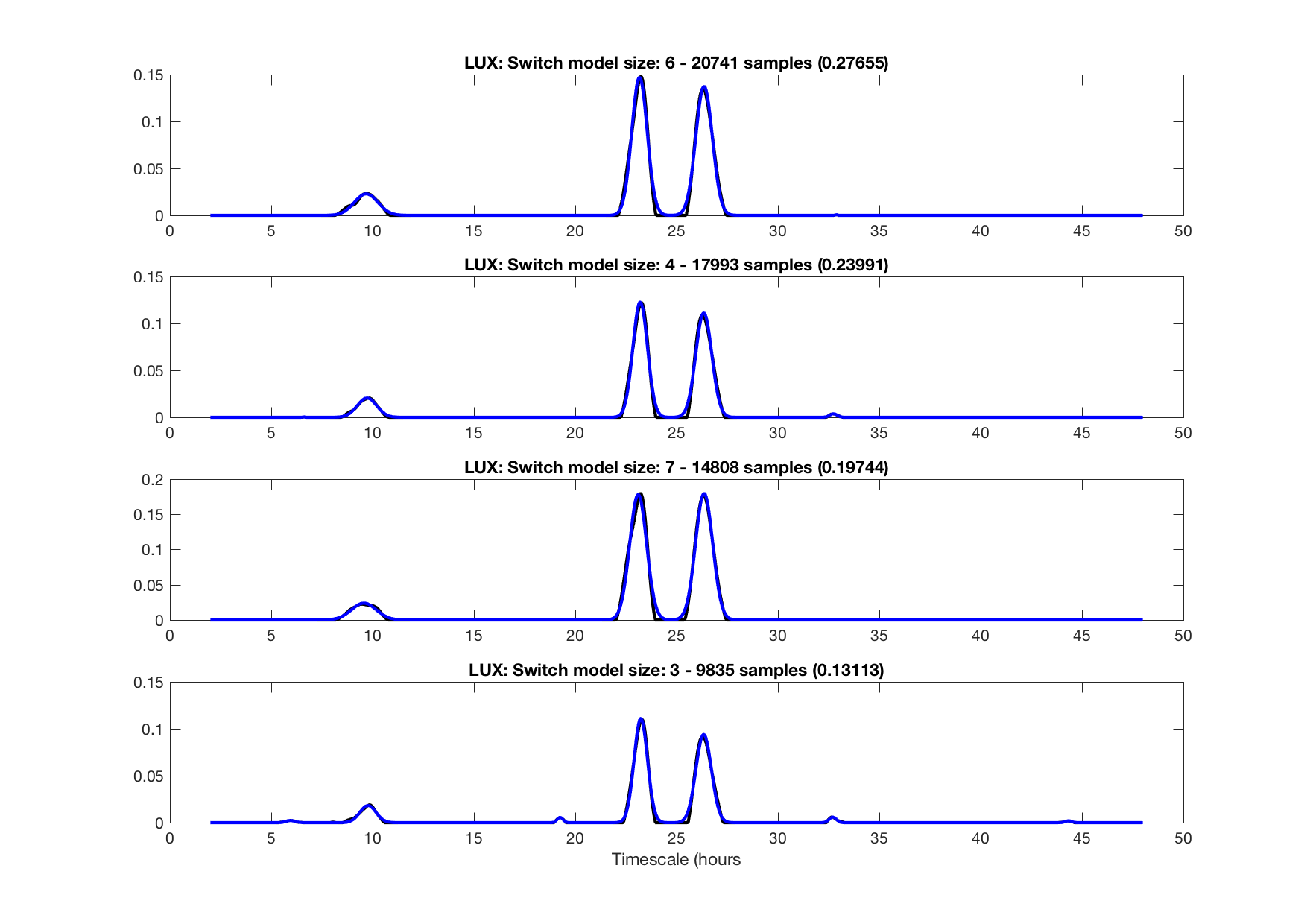

Supplement: Additional file 1 — Toolbox and data. The MATLAB code for running ReTrOS toolbox. This also includes the data used in the illustrative examples and the user manual that provides the instructions on how to run the toolbox. (ZIP 52838 kb) [file 12859_2017_1695_MOESM1_ESM.zip › ReTrOS-master/ReTrOSv5.4/output/clock_genes_mRNA/plots_switch/LUX_models.png]

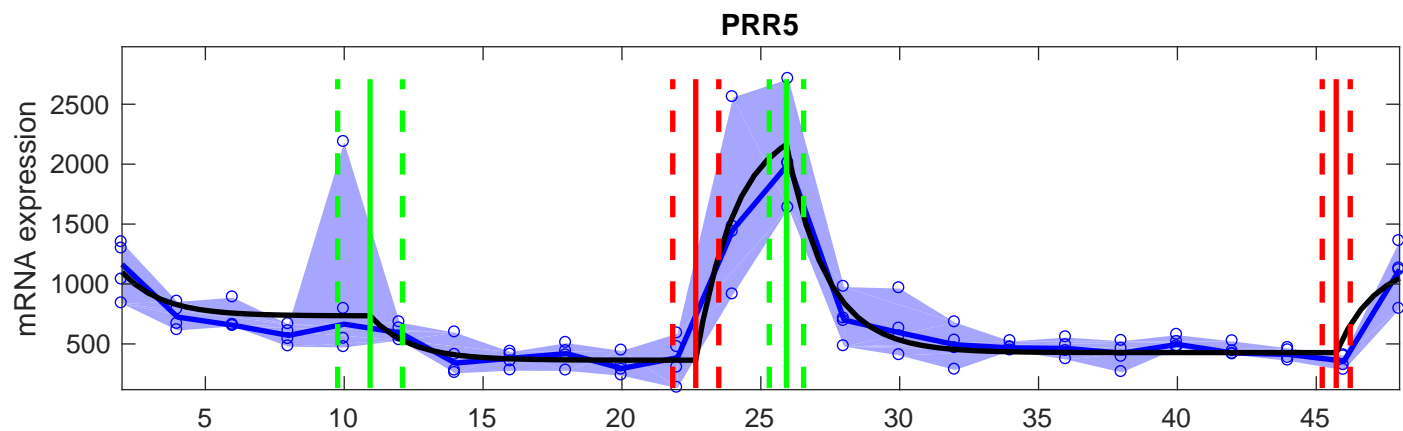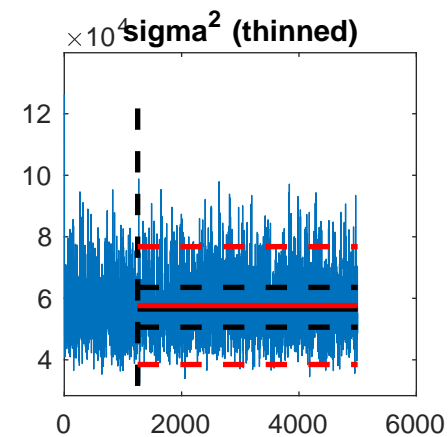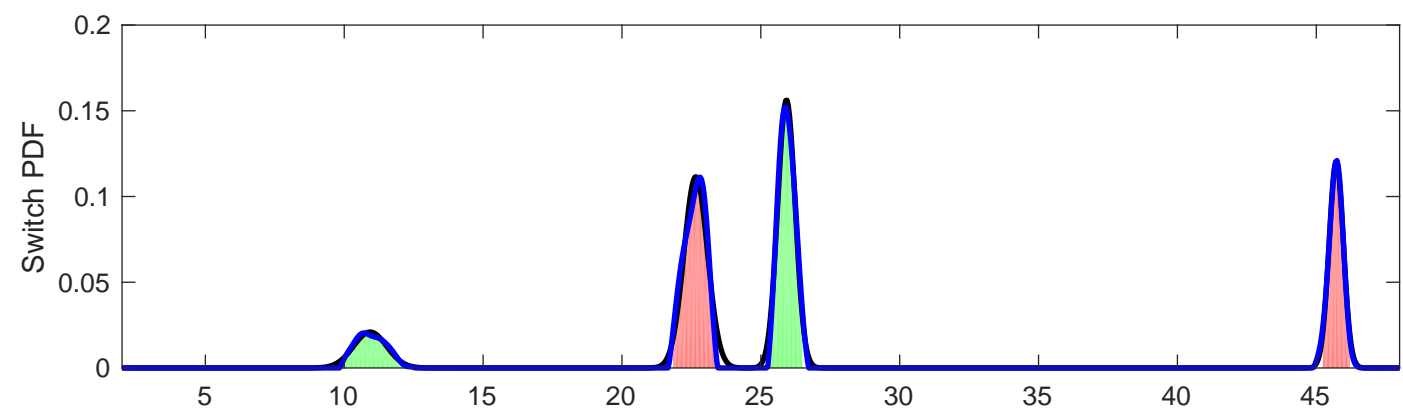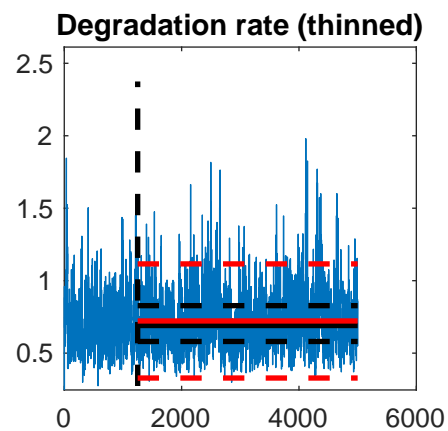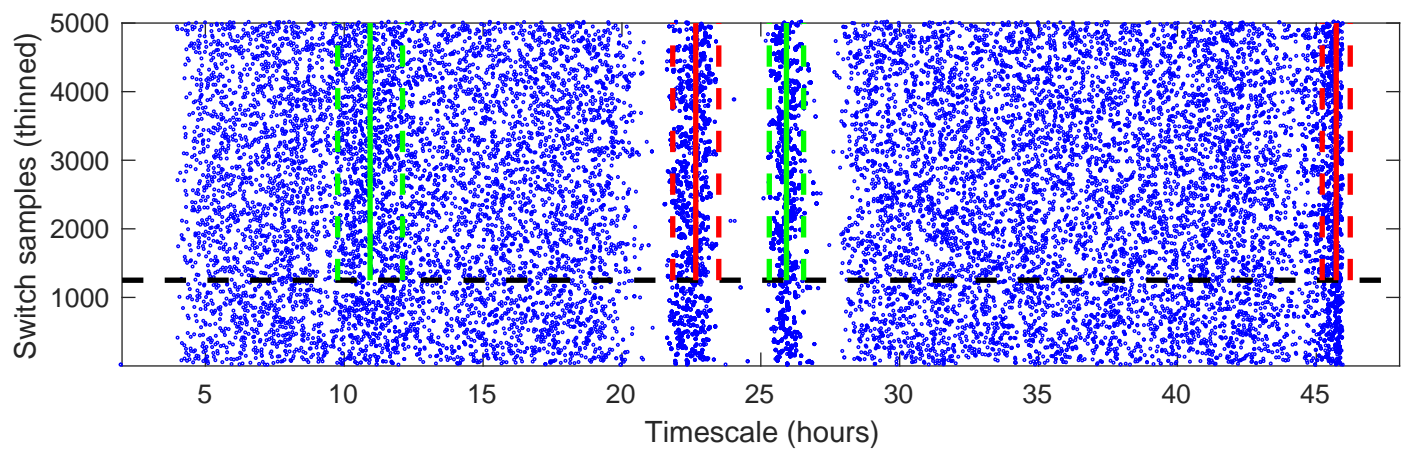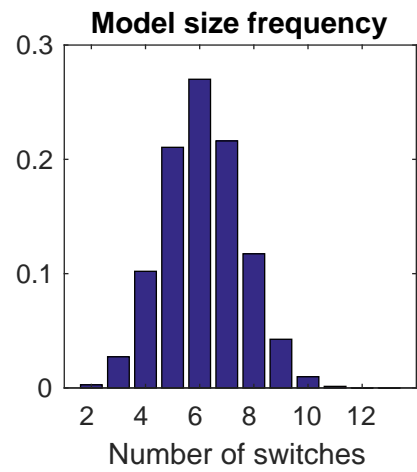

Supplement: Additional file 1 — Toolbox and data. The MATLAB code for running ReTrOS toolbox. This also includes the data used in the illustrative examples and the user manual that provides the instructions on how to run the toolbox. (ZIP 52838 kb) [file 12859_2017_1695_MOESM1_ESM.zip › ReTrOS-master/ReTrOSv5.4/output/clock_genes_mRNA/plots_switch/PRR5.pdf]

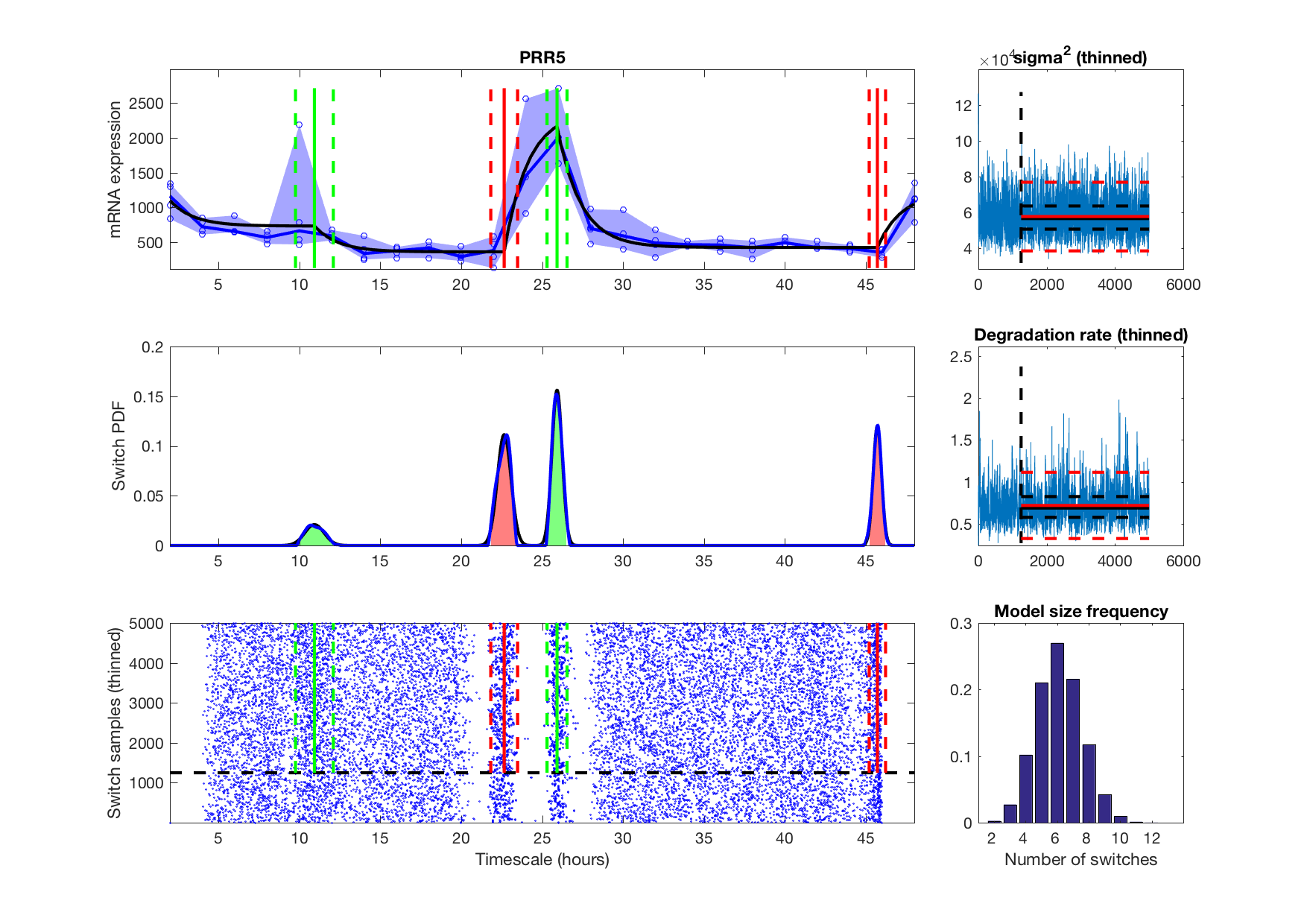

Supplement: Additional file 1 — Toolbox and data. The MATLAB code for running ReTrOS toolbox. This also includes the data used in the illustrative examples and the user manual that provides the instructions on how to run the toolbox. (ZIP 52838 kb) [file 12859_2017_1695_MOESM1_ESM.zip › ReTrOS-master/ReTrOSv5.4/output/clock_genes_mRNA/plots_switch/PRR5.png]

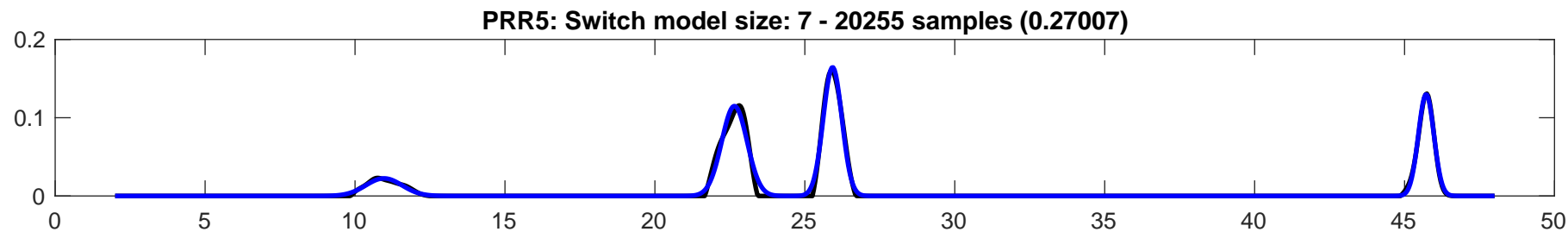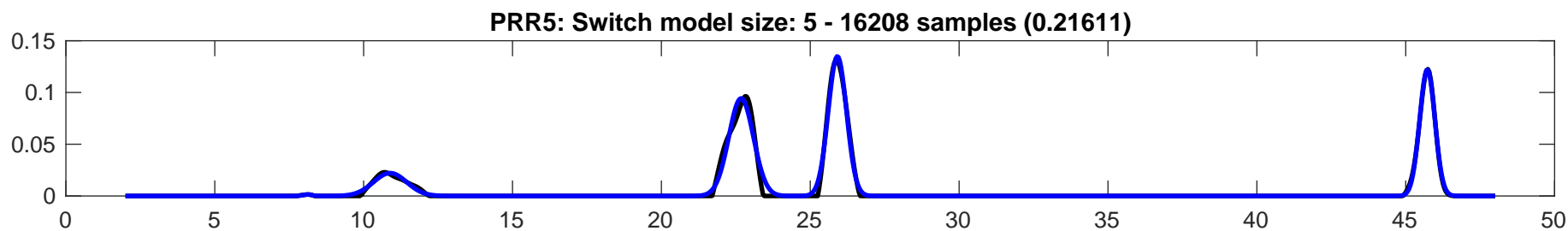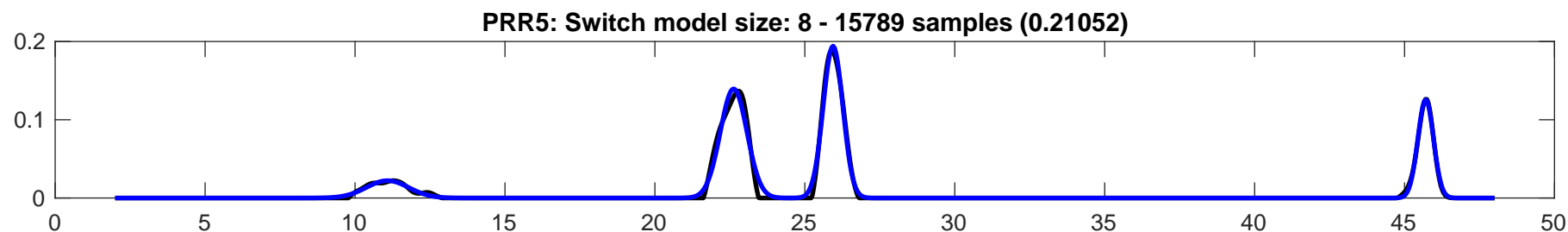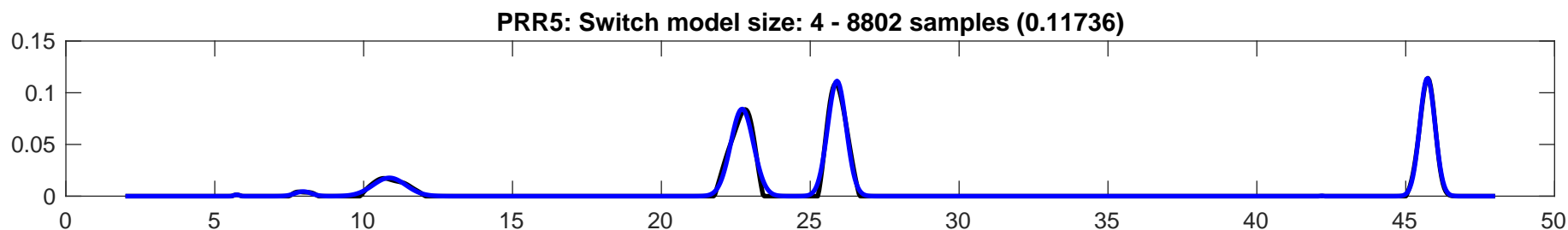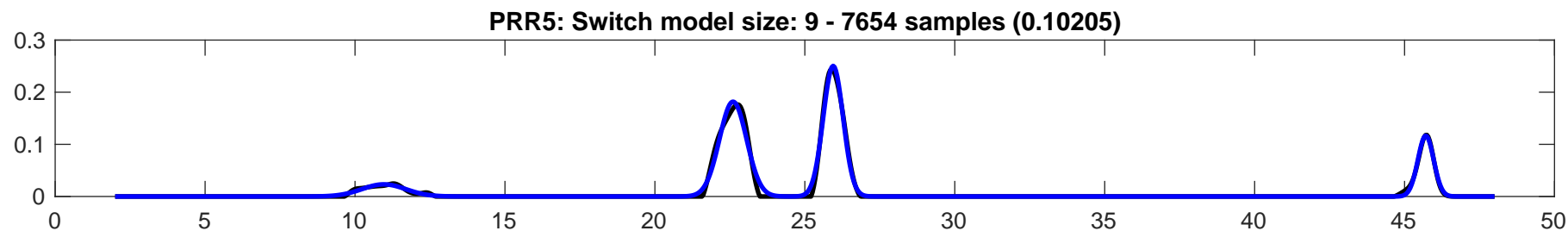

Timescale (hours)

Supplement: Additional file 1 — Toolbox and data. The MATLAB code for running ReTrOS toolbox. This also includes the data used in the illustrative examples and the user manual that provides the instructions on how to run the toolbox. (ZIP 52838 kb) [file 12859_2017_1695_MOESM1_ESM.zip › ReTrOS-master/ReTrOSv5.4/output/clock_genes_mRNA/plots_switch/PRR5_models.pdf]

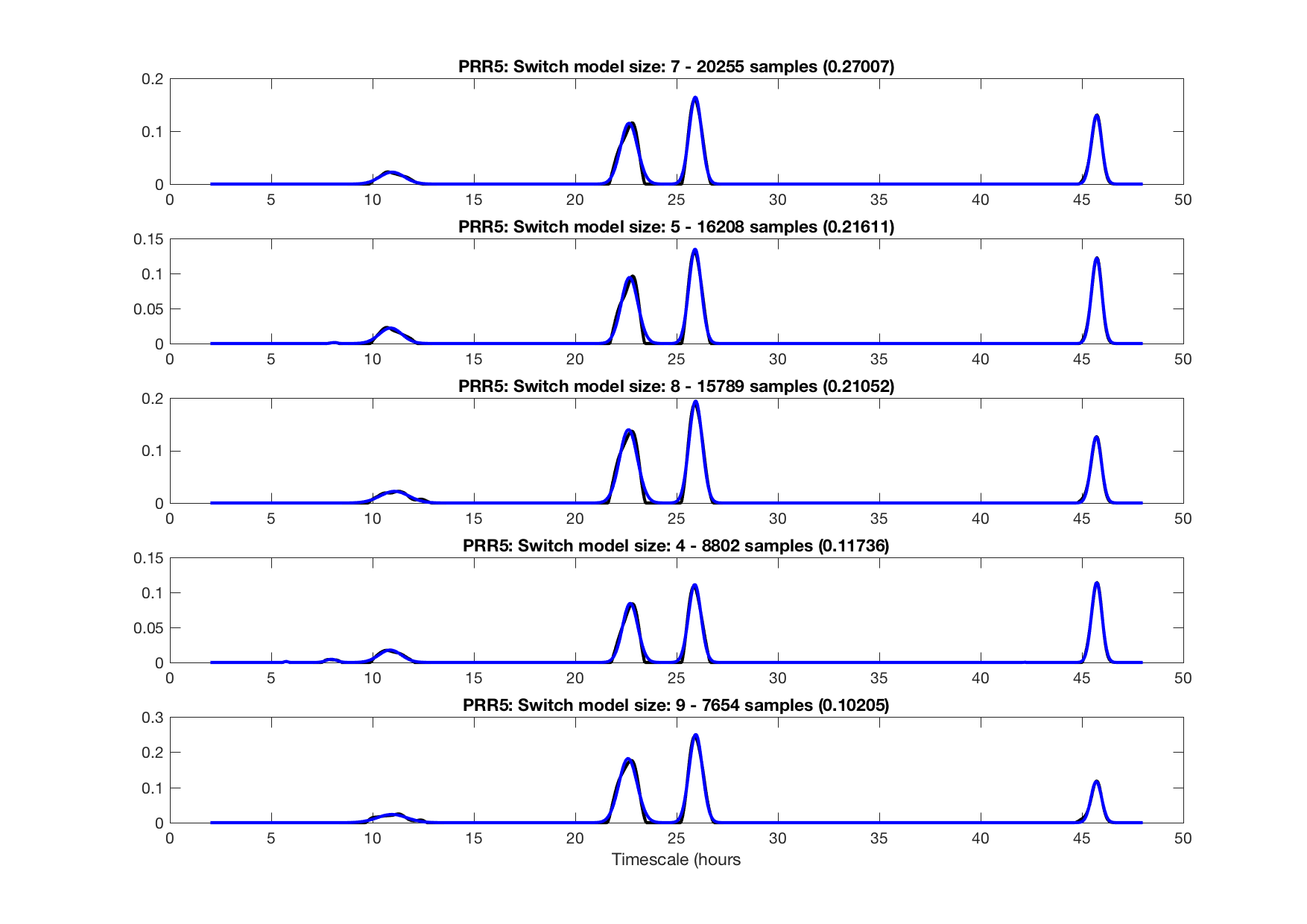

Supplement: Additional file 1 — Toolbox and data. The MATLAB code for running ReTrOS toolbox. This also includes the data used in the illustrative examples and the user manual that provides the instructions on how to run the toolbox. (ZIP 52838 kb) [file 12859_2017_1695_MOESM1_ESM.zip › ReTrOS-master/ReTrOSv5.4/output/clock_genes_mRNA/plots_switch/PRR5_models.png]

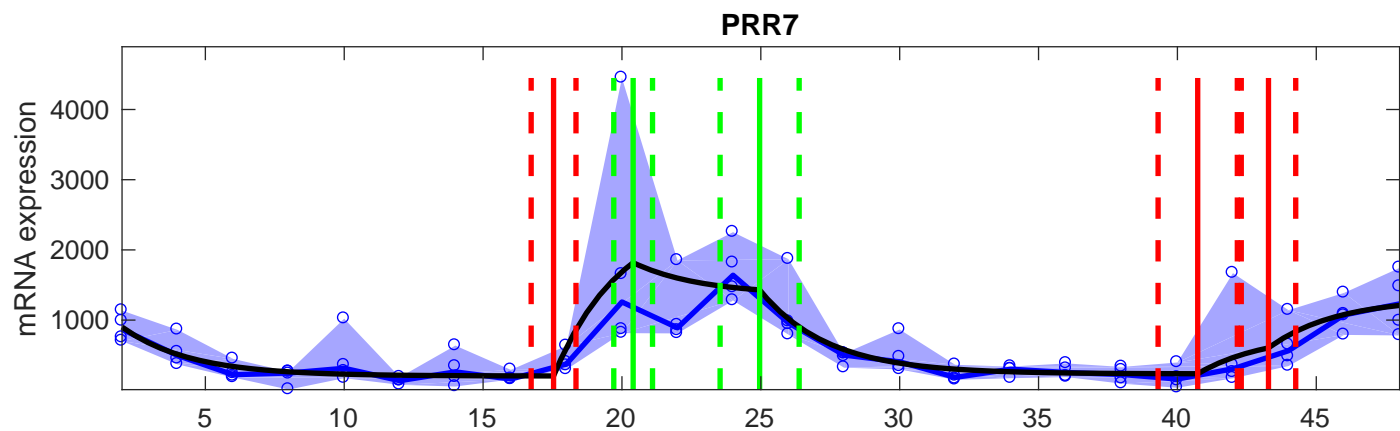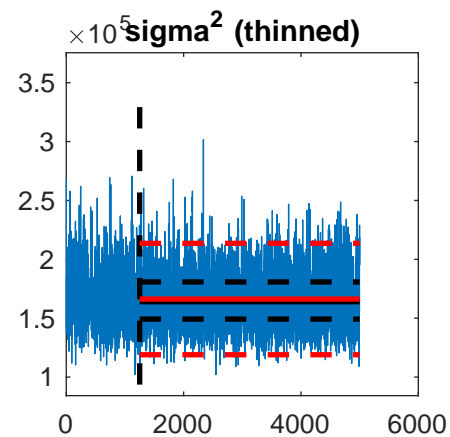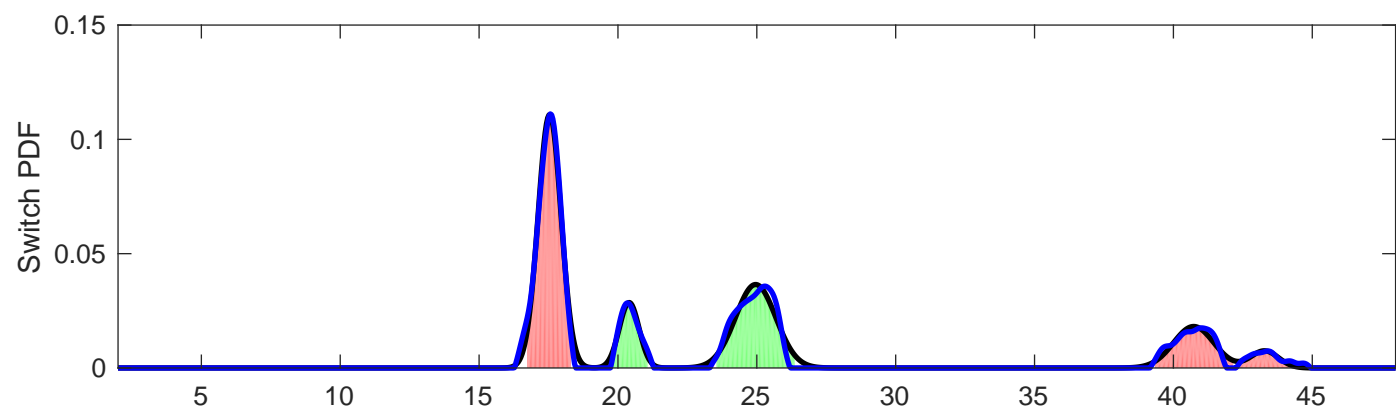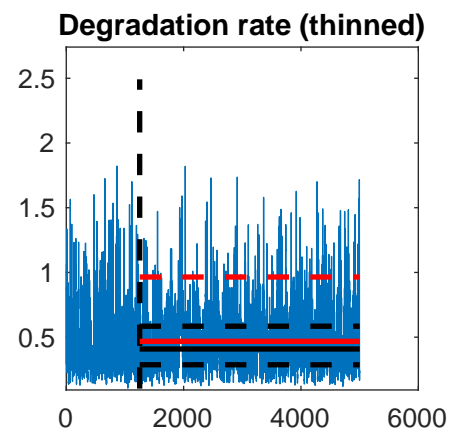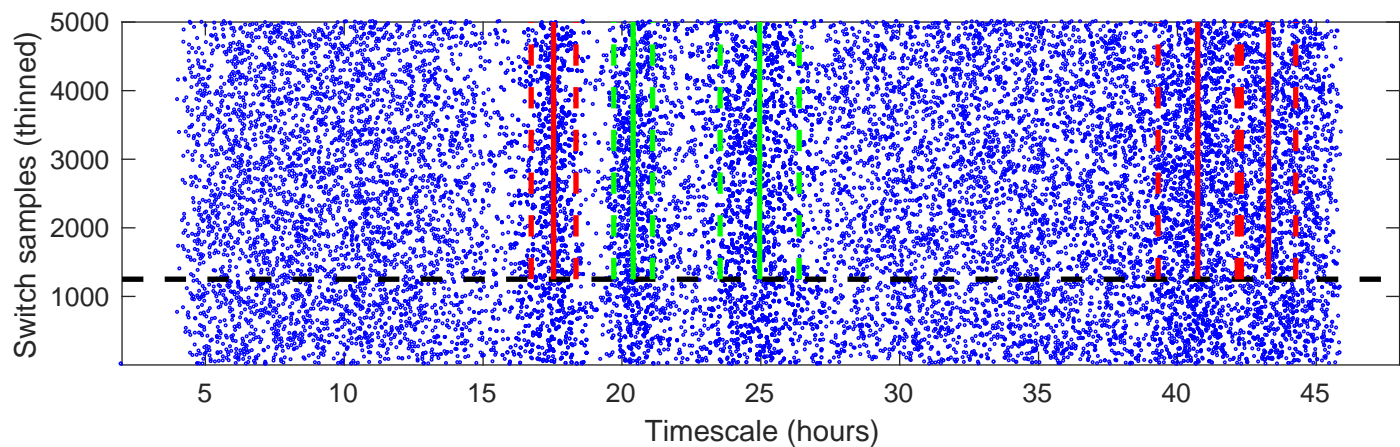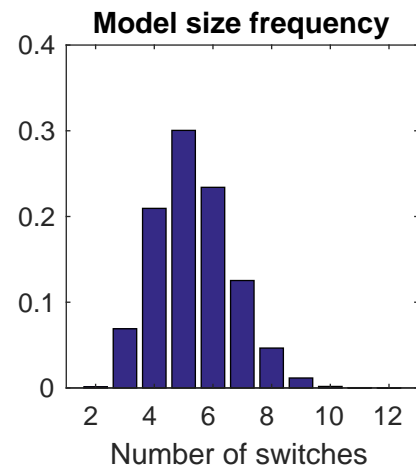

Supplement: Additional file 1 — Toolbox and data. The MATLAB code for running ReTrOS toolbox. This also includes the data used in the illustrative examples and the user manual that provides the instructions on how to run the toolbox. (ZIP 52838 kb) [file 12859_2017_1695_MOESM1_ESM.zip › ReTrOS-master/ReTrOSv5.4/output/clock_genes_mRNA/plots_switch/PRR7.pdf]

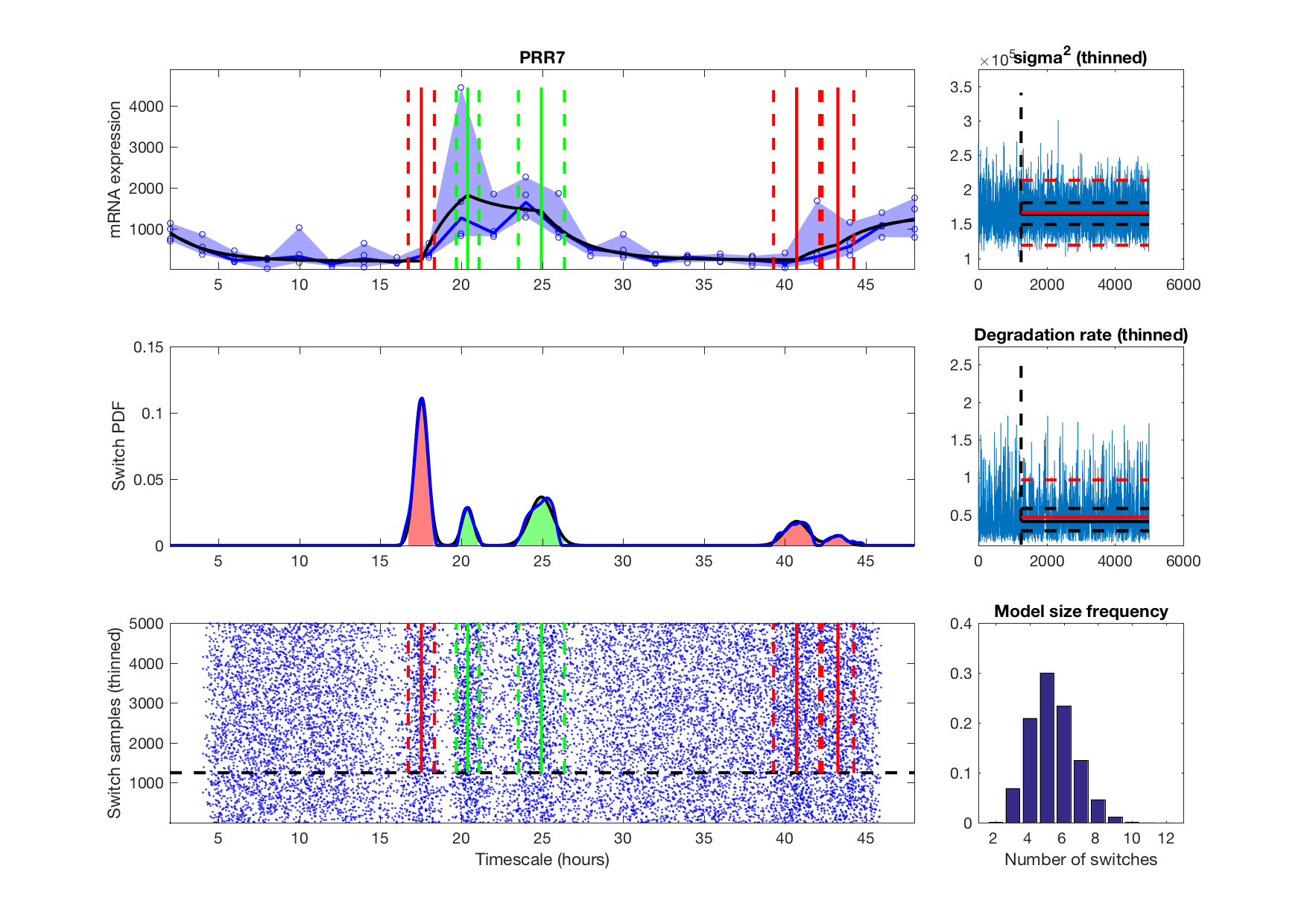

Supplement: Additional file 1 — Toolbox and data. The MATLAB code for running ReTrOS toolbox. This also includes the data used in the illustrative examples and the user manual that provides the instructions on how to run the toolbox. (ZIP 52838 kb) [file 12859_2017_1695_MOESM1_ESM.zip › ReTrOS-master/ReTrOSv5.4/output/clock_genes_mRNA/plots_switch/PRR7.png]

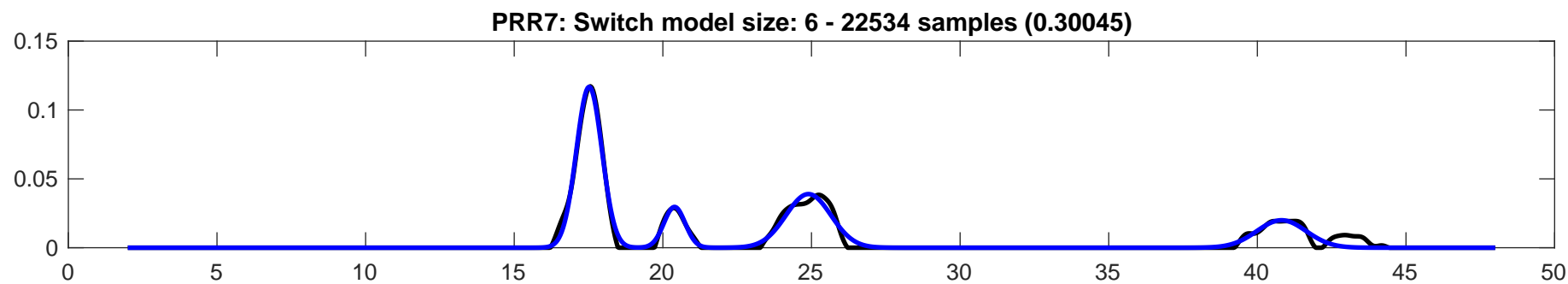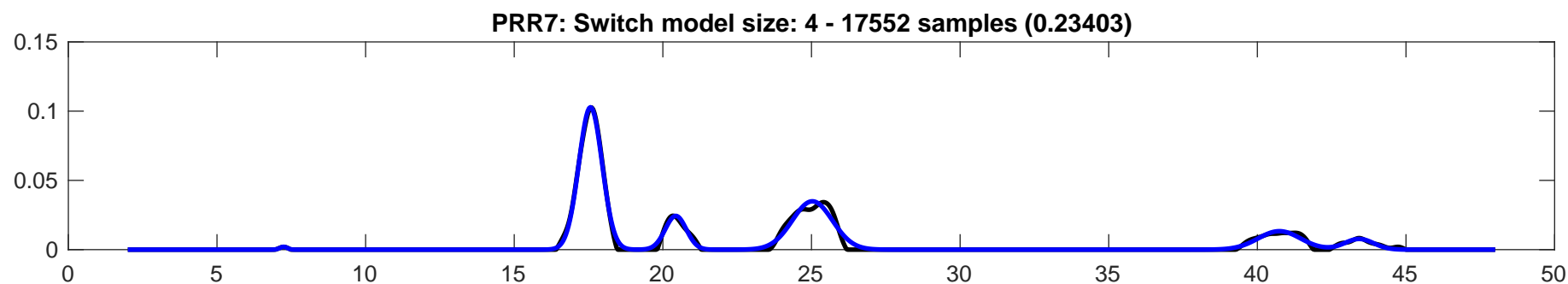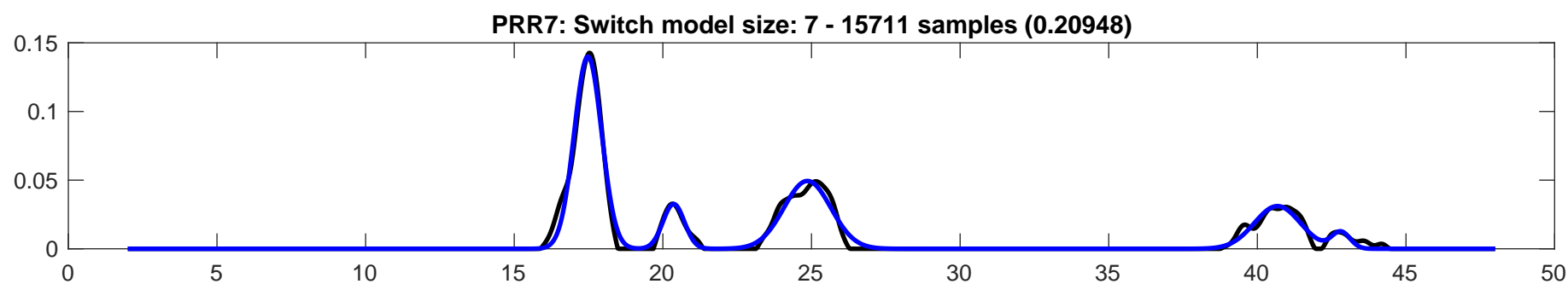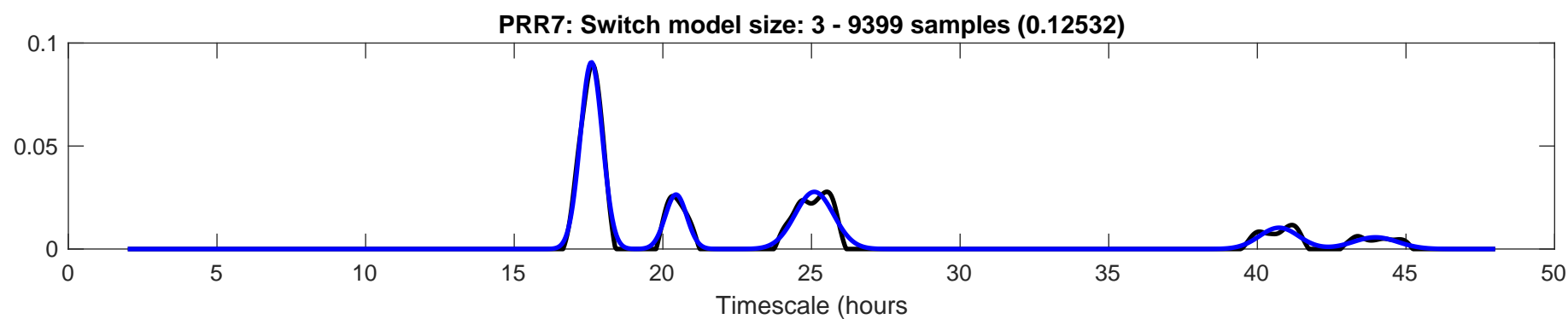

Supplement: Additional file 1 — Toolbox and data. The MATLAB code for running ReTrOS toolbox. This also includes the data used in the illustrative examples and the user manual that provides the instructions on how to run the toolbox. (ZIP 52838 kb) [file 12859_2017_1695_MOESM1_ESM.zip › ReTrOS-master/ReTrOSv5.4/output/clock_genes_mRNA/plots_switch/PRR7_models.pdf]

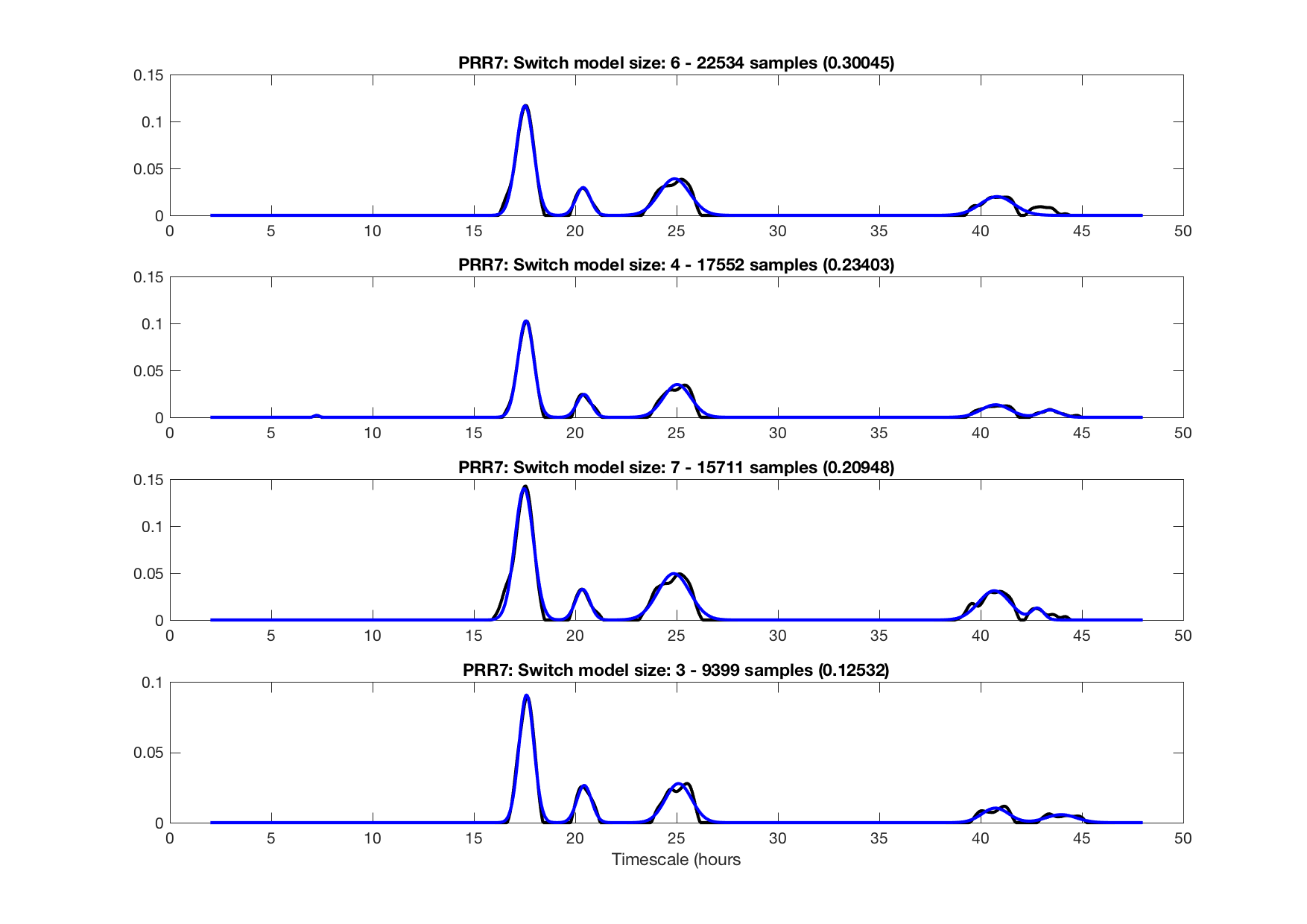

Supplement: Additional file 1 — Toolbox and data. The MATLAB code for running ReTrOS toolbox. This also includes the data used in the illustrative examples and the user manual that provides the instructions on how to run the toolbox. (ZIP 52838 kb) [file 12859_2017_1695_MOESM1_ESM.zip › ReTrOS-master/ReTrOSv5.4/output/clock_genes_mRNA/plots_switch/PRR7_models.png]

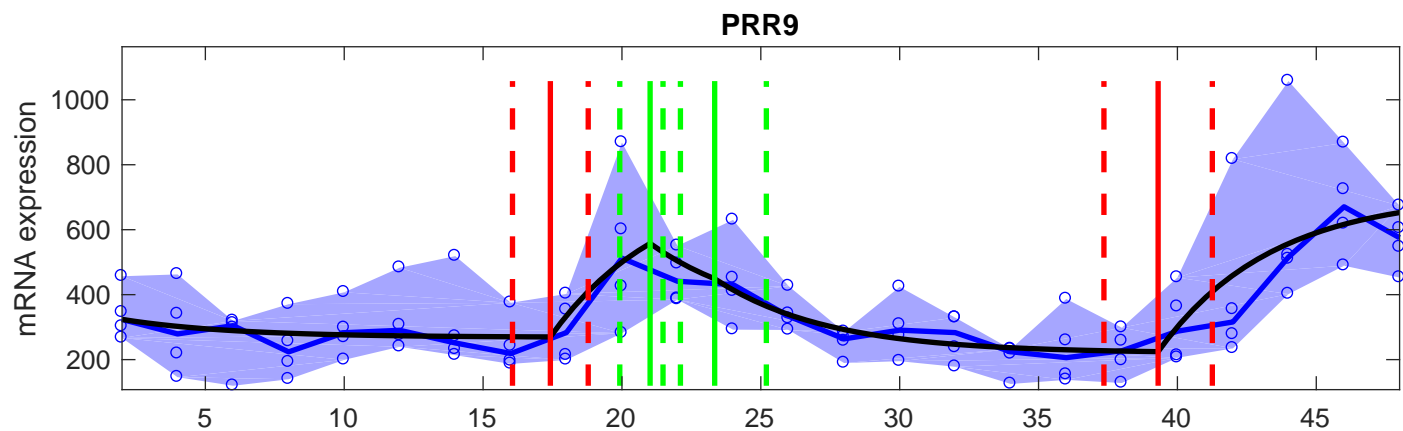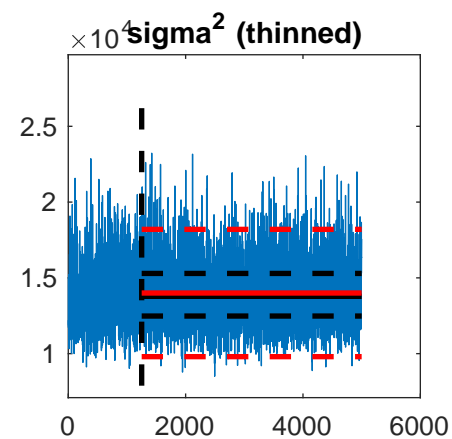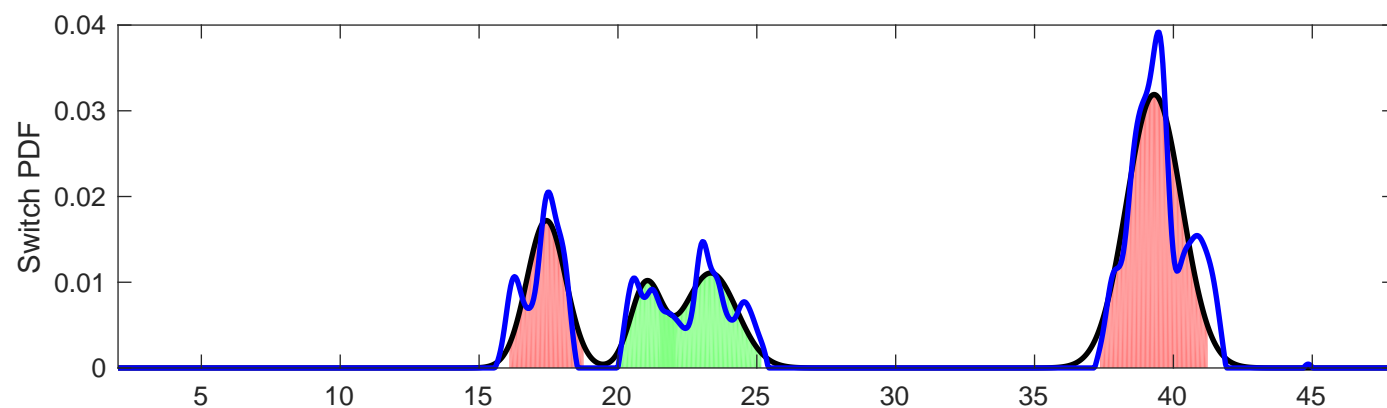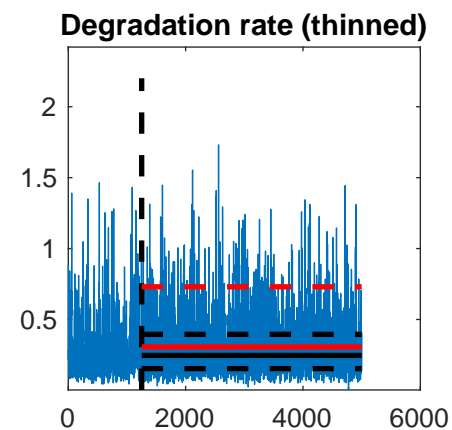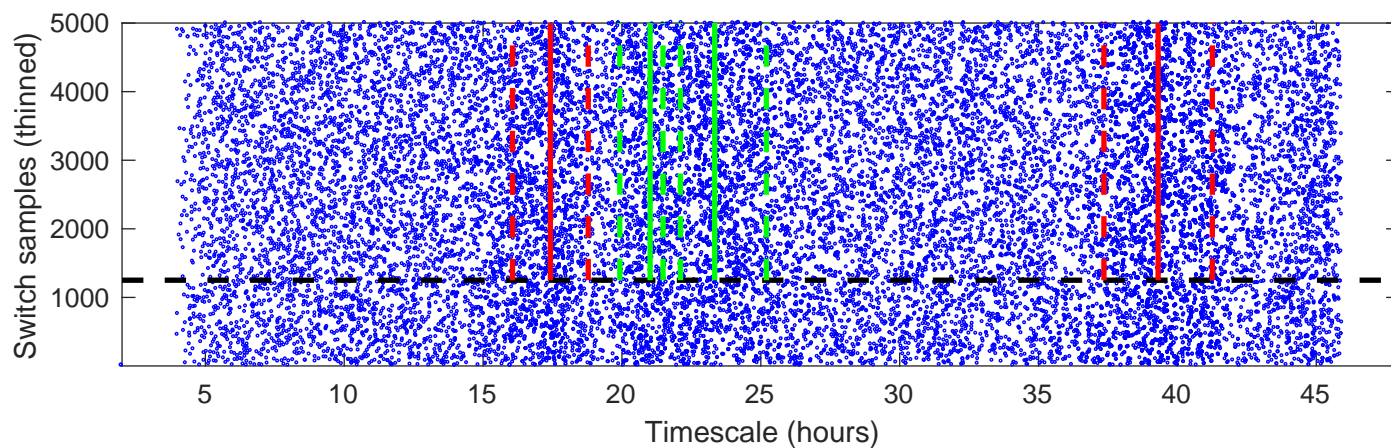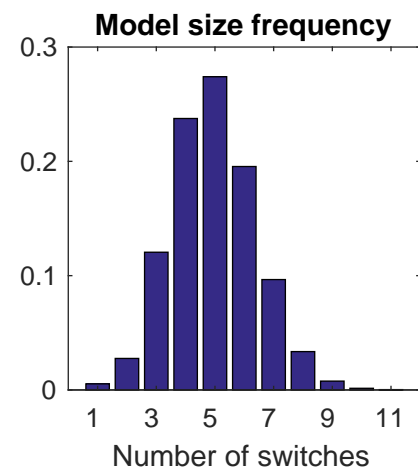

Supplement: Additional file 1 — Toolbox and data. The MATLAB code for running ReTrOS toolbox. This also includes the data used in the illustrative examples and the user manual that provides the instructions on how to run the toolbox. (ZIP 52838 kb) [file 12859_2017_1695_MOESM1_ESM.zip › ReTrOS-master/ReTrOSv5.4/output/clock_genes_mRNA/plots_switch/PRR9.pdf]

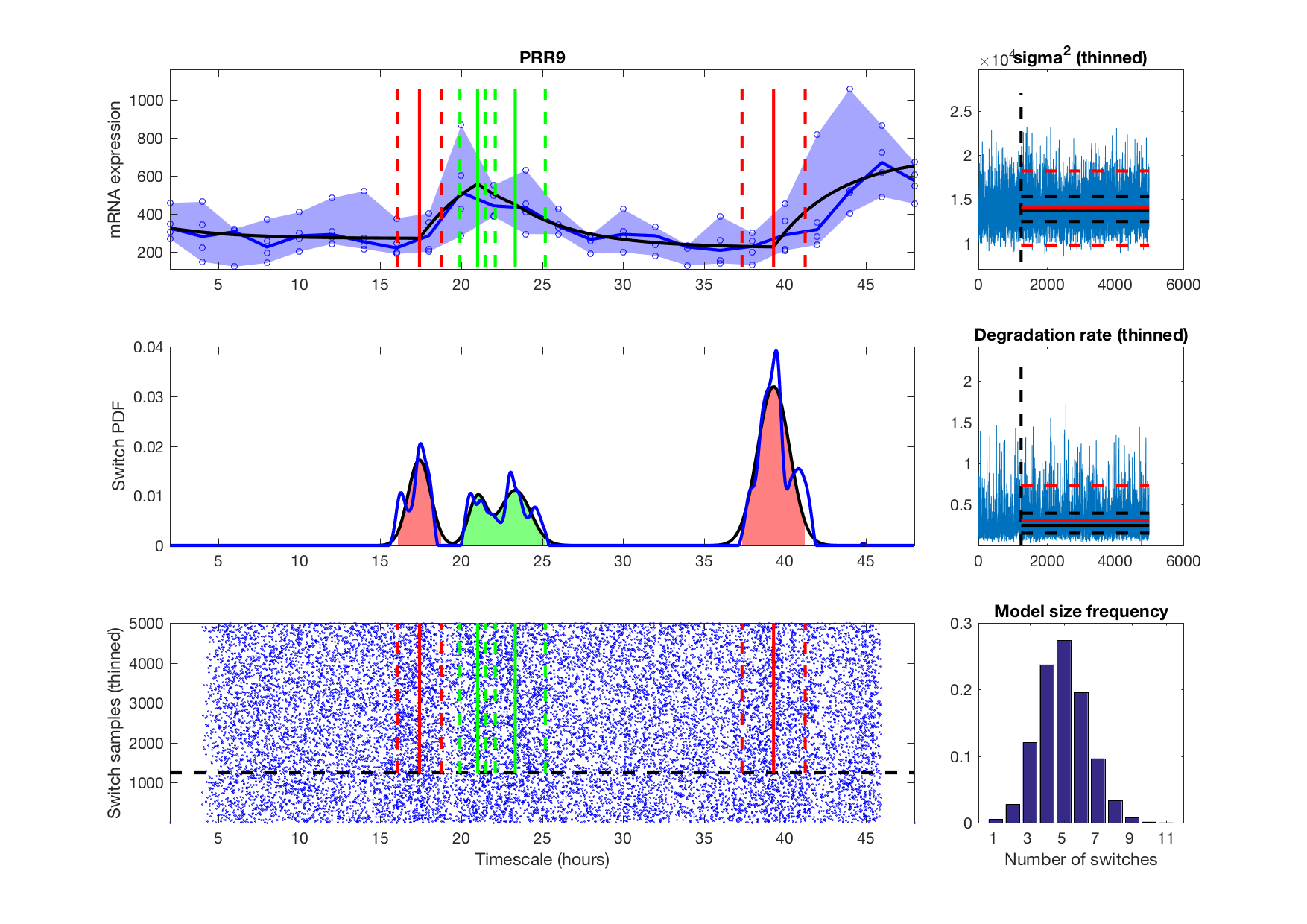

Supplement: Additional file 1 — Toolbox and data. The MATLAB code for running ReTrOS toolbox. This also includes the data used in the illustrative examples and the user manual that provides the instructions on how to run the toolbox. (ZIP 52838 kb) [file 12859_2017_1695_MOESM1_ESM.zip › ReTrOS-master/ReTrOSv5.4/output/clock_genes_mRNA/plots_switch/PRR9.png]

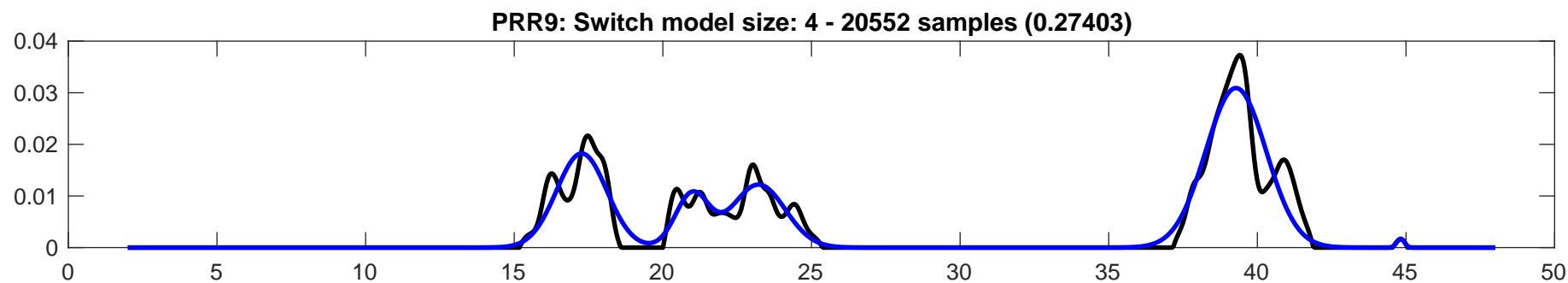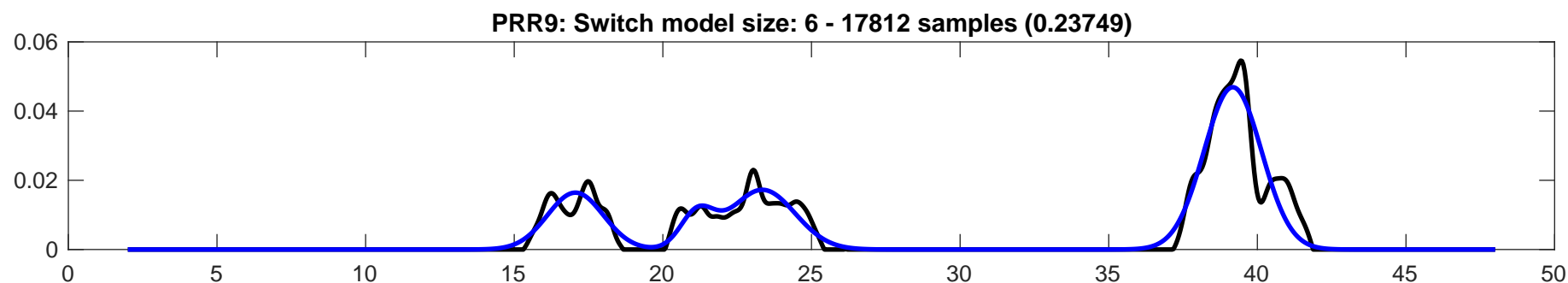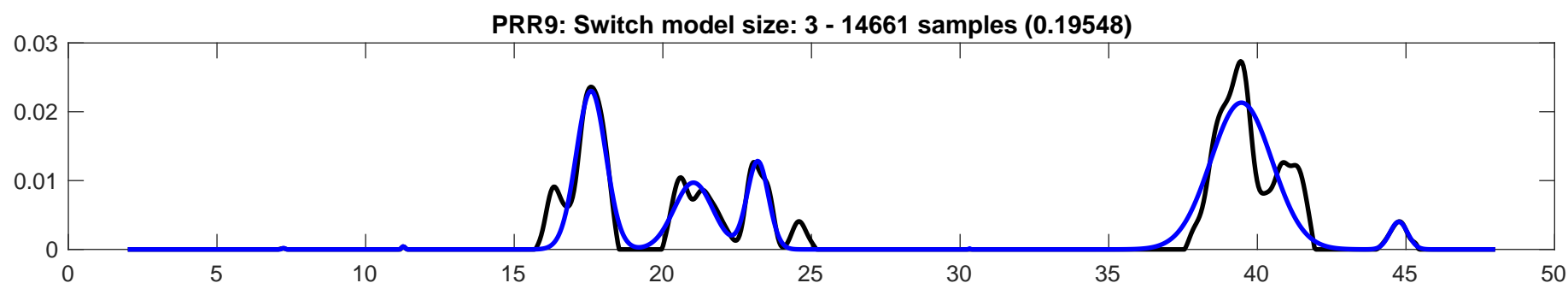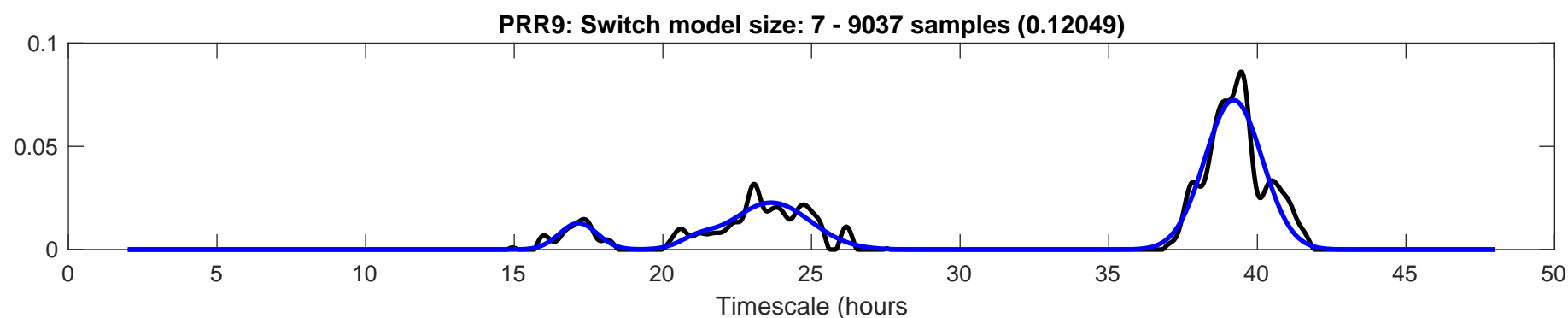

Supplement: Additional file 1 — Toolbox and data. The MATLAB code for running ReTrOS toolbox. This also includes the data used in the illustrative examples and the user manual that provides the instructions on how to run the toolbox. (ZIP 52838 kb) [file 12859_2017_1695_MOESM1_ESM.zip › ReTrOS-master/ReTrOSv5.4/output/clock_genes_mRNA/plots_switch/PRR9_models.pdf]

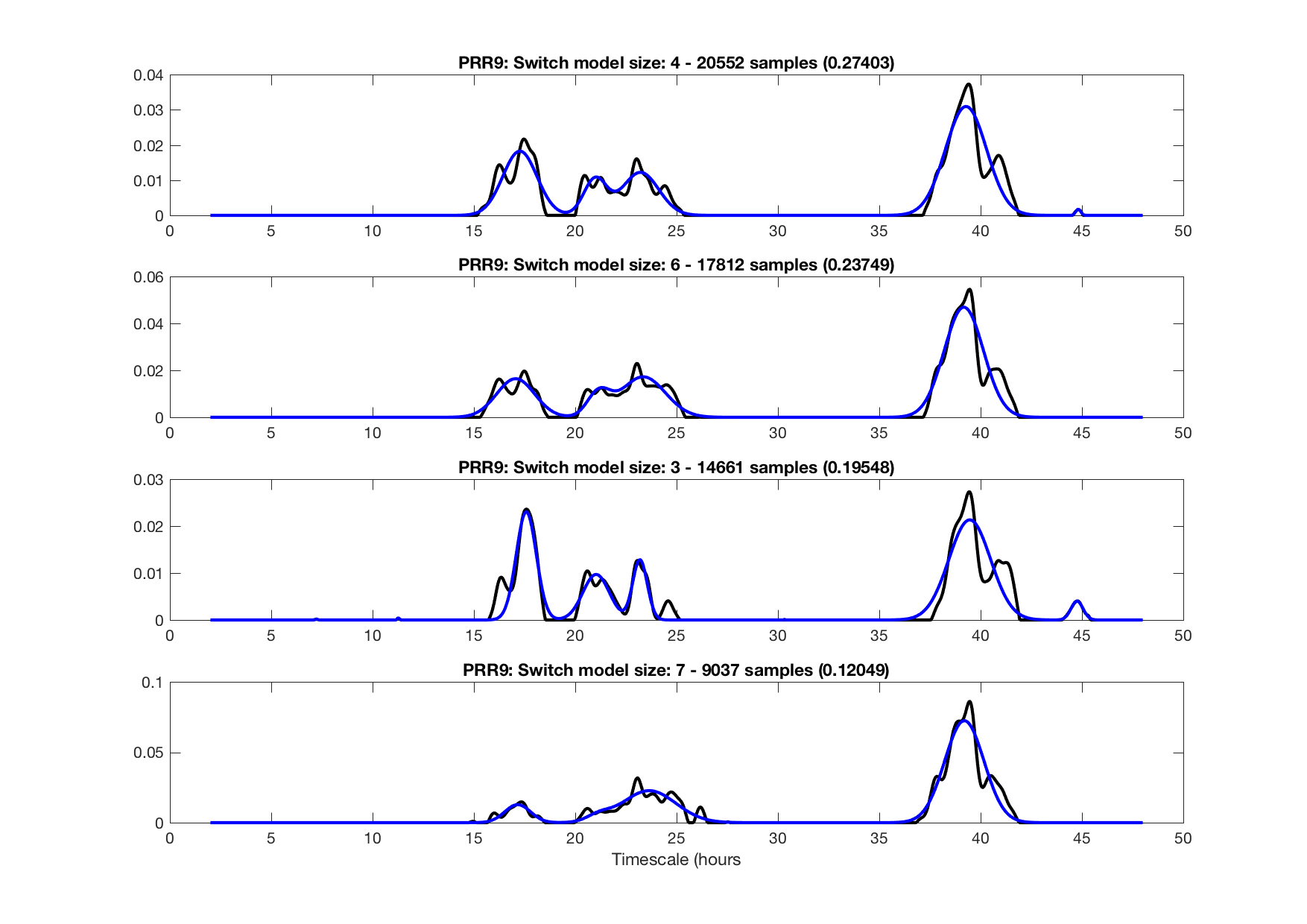

Supplement: Additional file 1 — Toolbox and data. The MATLAB code for running ReTrOS toolbox. This also includes the data used in the illustrative examples and the user manual that provides the instructions on how to run the toolbox. (ZIP 52838 kb) [file 12859_2017_1695_MOESM1_ESM.zip › ReTrOS-master/ReTrOSv5.4/output/clock_genes_mRNA/plots_switch/PRR9_models.png]

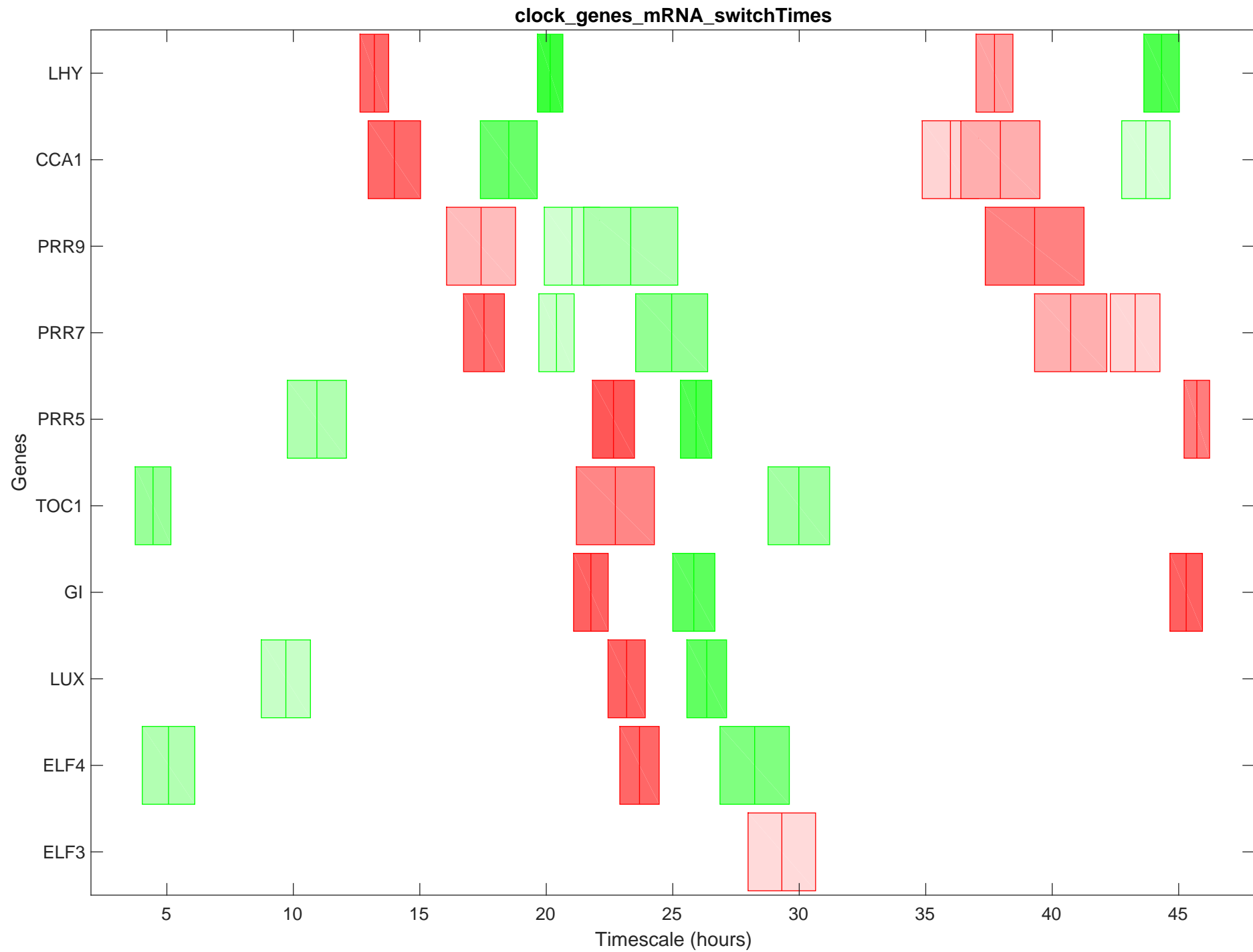

Supplement: Additional file 1 — Toolbox and data. The MATLAB code for running ReTrOS toolbox. This also includes the data used in the illustrative examples and the user manual that provides the instructions on how to run the toolbox. (ZIP 52838 kb) [file 12859_2017_1695_MOESM1_ESM.zip › ReTrOS-master/ReTrOSv5.4/output/clock_genes_mRNA/plots_switch/switchTimes.pdf]

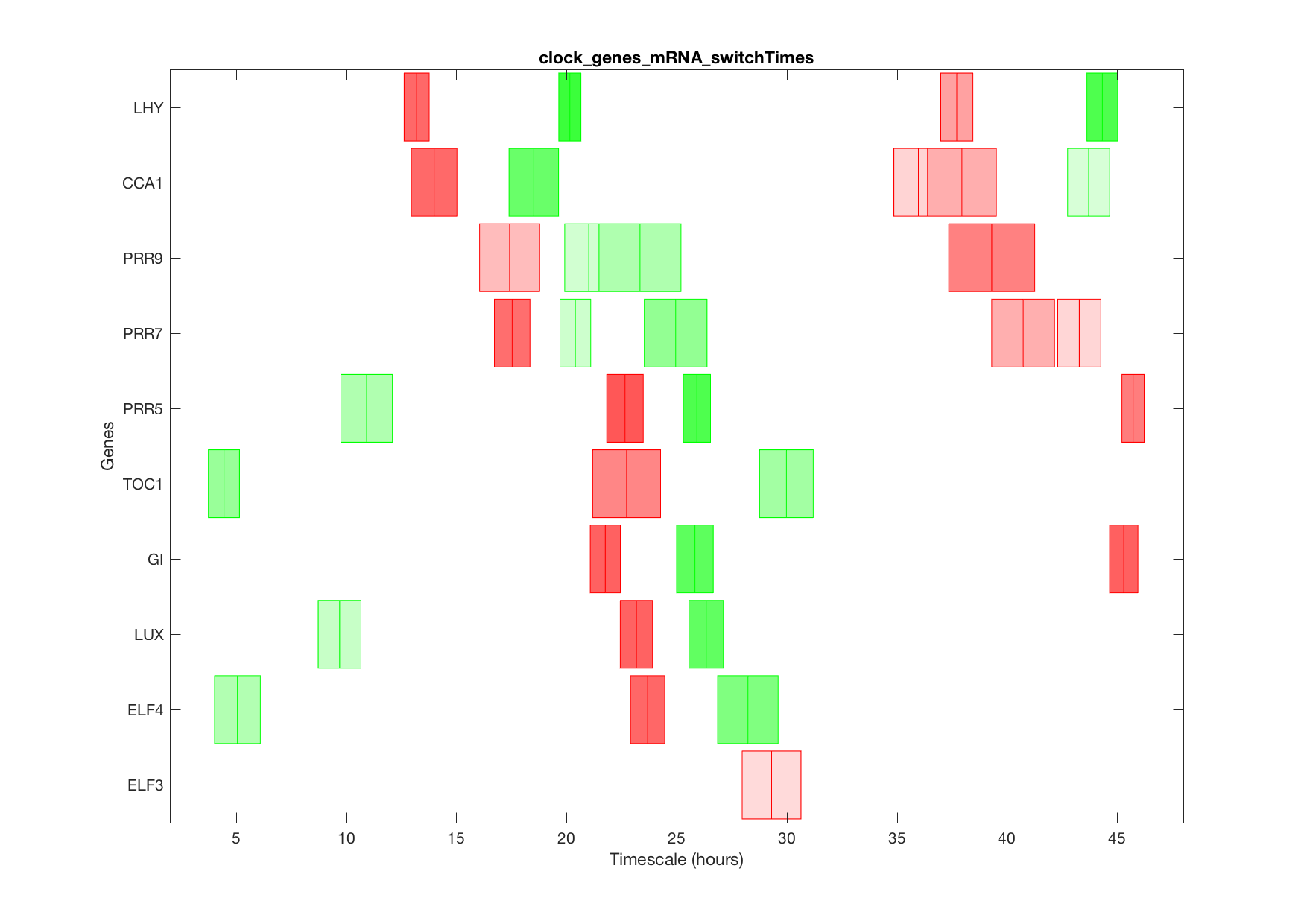

Supplement: Additional file 1 — Toolbox and data. The MATLAB code for running ReTrOS toolbox. This also includes the data used in the illustrative examples and the user manual that provides the instructions on how to run the toolbox. (ZIP 52838 kb) [file 12859_2017_1695_MOESM1_ESM.zip › ReTrOS-master/ReTrOSv5.4/output/clock_genes_mRNA/plots_switch/switchTimes.png]

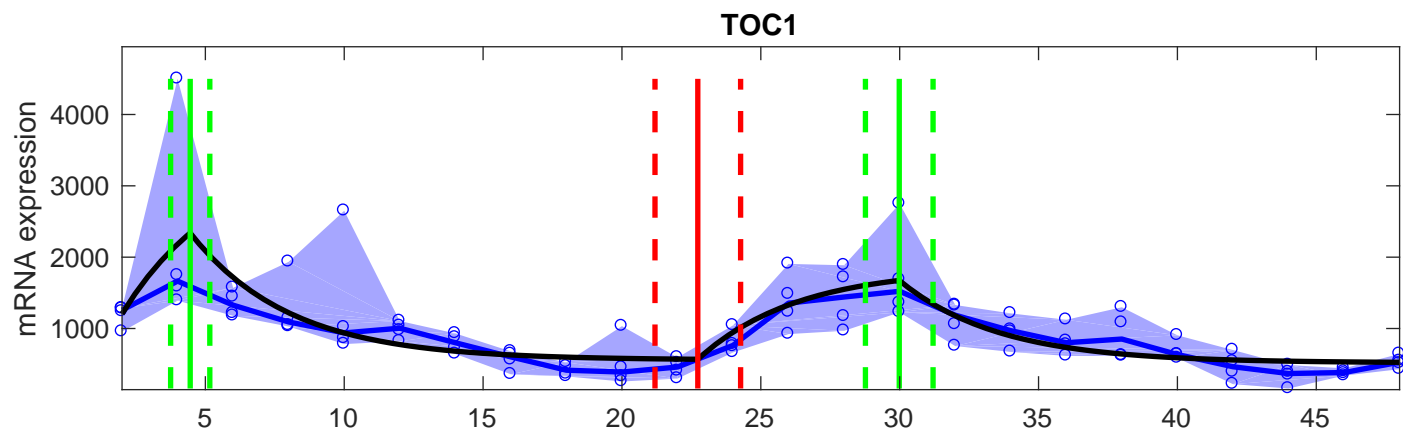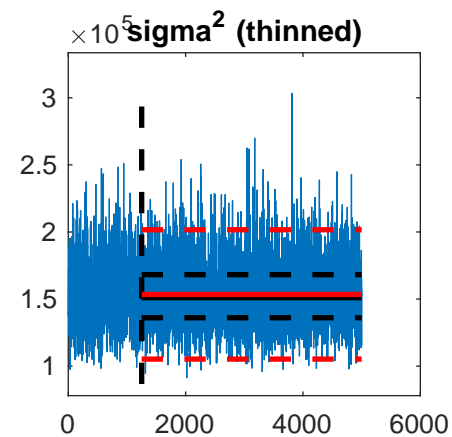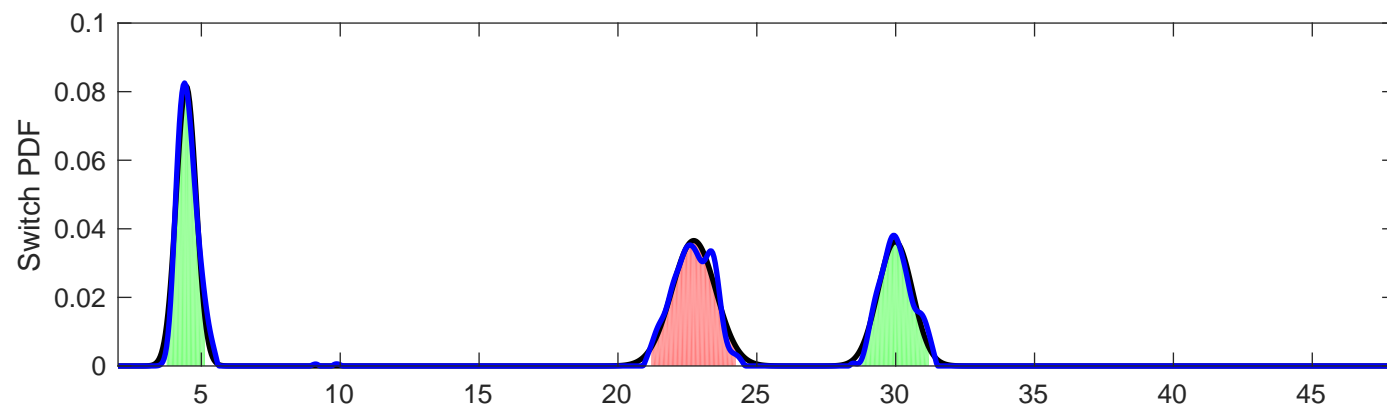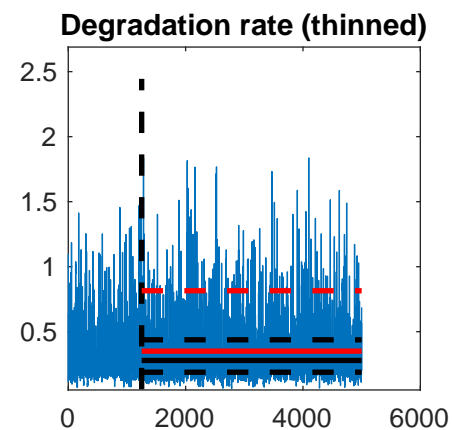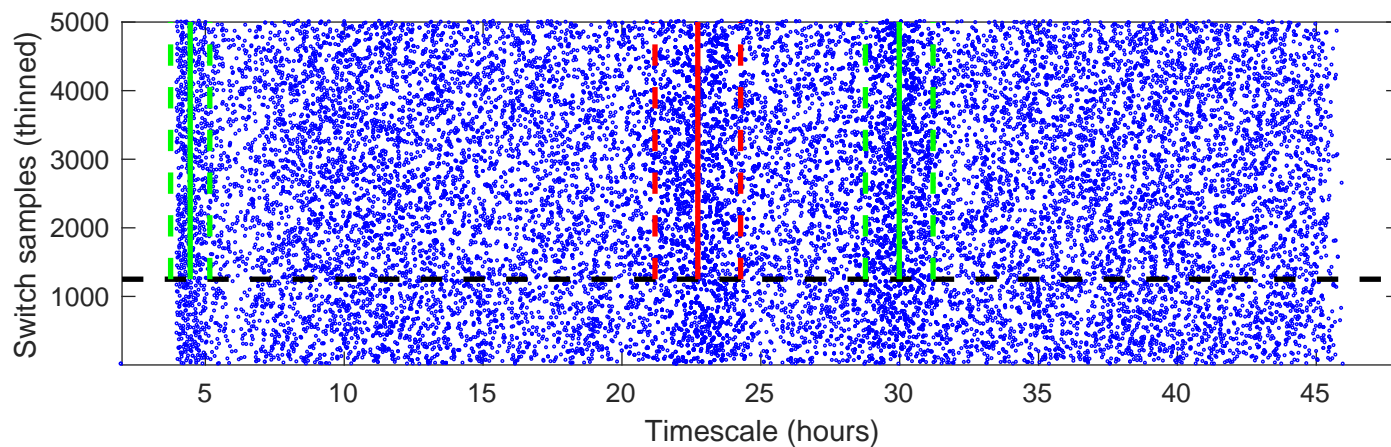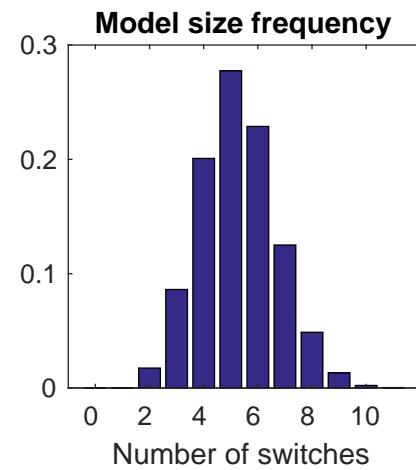

Supplement: Additional file 1 — Toolbox and data. The MATLAB code for running ReTrOS toolbox. This also includes the data used in the illustrative examples and the user manual that provides the instructions on how to run the toolbox. (ZIP 52838 kb) [file 12859_2017_1695_MOESM1_ESM.zip › ReTrOS-master/ReTrOSv5.4/output/clock_genes_mRNA/plots_switch/TOC1.pdf]

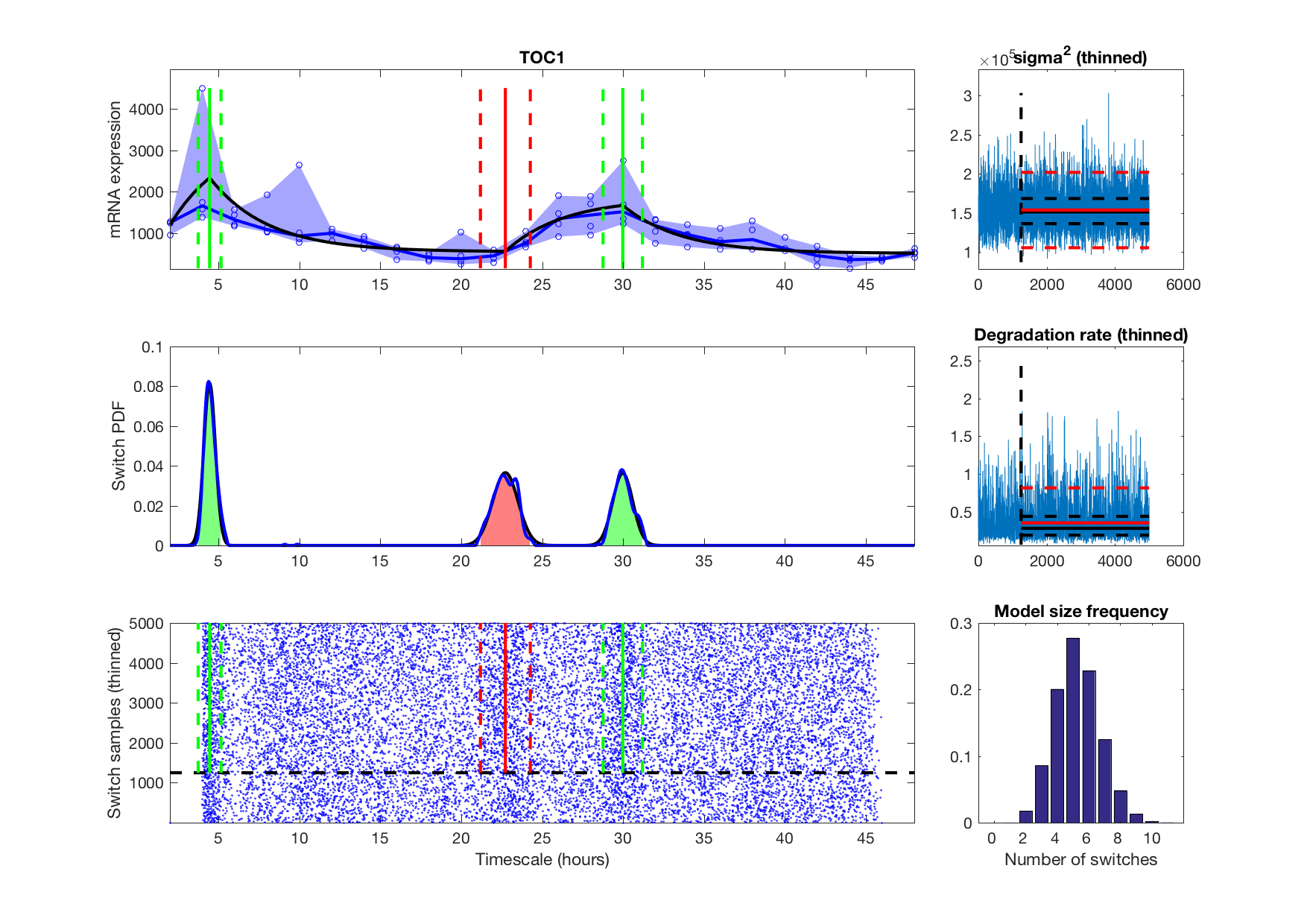

Supplement: Additional file 1 — Toolbox and data. The MATLAB code for running ReTrOS toolbox. This also includes the data used in the illustrative examples and the user manual that provides the instructions on how to run the toolbox. (ZIP 52838 kb) [file 12859_2017_1695_MOESM1_ESM.zip › ReTrOS-master/ReTrOSv5.4/output/clock_genes_mRNA/plots_switch/TOC1.png]

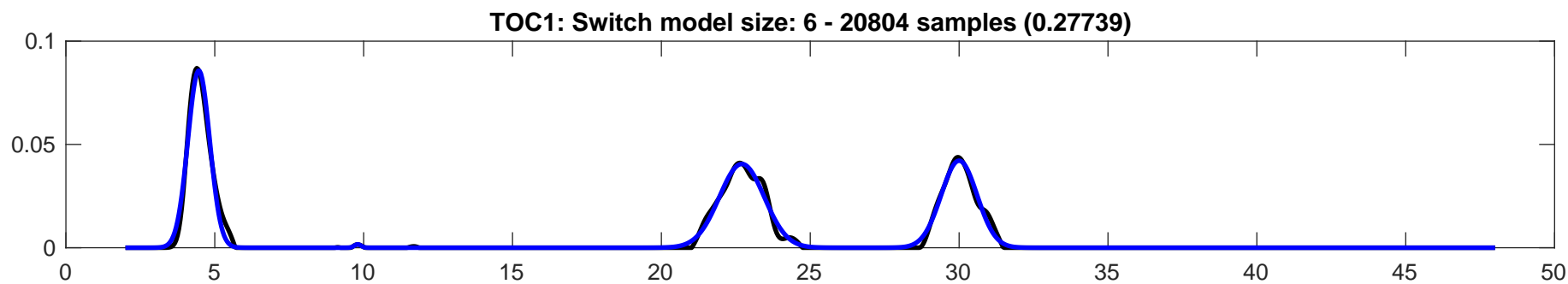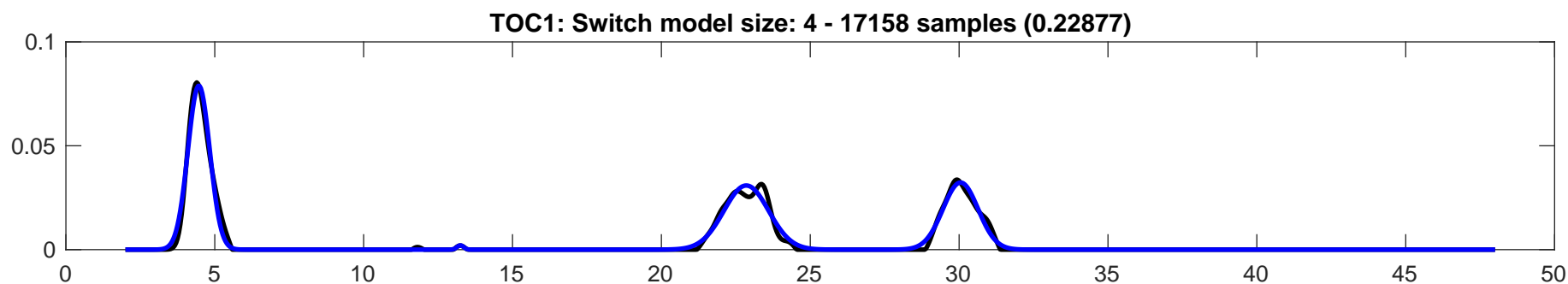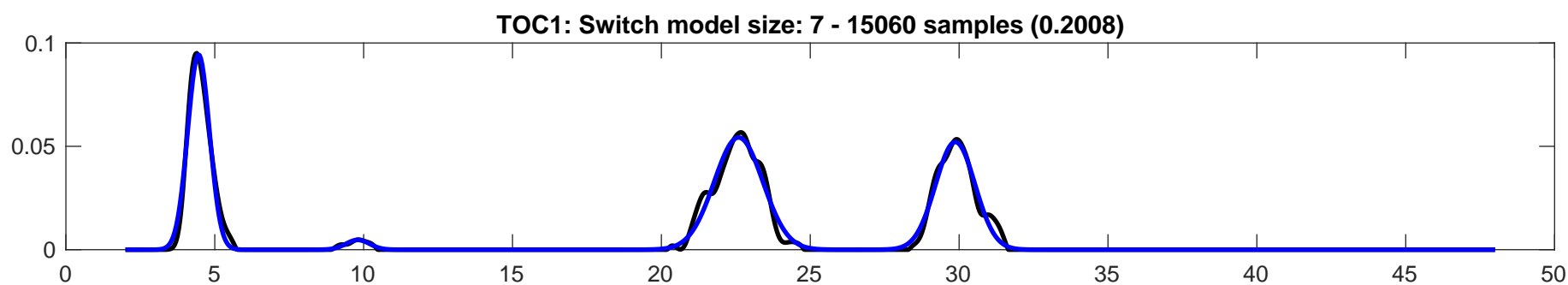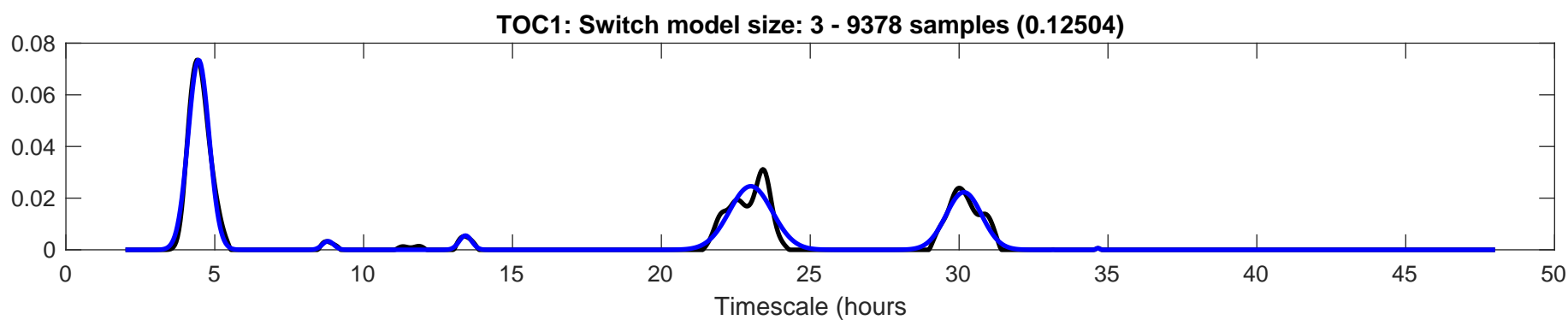

Supplement: Additional file 1 — Toolbox and data. The MATLAB code for running ReTrOS toolbox. This also includes the data used in the illustrative examples and the user manual that provides the instructions on how to run the toolbox. (ZIP 52838 kb) [file 12859_2017_1695_MOESM1_ESM.zip › ReTrOS-master/ReTrOSv5.4/output/clock_genes_mRNA/plots_switch/TOC1_models.pdf]

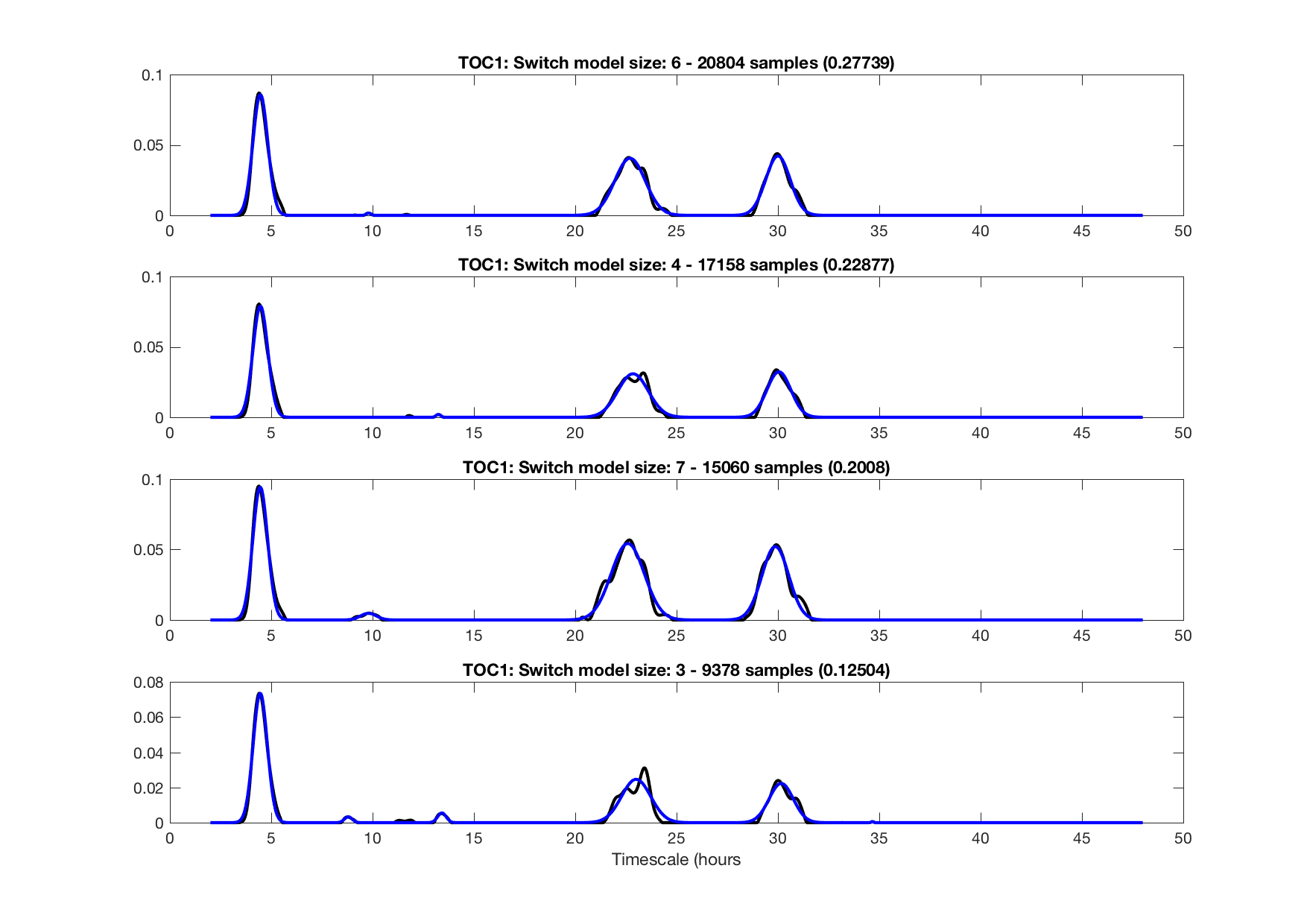

Supplement: Additional file 1 — Toolbox and data. The MATLAB code for running ReTrOS toolbox. This also includes the data used in the illustrative examples and the user manual that provides the instructions on how to run the toolbox. (ZIP 52838 kb) [file 12859_2017_1695_MOESM1_ESM.zip › ReTrOS-master/ReTrOSv5.4/output/clock_genes_mRNA/plots_switch/TOC1_models.png]
